# Abstracts of the 8th Tanzania Health Summit, October 2021: Dodoma, Tanzania. 11-13, October 2021

**DOI:** 10.1186/s12919-022-00231-0

**Published:** 2022-06-02

## A1 Gametocyte composition and malaria parasite transmission

### Victor Mwingira^1^, Eliapendavyo Msuya^1^, William Kisinza^1^, Subam Kathet^2^

#### ^1^Amani Research Centre, National Institute for Medical Research, Tanga, Tanzania; ^2^University of Helsinki, Helsinki, Finland

##### **Correspondence:** Victor Mwingira (victor.mwingira@gmail.com)

**Background**

Youth are challenged globally by sexual and reproductive health problems such as unwanted pregnancy, unsafe abortion and sexually transmitted infections. While family planning methods are a safe and effective way for individuals to responsibly control their sexual and reproductive needs, their use amongst sexually active youth is very low. This contributes to over 4.5 million unwanted pregnancies of which 40% end up as unsafe abortions globally. It is of urgency to understand factors that influence youth to adopt family planning methods to inform future strategies that aim at increasing the use of these modern methods of birth control.

**Objectives**

This study aimed at determining the prevalence and factor associated with utilization of family planning methods among youth in Mwanza, Tanzania.

**Methodology**

A cross sectional analytical study design was conducted among youth aged 15-24 years in selected secondary schools and university Colleges in Nyamagana district, Mwanza from January to March 2018. Participating institutions and the participants were selected using a multistage sampling method. Data was collected using self-administered questionnaires and later analyzed using SPSS version 23. Univariate and multivariable logistic regression was used to analyze factors associated with the use of family planning; with significance considered at p-value<0.05.

**Results**

A total of 349 participants were enrolled in this study. The prevalence of utilization of family planning methods among sexually active youth was 83.2%. Factors associated with FP use were being female (AOR 2.84; CI: 1.05, 7.67) and not having a peer who is using the method (AOR 0.31; 95% CI: 0.12, 0.82). Poor awareness on availability of FP services at nearby facility was found to be significant (cOR 0.38; 95% CI: 0.16-0.90) during crude analysis but become insignificant when adjusted for other factors

**Conclusion**

Majority of sexually active learned youth were utilizing FP methods. Sex and peer pressure were significantly associated with family planning use. Therefore, initiatives for advocating comprehensive sexuality education and strengthening youth friendly health clinics are highly proposed to increase consistent contraceptive use among youth.

## A2 Prevalence and factors associated with utilization of family planning methods among youth in mwanza-tanzania

### Upendo S Safari^1^, Leah A Sanga^1^, Catherine S Massay^1^, Geofrey Sigalla^1^, Rune N Philemon^2^

#### ^1^Sumve School of Nursing, Kilimanjaro Christian Medical University College, Mwanza, Tanzania; ^2^Kilimanjaro Christian Medical University College, Moshi, Tanzania

##### **Correspondence:** Upendo S Safari (uppysafari@gmail.com)

**Background**

Youth are challenged globally by sexual and reproductive health problems such as unwanted pregnancy, unsafe abortion and sexually transmitted infections. While family planning methods are a safe and effective way for individuals to responsibly control their sexual and reproductive needs, their use amongst sexually active youth is very low. This contributes to over 4.5 million unwanted pregnancies of which 40% end up as unsafe abortions globally. It is of urgency to understand factors that influence youth to adopt family planning methods to inform future strategies that aim at increasing the use of these modern methods of birth control.

**Objective**

This study aimed at determining the prevalence and factor associated with utilization of family planning methods among youth in Mwanza, Tanzania.

**Methodology**

A cross sectional analytical study design was conducted among youth aged 15-24 years in selected secondary schools and university Colleges in Nyamagana district, Mwanza from January to March 2018. Participating institutions and the participants were selected using a multistage sampling method. Data was collected using self-administered questionnaires and later analyzed using SPSS version 23. Univariate and multivariable logistic regression was used to analyze factors associated with the use of family planning; with significance considered at p-value<0.05.

**Results**

A total of 349 participants were enrolled in this study. The prevalence of utilization of family planning methods among sexually active youth was 83.2%. Factors associated with FP use were being female (AOR 2.84; CI: 1.05, 7.67) and not having a peer who is using the method (AOR 0.31; 95% CI: 0.12, 0.82). Poor awareness on availability of FP services at nearby facility was found to be significant (cOR 0.38; 95% CI: 0.16-0.90) during crude analysis but become insignificant when adjusted for other factors

**Conclusion**

Majority of sexually active learned youth were utilizing FP methods. Sex and peer pressure were significantly associated with family planning use. Therefore, initiatives for advocating comprehensive sexuality education and strengthening youth friendly health clinics are highly proposed to increase consistent contraceptive use among youth.

## A3 Intrauterine contraceptive device uptake and associated factors among women at haydom hospital, Tanzania

### Catherine Safari Massay^1^, Jane Rogathi^1^, Geofrey Nimrod Sigalla^1^ , Upendo Safari^2^

#### ^1^Kilimanjaro Christian Medical University College, Moshi, Tanzania; ^2^ Sumve School of Nursing, Kilimanjaro Christian Medical University College, Mwanza, Tanzania

##### **Correspondence:** Catherine Safari Massay (cathy.massay@gmail.com)

**Background**

The global population is growing faster than what the world’s resources could support, with Tanzania listed third among African countries with the highest fertility rates. Access to and utilization of effective family planning services to control fertility is now of urgency. Intrauterine contraceptive device (IUCD) - a long-term reversible contraceptive method represents the most cost effective reversible method for preventing unwanted and unintended pregnancy however; its uptake is very low worldwide and accounts to only 1% of all users of modern family planning methods in Tanzania.

**Objective**

This study aimed to determining the uptake of IUCD and its associated factors among women attending Reproductive and Child health Services at Haydom Hospital in Manyara Tanzania.

**Methods**

This was a hospital based cross-sectional analytical study conducted at Haydom Lutheran Hospital from January 2018 to April 2018. A sample of 347 women of reproductive age was enrolled in this study. Structured questionnaire was employed to gather quantitative and data were analyzed using SPSS Version 23.0.

**Results**

The prevalence of IUCD uptake was 5%. Factors influenced the use of IUCD were the level of knowledge about IUCD [AOR 4.99, 95%CI: 1.24-20.07], positive perception about IUCD [AOR 6.31, 95%CI: 1.39-28.77], marital status of being widowed or divorced [AOR 173.53, 95%CI: 6.11-4930.37] and being a farmer when compared to other formal employment [AOR 0.07, 95%CI: 0.02-0.33].

**Conclusion**

The use of intrauterine contraceptive devices was low in the study area. Participants’ occupation, marital status, knowledge and perceptions were the major factors of uptake of IUCD methods. Fear of side effects and negative perceptions were considered to be as barrier to use among those who have never used IUCD.

## A4 Reliability of visual assessment for diagnosis of neonatal jaundice among neonates of black descent: a cross-sectional study from Tanzania

### Ikunda Dionis

#### University of Dodoma, Dodoma, Tanzania

##### **Correspondence:** Ikunda Dionis (dionisikunda@gmail.com)

**Background**

Jaundice is common among neonates and if untreated can lead to kernicterus. Diagnosing of jaundice in neonates using Kramer’s method (visual assessment) is considered user friendly in resource limited areas. However, there are conflicting finding on reliability of the Kramer’s method in diagnosis of neonatal jaundice (NJ) particularly of black descent. Therefore, this study aimed to determine diagnostic accuracy of Kramer’s method in comparison with total serum bilirubin (TSB) test in diagnosis of NJ among neonates of black descent in Tanzania.

**Methods**

A cross-sectional study was conducted between June and July 2020 at Muhimbili National Hospital (MNH) in Dar es Salaam Tanzania. A total of 315 neonates were recruited consecutively. In each neonates’ jaundice was assessed by using Kramer’s method and TSB test. A 2 X 2 table was created for determination of sensitivity, specificity, positive predictive values (PPV), negative predictive value (NPV), positive and negative likelihood ratios(+LR/-LR) and diagnostic accuracy (effectiveness). Cohen kappa (κ) was used to analyze the agreement between Kramer’s method and TSB. Association between independent variables and presence of jaundice were assessed using chi-square test and the p < 0.05 was considered to be statistical significance.

**Results**

The prevalence of NJ was 49.8% by Kramer’s method and 63.5% by TSB.The Sensitivity, Specificity, PPV, and NPV of the Kramer’s method were 70.5%, 86.1%, 88.8%, and 62.6%, respectively. The +LR and –LR were 5.07 and 0.34 respectively. The diagnostic accuracy of the Kramer’smethod was 76.1%. There was a moderate agreement between Kramer’s method and TSB results (κ= 0.524, P<0.001). No significance relationship between the independent variables and presence of NJ.

**Conclusion**

Kramer’s method was found to be inefficient in detecting NJ among neonates of black descent. However, it can be used as a predictor of NJ and whenever available invasive techniques should be applied.

## A5 Breast cancer and associated factors in sub-saharan africa - literature review

### Neema R Urio^1^, Ngo Van Toan^1^, Geofrey N Sigalla^2^, Elizabeth Watternberg^3^

#### ^1^Hanoi Medical University, Hanoi, Vietnam; ^2^Marie Stopes, Dar es salaam, Tanzania; ^3^University of Minnesota, Minneapolis, United States of America

##### **Correspondence:** Neema R Urio (neema.marindo@gmail.com)

**Background**

Breast cancer is the second most common cancer in women all over the world. It is among the most diagnosed disease among women, both in high resource countries and low resource countries. Although the incidence of breast cancer in Sub-Saharan African countries is low, the rate of death is high. To create new thrust for governments and stakeholders’ preventive interventions, understanding risk factors, community awareness and accessibility to screening of breast cancer in Sub-Saharan Africa is crucial. This study was therefore aimed at describing the status and associated factors of breast cancer in Sub-Saharan Africa from 2008-2017.

**Methodology**

Literature review was conducted in search engines for papers published on breast cancer from January 2008 to December 2017. All articles conducted in Sub-Saharan Africa and published in English were included. Specific search terms were used. Results were summarized from the best evidence based on the study quality and the study objectives. Screened articles and reviews are described through text and presented in figures and tables.

**Results**

PRISMA flow analysis resulted in 32 studies which were included in the final qualitative synthesis, from 339 records initially identified through database search. The incidence of breast cancer in sub-Saharan Africa increased annually by 4.9% in Zimbabwe, 3.7% in Uganda, 6.5% Mozambique and 4.3% in South Africa. Mortality rates reported to increase due to lack of screening programs for early detection, myths, and poverty. Factors associated with breast cancers were reproductive, lifestyle, environmental and genetic factors.

**Conclusion**

In Sub- Saharan countries, education to women about the early detection and treatment of breast cancer, establishment of new equipped cancer treatment centers and breast cancer education programs will be the most important interventions to reduce incidence and mortality of breast cancer. Policy makers should plan and prioritize prevention and control of breast cancer.

## A6 Evaluation of mechanisms to improve mobile phone surveys for non communicable diseases surveillance in tanzania

### John William, Farida Hassan, Irene Petro, Frank Kagoro

#### Ifakara Health Institute, Morogoro, Tanzania

##### **Correspondence:** John William (williamj@ihi.or.tz)

**Background**

NCDs morbidity and mortality are rising at an alarming rate in Tanzania. A clear mapping of their burden and trends is paramount. It’s vital to obtain an easy, user-friendly, and updatable methodology for data collection.

With an increased number of mobile phone subscriptions in Tanzania and its usefulness, opportunities can be exploited focusing on mobile phones as an intervention in different public health issues. A more frequent collection of NCDs risk factors data using mobile phones could address gaps in NCDs surveillance.

**Objectives**

The general objective of the study is to adapt and assess the feasibility, quality, and validity of mobile phone surveys (MPS) for collecting information on NCDs risk factors.

**Methodology**

This study involved mixed-method study designs, qualitative methods to assess local perceptions and preferences. NCDs’ risk factors questionnaire were used and sent randomly via short message services (SMS) and interactive voice responses (IVR) in randomized sub-studies to assess key study outcomes of interest. Thematic framework analysis and STATA version 14 were used in the analysis.

**Results**

The majority of the MPS respondents were aged between 18-29 years, more males compared to females, from urban compared to rural. The number of complete interviews was higher followed by partial interviews and refusal. Overall, the completion rate was higher compared to refusal rates in both study arms.

Illiteracy was reported as a barrier to the SMS surveys. Overall participants reported timing in administering MPS and the use of incentives after completion will influence completion.

**Conclusion**

Mobile phone surveys can be used as a feasible tool for the surveillance of NCDs in developing countries like Tanzania. Consideration of users perceptions and preferences is critical for ease and successful optimization. Exploration of relative advantages and disadvantages and costs associated with each modality is paramount.

## A7 Impact and acceptability of the safe delivery app among skilled birth attendants in mpwapwa district, dodoma

### John William, Mary Ramesh, Donat Shamba

#### Ifakara Health Institute, Morogoro, Tanzania

##### **Correspondence:** John William (williamj@ihi.or.tz)

**Background**

Tanzania is among the countries with the highest maternal and neonatal mortality in the world. The majority of maternal deaths are due to direct obstetric complications, which could be avoided if deliveries were assisted by a skilled birth attendant.

Low-cost mHealth solutions, such as Safe Delivery App (SDA), can support skilled birth attendants and midwives in managing obstetric emergencies, by providing access to evidence-based guidelines.

**Objectives**

The overall objective of the study was to assess the feasibility of using SDA for improving the provision of care for maternal and newborn health in Mpwapwa district, in Dodoma Tanzania.

**Methodology**

Mixed method study involving quantitative and qualitative techniques of data collection was used. 56 health workers were trained on the use of SDA. A Key Feature Questionnaire (KFQ) was used to assess health workers' practical application of knowledge based on clinical scenarios at baseline and end-line. A total of 15 IDI’s were conducted with health workers and 2 FGDs with recently delivered mothers.

**Results**

There is an increased level of knowledge of 7% in various topics in maternal and newborn health after the use of SDA for seven months. It was observed that 86% of users at baseline used the SDA weekly, with weekly use increasing to 94% at the end-point. Videos were the most used feature of the App. Health workers regarded post-partum haemorrhage to be the most useful topic. Healthcare workers reported to become more confident and felt better skilled at handling emergencies which reduced the number of referrals.

**Conclusion**

It was observed there was an upward trend in both knowledge, acceptability and usage of the SDA. Although the assessment on the use of SDA was for a short period of 7-months, the results are promising and provide insights for building a case for national scale implementation.

## A8 Challenges of water unavailability on sanitation in informal settlement

### Zamzam Kibao, Opportuna Kweka

#### University of Dar es Salaam, Dar es Salaam, Tanzania

##### **Correspondence:** Zamzam Kibao (zkhelef@yahoo.com)

**Background**

Water availability is crucial for the survival of every human being. In order to ensure good health and better sanitation, the availability of safe water is inevitable. Therefore, the information of this study informs the government about the current situation of water availability in the informal settlement.

**Objectives**

The study specifically investigated issues of water availability situation in the informal settlement, the challenges of water unavailability on sanitation and the role played by water and sanitation actors – both government and private actors.

**Methodology**

The study employed mixed research design. In data collection both qualitative and quantitative method used. Qualitative methods include in-depth interview and Focus Group Discussion method. Quantitative method was mainly in the form of household survey. Qualitative data analyzed through content analysis while quantitative data analyzed through SPSS version 23.

**Results**

In Tabata ward only 22.1% access water from DAWASA tap at home, others access water from private water actors due to public water unavailability. Majorities do not access tap water at home due to poor infrastructure in terms of housing structure and water infrastructure also area is hazardous, especially along Bonde Msimbazi. Water unavailability bring the following challenges on sanitation; use of unsafe water: people use contaminated water from private water actors as an alternative to safe tap water, use little water than needed in washing 44.2%, bathing 22.7%, and toilet cleaning 33.1%. Postponing use of water and toilet cleaning: activities postponed include laundry, bathing and sometimes toilets would not be cleaned for 2-3 days.

**Conclusion**

Postpone toilet cleaning to save water for basic activities like cooking. The government plays a great role in provision of safe water but does not meet the demand of people. To reduce the challenges of water unavailability on sanitation, the study recommends to reduce the cost of water connection and improving the infrastructure for both water and sanitation service.

## A9 Intimate partnerships and impact on hiv seropositivity status disclosure between men and women

### Tusajigwe Erio^1^, Eileen Moyer^2^, Josien de Klerk^3^

#### ^1^Amsterdam Institute for Global Health and Development, University of Amsterdam, National Institute of Medical Research, Tanzania; ^2^Amsterdam Institute for Global Health and Development (AIGHD), University of Amsterdam (UvA); ^3^Amsterdam Institute for Global Health and Development (AIGHD)

##### **Correspondence:** Tusajigwe Erio (t.erio@aighd.org)

Disclosing HIV seropositivity status continues to be unfavorable to most women in Tanzania who have been diagnosed with HIV. Public health practitioners advocate for HIV status disclosure to increase adherence and limit transmission among couples with sero-discordancy status. With the recent introduction of the test and treat policy in Tanzania implementing the treat all strategy, HIV status disclosure is imperative. In the Tanzanian context, a woman earns her respect in a community by being in a conjugal relationship/being married, making marriage highly valued with women. Although there is a body of literature explaining the effect of disclosure on intimate partnerships, the effect of intimate partnerships on disclosure between men and women has not been well-established. This paper elucidates how men and women value intimate partnership differently and how it impacts disclosure in HIV discourse. We conducted qualitative research between 2016 -2018 among people living with HIV in Shinyanga urban and rural. The findings revealed that HIV-positive women were more concerned with protecting their intimate relationships compared to men. The type of relationship dictated a woman’s decision on disclosure. HIV seropositivity was perceived as a barrier to marriage, making disclosure unfavorable to most women compared with men. Both young and adult women desired to be married/remarry or be in a permanent relationship. Their readiness and decision to disclose their HIV status were directly affected by their aspiration of being married. Men were more concerned with finances and sex. Most men reported having disclosed their HIV status to their partners and some family members compared to women. The findings suggest that interventions should develop support groups for HIV-infected women and men to increase awareness on the effective use of ART for limiting transmission, therefore, disclosure should not be a barrier to experiencing intimate relationships.

## A10 Invitro filtration efficiency for selected face masks to bacteria with a size smaller than sars-cov-2 respiratory droplet

### Maryam Amour, Hussein Mwanga, George Bwire

#### Muhimbili University of Health and Allied Sciences, Dar es salaam, Tanzania

##### **Correspondence:** Maryam Amour (maryam.a.amur@gmail.com)

**Background**

A severe acute respiratory syndrome coronavirus 2 (SARS-CoV-2) can be transmitted between people through respiratory droplets (>5-10 μm in diameter).

**Objectives**

We conducted an invitro experiment to determine the filtration efficiency for the selected cotton cloth, medical face masks and N95 respirators) to bacteria with 0.5-1.5 μm in diameter.

**Methodology**

A suspension was prepared using normal saline (NaCl) and bacteria (Staphylococcus aureus and Escherichia coli) and maintained at a turbidity of 0.5 MacFarland. The suspensions was put in a 100ml plastic spray bottle (with an approximated 250 μl and flow rate of 31.5 ft 3 /min per spray) and then a single spray was applied to the test mask. Within 0 and after 4 hrs after spraying, a swab was streaked on a CLED media then incubated for 48 hours at 37 o C in ambient air. Bacterial filtration efficiency (BFE) was determined as the proportions of colony forming units (CFUs) between the test and control mask.

**Results**

We found a BFE of 100% and 99% for medical and double layer cotton cloth masks, respectively. While observing other measures such as physical distance (at least 1.5m apart) and regular hand washing.

**Conclusion**

While further studies including clinical trials are needed, preliminary findings promise the use of at least two-layers of cotton cloth for face coverings in public settings to prevent the spread of the SARS-CoV-2 droplets from the wearer.

## A11 Role of human pegivirus infections in whole sporozoite vaccination and controlled human malaria infection

### Anneth-Mwasi Tumbo^1^, Maximillian Mpina^1^, Florence Milando^1^, Said Jongo^1^, Salim Abdulla^1^, Omar Juma^1^, Ali Hamad^1^, Elizabeth Nyakarungu^1^, Mwajuma Chemba^1^, Ali Mtoro^1^ , Kamaka Ramadhan^1^, Ally Olotu^1^, Damas Makweba^1^, Stephen Mgaya^1^, Claudia Daubenberger^1^, Marcel Tanner^2^, Tobias Schindler^2^, Jean-Pierre Dangy^2^, Nina Orlova-Fink^2^, Jose Raso Bieri^3^, , Kenneth Stuart^4^, Matthieu Perreau^5^, Jack Stapleton^6^, Stephen Hoffman^7^

#### ^1^Ifakara Health Institute, Morogoro, Tanzania; ^2^Swiss Tropical and Public Health Institute, University of Basel, Basel, Switzerland; ^3^Equatorial Guinea Malaria Vaccine Initiative, Malabo, Equatorial Guinea; ^4^Seattle Children hospital, Seattle, United States; ^5^Centre Hospitalier Universitaire Vaudois, Lausanne, Switzerland; ^6^University of Lowa, Lowa City, United States of America; ^7^Sanaria, Maryland, United States of America

##### **Correspondence:** Anneth-Mwasi Tumbo (atumbo@ihi.or.tz)

**Background**

Co-infections are among many factors associated with immune dysfunction and sub-optimal vaccination outcomes. Chronic, asymptomatic viral infections can contribute to the modulation of vaccine efficacy through various mechanisms. Human Pegivirus-1 (HPgV-1) persists in immune cells potentially modulating immune responses. We investigated whether Pegivirus infection influences vaccine-induced responses and protection in African volunteers undergoing whole P. falciparum sporozoites-based malaria vaccination and controlled human malaria infections (CHMI).

**Methods**

HPgV-1 prevalence was quantified by RT-qPCR in plasma samples of 96 individuals before, post vaccination with PfSPZ Vaccine and after CHMI in cohorts from Tanzania and Equatorial Guinea. The impact of HPgV-1 infection was evaluated on (1) systemic cytokine and chemokine levels measured by Luminex, (2) PfCSP-specific antibody titers quantified by ELISA (3) asexual blood-stage parasitemia pre-patent periods and parasite multiplication rates (4) HPgV-1 RNA levels upon asexual blood-stage parasitemia induced by CHMI.

**Results**

The prevalence of HPgV-1 was 29.2% (28/96) and sequence analysis of the 5' UTR and E2 regions revealed the predominance of genotypes 1, 2 and 5. HPgV-1 infection was associated with elevated systemic levels of IL-2 and IL-17A. Comparable vaccine-induced anti-PfCSP antibody titers, asexual blood-stage multiplication rates and pre-patent periods were observed in HPgV-1 positive and negative individuals. However, a tendency for higher protection levels was detected in the HPgV-1 positive group (62.5%) compared to the negative one (51.6%) following CHMI. Overall, HPgV-1 viremia levels were not significantly altered after CHMI.

**Conclusions**

HPgV-1 infection did not alter PfSPZ Vaccine elicited levels of PfCSP-specific antibody responses and parasite multiplication rates. Ongoing HPgV-1 infection appears to improve to some degree protection against CHMI in PfSPZ-vaccinated individuals. This is likely through modulation of immune system activation and systemic cytokines as higher levels of IL-2 and IL17A were observed in HPgV-1 infected individuals. CHMI is safe and well tolerated in HPgV-1 infected individuals

## A12 Knowledge on tuberculosis among key and vulnerable population (kvp) in Mbeya and Songwe region, Tanzania

### Christina Manyama, Elimina Siyame, Willyhelmina Olomi, Chacha Mangu

#### Mbeya Medical Research Centre, National Institute for Medical Research, Mbeya, Tanzania

##### **Correspondence:** Christina Manyama (cmanyama@nimr-mmrc.org)

**Introduction**

TB ranks as the leading cause of death from infectious disease worldwide. Several factors account for an individual to acquire TB infection however HIV epidemic has increased the reactivation of latent TB infection to 5 to 15 percent in HIV/TB co infection. In Tanzania HIV epidemic is quite generalize with more concentration in Key and Vulnerable population (KVP). Due to the overlap nature of HIV and TB, this survey assessed the knowledge, attitude and practice of KVP regarding TB disease and HIV

**Method**

A survey study in Mbeya and Songwe regions to explore knowledge about TB among KVP attending the Mobile Diagnostic Testing and Care sites. A total of 249 respondents who fulfilled the criteria for KVP were enrolled and administered a semi structured questionnaire on TB. Data were analyzed using STATA 14.

**Results**

Data were collected from 249 respondents of which Females were 152(61.04%), male 95 (38.15%), aged 15-24 were 68 (27.31%), 25-34 were 116 (46.59%,35 and above 65 (26.10%), Primary level of education were 104(41.94%), secondary education were 73 (29.44%) and professionals were 44 (17.74%).Data showed that knowledge level on TB differ from one education group to another ,illiterate had poor knowledge compared to primary education (aOR=0.08, 95% CI (0.02-0.25, p= 0.001), Primary education level compared to professionals (aOR=7.81, 95% CI (1.67-36.48), p-0.009 are associated with decrease of the good knowledge towards TB and when primary was compared to secondary education (aOR=1.74, 95% CI 0.78-3.87, p-0.176) the difference was not statistical significant. When comparing age groups of 14-24 years with 20-34 years, the later had better knowledge on TB (aOR=0.92,95% CI 0.42-2.00 p=0.826).

**Conclusion**

Participants involved in this survey were well informed about TB, however knowledge differ between groups which might lead to some misconception and stigmatization.

## A13 Prevalence and risk factors of hypertension in children in Dar es salaam

### Peter Kibacha

#### Muhimbili National Hospital, Dae es Salaam, Tanzania

##### **Correspondence:** Peter Kibacha (tahecap@yahoo.com, kibacha2001@yahoo.co.uk)

**Background**

The increase in magnitude of hypertension in children is of serious concern. Hypertension has been shown to start in early life and to track into adulthood. Detecting Children with hypertension and prehypertension will aid early intervention and reduction of associated morbidity and mortality. There are several reports showing gaps between children from different social economic backgrounds.

**Methodology**

A descriptive crossectional study was conducted between August 2015 to August 2017 to determine the prevalence and risk factors for hypertension among primary school children in Dar es Salaam, Tanzania. Eight hundred and ninety two (892) children aged between four and eighteen years were recruited. Structured questionnaires were used to obtain socio-demographic characteristics of the children and their parents or guardians. Data were analyzed using Statistical Package for Social Sciences (SPSS) version 16.0.

**Results**

In this study 892 children 442(49.6%) females and 450 (50.4%) males were enrolled in the study.

Of recruited children 445(54.4%) were from public schools. Two hundred and thirty children (25.8%) were hypertensive. One hundred and thirty nine children (15.6%) were overweight, and 306(34.3%) were obese. Female children were more obese (37.7%) compared to male children (30.9%).Among 230 who had elevated BP, 132 had high BMI.

**Conclusion**

The prevalence of hypertension among primary school children in Dar es Salaam was found to be high (25.8%). Higher prevalence of hypertension 28.5% was seen in children from private primary schools compared to 23.4% prevalence of hypertension in children prom public primary schools. There was an increased association between hypertension in children with obesity, sedentary lifestyle and children those from high social economic status. It is suggested to use the findings of this study as markers for increased likelihood of elevated blood pressure in children. As such it is recommended that children in primary schools should be evaluated for obesity and its related risk factors to hypertension.

## A14 Harm reduction services in Tanzania mainland

### Peter Kibacha

#### Muhimbili National Hospital, Dae es Salaam, Tanzania

##### **Correspondence:** Peter Kibacha (tahecap@yahoo.com, kibacha2001@yahoo.co.uk)

**Introduction and methodology**

This project was conducted in between September 2020 and August, 2021, IPPF/WHR in collaboration with TAHECAP implemented a project on Harm Reduction Services in six regions of Mwanza,Mtwara,Dododma,Lindi,Arusha and Kilimanjaro with objectives to; build the capacity of Harm Reduction Model among Healthcare Providers, access the acceptability and effectiveness of the Harm Reduction model among women seeking abortion services, integrate the Harm Reduction services within existing services and document care providers’ perceptions,Data collection was done using; guided questionnaires and Interviews.

**Results**

780 women seeking abortion services received Harm Reduction Services from 60 health facilities in all 6 regions between ,September 2020 & August, 2021.720 (92%) came for a follow-up visit,690(96%) got Family Planning method on follow-up, Out of 690(96%) who received Family Planning;98 (14%) received implants,300(43%) Depo-Provera, 100 (14%) received IUCD, 192(28%) received COC.For those women who accessed abortion services; Many knew exactly who to see, Some knew the provider personally, few women came blindly.Those who came blindly a significant proportion asked non-care giving staff.A few followed routine procedures and requested for abortion services when entered a consultation room.

Out of 780,602(77%) women terminated their pregnancies,178(23%) didnt terminate their pregnancies after Harm reduction councelling.Out of 602 women who terminated their pregnancies;581/602 (97%) used misoprostol, cost from TZS 20,000-120,000 for a dose of 12 tabs of 200mcg,21(3%) used MVA due to advanced GA .

Eighty percent (80%) reported getting Misoprostol from pharmacies. 99% of women who got Misoprostol said it was easy to get.95% of women said would return if in similar situation.690 (88%) said they would refer a friend.

**Conclusion**

Most women with an unwanted pregnancy had used FP and stopped, economic hardship and having a small child two main reasons for pregnancy termination. We recommend inclusion of Harm Reduction Services in our RCH clinics, for reduction of Maternal Mortality and Morbidity due to unsafe abortion.

## A15 Assessment of knowledge, attitude and practice towards antimicrobial resistance among students in 3 secondary schools in Dodoma

### Erick Venant^1^, Baritazar K Stanley^1^, Michael J Mosha^1^, Dorine Grace J Mushi^1^, Eva Ombaka^2^, Kelvin E Msovela^2^, Pendo Masanja^3^, Karin Wiedenmayer^4^

#### ^1^Roll Back Antimicrobial resistance Initiative, Dodoma, Tanzania; ^2^St John’s University of Tanzania, Dodoma, Tanzania; ^3^University of Dodoma Tanzania, Dodoma, Tanzania; ^4^Swiss Tropical and Public Health Institute, University of Basel, Basel, Switzerland

##### **Correspondence:** Erick Venant (erickvenant@rbainitiative.or.tz)

**Introduction**

Antimicrobial resistance is still not given enough attention and the public is insufficiently aware of its existence, leading to behavior, which propagates the rise of antimicrobial resistance (AMR). One of the objectives of Tanzania's national action plan on antimicrobial resistance is to improve awareness and understanding of antimicrobial use and resistance through effective communication, education and training. This task will need involvement of many stakeholders and sectors.

**Objectives**

The main objective of the study was to assess the knowledge, attitudes and practices toward antimicrobial use and resistance among students in three secondary schools in Dodoma city.

**Methodology**

For this interventional pre-post comparative study, data was collected before and after training on antimicrobial use and resistance. Secondary school students from Mkonze, Merriwa and Kiwanja cha Ndege secondary school who are members of AMR school clubs participated. Training included classroom teaching and arts and crafts. We used quantitative and qualitative data collection methods by using self-administered paper-based structured coded questionnaires delivered to the students with supervision of school guardians. Analysis was done through Excel and SPSS.

**Results**

Three aspects were investigated: awareness of ways to reduce AMR; knowledge that antibiotics cannot be used to treat flu and factors that contribute to AMR. Before the training knowledge of these was below 30%. Three months after the training knowledge had increased to above 90%.

**Conclusion**

Training of secondary school students significantly improved awareness, knowledge and attitude regarding antimicrobial use and antimicrobial resistance. AMR school clubs are an effective vehicle to raise awareness and mitigate the AMR crisis. Focus on students will lead to wider awareness in the community.

## A16 Enhancing community awareness on health insurance and uptake of the improved community health fund (ichf)

### Elizeus Rwezaura^1^, Sia Tesha^1^, Manfred Stoermer^2^

#### ^1^Health Promotion and System Strengthening Project, Dodoma, Tanzania; ^2^Swiss Tropical and Public Health Institute, Basel, Switzerland

##### **Correspondence:** Elizeus Rwezaura (elizeus.rwezaura@hpss.or.tz)

**Introduction**

Since July 2018, the Government of Tanzania adopted and started the implementation of the community health fund (CHF) Iliyoboreshwa as an improved community health insurance scheme mainly targeting population in the informal sector. The improved CHF scheme is operated through the President’s Office – Regional Administration and Local Government (PORALG); accessible in all regions in mainland Tanzania. The governance and operational of improved CHF is embedded in the existing government structures from the national to the village level.

**Objectives**

In December 2020 PORALG in collaboration with HPSS project conducted an annual iCHF performance assessment with objective to assess the implementation structures, implication on achievement of anticipated results and impact.

**Methodology**

The assessment covered all 26 regions in Tanzania and 3 councils from each region resulting in a 34 councils which is 18.4% of total councils. The quantitative data collection method used whereby trained data enumerators interviewed the CHF teams in the respective regions using ODK application. Analysis was done through STATA software.

**Results**

The assessment results indicate that operational and administration structure is well established with defined staffing. On the progress and implementation, the cumulative number of iCHF members from July 2018 has progressively grown to 3,032,007 individuals by the end of July 2021. However, some of these members have their membership expired and as of July 2021 only 1,641,980 households have active policies equivalent to 3.23% of total population in mainland Tanzania. Premiums collected increased also funds disbursed TZS11,810,333,275 to health facilities since July 2018 to June 2021 on services utilized by iCHF members.

**Conclusion**

There is no clear record of the initial investment put in CHF Iliyoboreshwa. It still not clears on whether a matching funds policy exists under this improved CHF scheme; since the rollout of iCHF matching fund was paid once in February 2020; this brings an alert that without external subsidise, financial sustainability will only depend on the extent of enrolment coverage of which to breakeven the coverage should b e at least 30%. The income generated, after provider re-imbursement it will be possible to cover full operational cost and minimal reserves.

## A17 Community engagement in the trial of insecticide-treated durable wall lining for malaria control in rural Tanzania

### Peter E Mangesho^1^, Mohamed S Mohamed^1^, Ruth C Mnzava^1^, Kaseem J Mkuza^1^, George Mtove^1^, William N Kisinza^1^, Joseph P Mugasa^1^, Kihomo R Mpangala^2^,Yara A Halasa-Rappel^2^, Donald S Shepard^2^, Louisa A Messenger^3^, Aggrey R Kihombo^4^

#### ^1^National Institute for Medical Research, Dar es salaam, Tanzania; ^2^Brandeis University, Waltham, United States of America; ^3^London School of Hygiene and Tropical Medicine, London, United Kingdom; ^4^Mzumbe University, Morogoro, Tanzania

##### **Correspondence:** Peter E Mangesho (mangeshop@gmail.com)

**Background**

Community engagement (CE) is gradually promoted for Bio-medical research conducted in resources poor settings. During community trials CE is a complex social phenomenon that defies simple explanation or mechanization employed to engage the community. However, there is scarcity of documented local experiences on CE in Bio-medical research in Tanzania.

**Objective**

To assess the sensitization process, experiences and challenges in improving understanding and subsequent acceptance of an insecticide treated durable wall lining project in rural Tanzania.

**Methodology**

Prior the project we conducted meetings with village leaders to introduce the project. Village leaders prepared community engagement meetings by using the traditional approach “Mbiu”to invite the villagers at the meetings places, whereas, the researchers in support of local leaders addressed the community about the trial.

**Results**

The meetings were poorly attended due long walking distance to the meeting locations, farming activities and presidential election campaigns. Sensitization was re-strategized to add door-to-door sensitization, announcements using a megaphone, designing and distribution of brochures detailing the study objectives and consenting process. The process continued during all three installation phases.

The new strategies rose an acceptance rate from 31.5% to 61.5%. However, some clusters still had some refusals. Reasons included gender and consent, For example, in some houses the head of house (generally a man) refused installation after the wife had accepted; Old rumors resurfaced that ITWL contributed to male impotence. Some installers, initially unprotected, developed skin rashes and the message reached all over. Fear of damaging house walls. Directives that children should not touch the wall liners and confusion from installation delay all fed into refusal rates.

**Conclusions**

Re-strategizing sensitization plus continuous sensitization throughout and after the official installation period increased ITWL acceptance. Future projects should not rely on a single sensitization approach and consider using specialized village research committees for improved CE.

## A18 Youth as agents of change in raising antimicrobial resistance awareness in the community in Dodoma region

### Erick Venant^1^, Baritazar K Stanley^1^, Dorine Grace J Mushi^1^, Michael J Mosha^2^, Eva Ombaka^2^, Israel Mwingira^2^, Karin Wiedenmayer^3^, Roger Harrison^4^

#### ^1^Roll Back Antimicrobial resistance Initiative, Dodoma, Tanzania; ^2^St John’s University of Tanzania, Dodoma, Tanzania; ^3^Swiss Tropical and Public Health Institute, University of Basel, Basel, Switzerland; ^4^University of Manchester, Manchester, United Kingdom

##### **Correspondence:** Erick Venant (erickvenant@rbainitiative.or.tz)

**Introduction**

One of the strategic objectives of the AMR national action plan is to improve public awareness and understanding of AMR. Changing peoples’ behaviour is not easy and the older a person is, the more they are set in their ways. Schoolchildren are in their formative years, which is the right time to impart knowledge and best practices that will guide their behaviour in life. Importantly, students have links with families and communities and are future leaders. Roll Back Antimicrobial resistance Initiative (RBA Initiative) has been pioneering the use of youth as agents of change to increase AMR awareness, promote positive behavioral change and thus reduce treatment failure.

**Objectives**

The main objective was to equip young people (schoolchildren) with knowledge and skills to understand antimicrobial use and resistance and ability to pass the knowledge to their families, other students and community at large.

**Methodology**

Through AMR clubs, RBA Initiative used different methodologies to engage and educate schoolchildren on AMR. These included classroom teaching, arts and crafts like songs, skits, drama, traditional dances and storytelling and fun videos. Further motivation for active participation was encouraged through competition. The content of the training included topics such as behavior that fuels AMR, hand hygiene and sanitation, the impact of fake drugs and the one- health approach.

**Results**

In 2020, the project build the capacity of 160 students. Subsequently, these trained students have reached over 1000 fellow students and over 800 community members including family members with key AMR messages.

**Conclusion**

If Engaged and empowered young people are able to increase community knowledge and awareness regarding AMR as agents of change, contributing to the national action plan on AMR.

## A19 An assessment of knowledge and hand hygiene practices among healthcare workers at Dodoma regional referral hospital

### Erick Venant^1^, Eva A Ombaka^2^, Valence Ndesendo^2^, Karin Wiedenmayer^3^

#### ^1^Roll Back Antimicrobial resistance Initiative, Dodoma, Tanzania; ^2^St John’s University of Tanzania, Dodoma, Tanzania; ^3^Swiss Tropical and Public Health Institute, University of Basel, Basel, Switzerland

##### **Correspondence:** Erick Venant (erickvenant@rbainitiative.or.tz)

**Introduction**

The WHO promoted “My 5 moments for hand hygiene” is designed to prevent the spread of infectious organisms in health-care settings which occurs mostly via contaminated hands of health care workers (HCWs), items/equipment and the environment. Information on its implementation in Tanzania is needed.

**Objectives**

The main objective of the study was to assess knowledge, availability and access to facilities for hand hygiene and the adherence to the WHO’s 5 moments of hand hygiene by healthcare workers at Dodoma Regional Referral Hospital. Bacterial content of cell phones of HCWs was assessed as source of microbial contamination.

**Methods**

This was a descriptive cross-sectional study. Three sets of data were collected through i) questionnaires to assess knowledge, attitudes and barriers to effective hand hygiene, ii) observation to assess adherence of five moments of hand hygiene, and iii) laboratory examination of cell phones for bacterial contamination. Analysis used SPSS.

**Results**

Over 75% of HCWs had formal training on hand hygiene and had access to hand washing facilities, but only 63% were aware of the ‘WHO 5 moments’. Of 270 doctors, nurses and health care students only 7 (2.6%) complied with expected action with no difference between groups. No hand hygiene was observed after touching cell phones. Thirty four percent of sampled cell phones were contaminated with Staphylococci species, which were resistant to penicillin and had varying resistance to Erythromycin, Clindamycin and Gentamycin.

**Conclusion**

Hand hygiene is the most effective way to reduce the risk of health care associated infections. However, many healthcare workers do not adhere to recommended hand hygiene despite training and availability of hand washing facilities. The low practice of hand hygiene means that cell phones of health care workers can easily act as reservoir of transmissible organisms. Use of cell phones as a source of contamination must be included in interventional training of HCW.

## A20 Determination of butyrate ameriolative effects on the severity of dexamethasone-induced diabetic changes in rats

### Rehema S Shungu, Joshua J Malago

#### Sokoine University of Agriculture, Morogoro, Tanzania

##### **Correspondence:** Rehema S Shungu (rshungu08@gmail.com)

**Background**

Diabetes mellitus is a condition that is characterized by abnormal high amounts of glucose levels in the blood. Recent studies have shown a correlation between the microbial compositions in the human gut with occurring metabolic disorders as human gut represents a large contact area between the body and the external environment. A change in the microbiota composition results to an alteration of the gut metabolic activity and microbiota fermentation products such as butyrate resulting to an imbalance.

**Objectives**

The aim of this study was to determine the beneficial effect and protective potency of butyrate on diabetes and study the diabetic model using rat models as human reference and sodium butyrate as source of the butyrate.

**Methodology**

Using complete randomized method, a total of 18 rats aged 6-8months and weighing 90-150g were used. They were acclimatized for 1 week accordingly. Diabetes mellitus was induced by injecting rats with 4 mg/kg dexamethasone intraperitoneally for four consecutive days following or without prior treatment with 100 mg/kg butyrate (intraperitoneally) every other day for seven days in respect to treatments. On day 14, blood glucose levels were determined by glucometer plus device.

**Results**

The group that received butyrate only had the highest mean blood glucose level of 5.45mg/dl followed by dexamethasone only treated group with 5.1mg/dl. An increase in the average body weight was seen in all groups. The rats were then humanely sacrificed by chloroform, autopsied. The liver and pancreas of each group were taken and fixed in 10% neutral buffered formalin for 24 hours, and prepared for histological evaluation of tissue damage under a light microscope under supervision of an experienced pathologist. Hepatic micro and macro vesicular steatosis, pyknosis, acidophilic bodies, vacuolation and sinusoidal ectasia were observed. In the pancreas the numbers of Islets of Langerhans were increased and hyperplastic observed. Reduction of the severity of these changes by butyrate was observed.

**Conclusion**

It is concluded that butyrate suppresses the deleterious effects of dexamethasone-induced diabetic changes. This observation eludes the fact that butyrate supplements may have protective ramifications against diabetes mellitus.

## A21 Epidemiological trend and associated demographic factors for influenza cases in Tanzania, 2016-2019

### Vulstan Shedura^1^, Doreen Kamori^1^, Ally Hussein^1^, Ritha Willilo^2^, Salum Nyanga^3^, Geofrey Mchau^4^

#### ^1^Muhimbili University of Health and Allied Sciences, Dar es salaam, Tanzania; ^2^World Health Organization, Dar es salaam, Tanzania; ^3^National Public Health Laboratory, Dar es salaam, Tanzania; ^4^Ministry Of Health, Dar es salaam, Tanzania

##### **Correspondence:** Vulstan Shedura (vulstanshedura@gmail.com)

**Background**

Influenza is a disease of public health importance that accounts for up to one million of total health related global death annually.

**Objectives**

To determine the epidemiological trend and associated demographic factors for influenza cases in Tanzania from 2016 to 2019.

**Methodology**

A cross-sectional study was conducted using secondary data obtained from national laboratory information system from 2016 to 2019. Logistic regression model was used to check the significant predictors for influenza.

**Results**

A total of 7260 swab samples were collected between 2016 to 2019 from clients with median age of 4 years [IQR=25; 26-1], most samples (53.4%) were from patients aged under five years. Most samples (19%) were collected from Mwananyamala hospital. Overall sample collection was lower in 2016, but increased from 2017 to 2019. Trend shows strong evidence of correlation between SARI and ILI case definitions on influenza positivity (r=0.78[0.60-0.799]). Laboratory confirmation was done by PCR technique whereby cases were 17% with higher prevalence of influenza A [12% (881/7260)] as compared to influenza B [5% (373/7260)]. We observed the seasonality of influenza whereby higher number of cases occurred in rainy and cold seasons (January to June) than the rest of other months. In bivariate and multivariate logistic regression analysis, the factors including age, case definition type and sentinel site were significantly associated with influenza positivity (p<0.05). Patients who presented with SARI were more likely to have influenza as compared to those who had ILI symptoms (aOR 0.75, 95% CI [0.64-0.89], P=0.001). Those who presented with ILI symptoms were more likely to be detected for influenza B as compared to those with SARI.

**Conclusion**

Having known the seasonality of the disease apprises for the proper allocation of resources for the surveillance activities. Since Influenza viruses may have pandemic potential, surveillance activities are inevitable to create epidemiological awareness for prompt public health action.

## A22 Timing and risk factors for early and late death among tuberculosis patients in Tanzania

### Elias M Bukundi^1^, Francis Mhimbira^1^, Candida Moshiro^1^, Rogath Kishimba^2^, Zuweina Kondo^3^

#### ^1^Department of Epidemiology and Biostatistics, Muhimbili University of Health and Allied Sciences, Dar es salaam, Tanzania; ^2^Tanzania Field Epidemiology and Laboratory Training Programme, Dar es salaam, Tanzania; ^3^National Tuberculosis and Leprosy Programme, Ministry of Health, Community Development, Gender, Elderly and Children, Dodoma, Tanzania

##### **Correspondence:** Elias M Bukundi (eliasbukundi@gmail.com)

**Introduction**

Tuberculosis (TB) is the leading infectious disease killer which accounted for more than 1.6 million deaths in 2019 Worldwide. In Tanzania, TB mortality is twice as high compared to global TB mortality. This study aimed to determine the timing of TB death, mortality rate, and risk factors for early and late TB death in Tanzania.

**Methods**

We conducted a retrospective cohort study using National TB program data of all TB patients enrolled on TB treatment from January 2017 through December 2017. We calculated case fatality rate as percentage and mortality rate per 100 person-months (pm) using Kaplan-Meier method. A Cox proportional hazard model was used to identify risk factors associated with early and late TB death. Hazard ratios and their respective 95% confidence intervals for each phase of tuberculosis treatment were reported.

**Results**

More than two-thirds of TB deaths, 2023 (68.7%) occurred in the first two months of TB treatment. Mortality rates were 34.7 and 11.1 per 100 pm in the first and second month of TB treatment respectively. The overall median (interquartile range) survival time for those who died was 39 (36-41) days. A high risk of late TB death was observed among those referred from care and treatment centre (adjusted hazard ratio (aHR) = 1.21, 95% confidence interval (CI) = 1.01- 1.45) and other referral types (aHR = 1.76, 95% CI = 1.34-2.33). Advanced age, TB/HIV co-infection, clinically diagnosed TB patients, and using facility-based DOT option were independent risk factors associated with TB death at any time during TB treatment.

**Conclusion**

We observed a high TB mortality rate and shorter median survival time in the first two months of TB treatment. Prioritizing intervention focused on the first two months of tuberculosis and high-risk groups will reduce more than two-third of tuberculosis-related death.

## A23 Tracking the elimination of dog-mediated rabies from Pemba island, Tanzania: an observational study

### Kennedy Lushasi^1,2,3^, Kirstyn Brunker^2^, Malavika Rajeev^10^, Elaine Ferguson^2^, Rachel Steenson^2^, Laurie Baker^2^, Roman Biek^2^, Joel Changalucha^1^, Sarah Cleaveland^2^, Nicodemus Govella^1^, Daniel T Haydon^2^, ^1,13^Gurdeep Jaswant, Paul Johnson^2^, Rudovick Kazwala^12^, Tiziana Lembo^2^, Msanif Masoud^11^, Mathew Maziku^6^, Eberhard Mbunda^6^, Geofrey Mchau^7^, Francois-Xavier Meslinx^2^, Ally Z Mohamed^4^,Emmanuel Mpolya ^3^, Zacharia Mtema^1^, Chanasa Ngeleja^5^, Kija Ng’abhi^8^, Hesron Nonga^6^, Khasim Omar^4^, Kristyna Rysava^9^, Maganga Sambo^1^, Lwitiko Sikana^1^, Katie Hampson^1,2^

#### ^1^Environmental Health and Ecological Sciences Department, Ifakara Health Institute, Dar es salaam, Tanzania; ^2^Boyd Orr Centre for Population and Ecosystem Health, Institute of Biodiversity, Animal Health and Comparative Medicine, College of Medical, Veterinary and Life Sciences, University of Glasgow, Glasgow, United Kindom; ^3^Global Health and Biomedical Sciences, School of Life Sciences and Bioengineering, Nelson Mandela African Institution of Science and Technology, Arusha, Tanzania; ^4^Department of Livestock development, Pemba, Ministry of Livestock Development and Fisheries, Zanzibar; ^5^Tanzania Veterinary Laboratory Agency, Dar es salaam, Tanzania; ^6^Ministry of Livestock Development and Fisheries, Dodoma, Tanzania; ^7^Department of Epidemiology, Ministry of Health, Community Development, Gender, Elderly and Children Dodoma, Tanzania; ^8^Department of Social Medicine and Public Health Sciences, University of Dar es Salaam, Mbeya, Tanzania; ^9^University of Warwick, Coventry, United Kingdom; ^10^Department of Ecology and Evolutionary Biology, Princeton University, New Jersey, United States of America; ^11^Ministry for Health and Social Welfare, Pemba, Zanzibar; ^12^Department of Veterinary Medicine and Public Health, Sokoine University of Agriculture, Morogoro, Tanzania; ^13^The University of Nairobi, Nairobi, Kenya

##### **Correspondence:** Kennedy Lushasi (klushasi@ihi.or.tz)

**Background**

Rabies has circulated on Pemba Island, off the Tanzanian mainland, since the late 1990s. In 2010, a rabies elimination program began in southeast Tanzania including Pemba. We used contact tracing and whole genome sequencing to investigate the dynamics of rabies elimination from Pemba.

**Methods**

We used contact tracing initiated from routine surveillance of animal bite injuries to identify rabid animals, human rabies exposures and deaths, and whole genome sequencing of viruses for phylogeographic analysis. With government census data and post-vaccination transects we estimated dog vaccination coverage over time. We combined the epidemiological data and inferred phylogenies to construct transmission chains, compute the reproduction number, R, and estimate the impacts of control and prevention measures.

**Findings**

From 2010 until 2014 vaccination coverage increased (from <10% to >50%), whilst animal rabies cases, human rabies exposures and deaths declined. R remained at <1 for two years until the last detected case in May 2014. At least five divergent virus lineages circulated during this period. Following re-emergence in July 2016 emergency dog vaccination took place in September with annual campaigns thereafter. We identified two independent viral introductions from the mainland that seeded two transmission chains, leading to 102 animal cases, 180 exposures and 3 deaths in the ensuing outbreak. Increasing dog vaccination coverage was associated with declining cases and R, until elimination again in 2018.

**Interpretation**

The diversity of genetic lineages show how introductions from mainland East African led to endemic circulation on Pemba in the past and remain a threat to Pemba’s rabies free status. Re-introductions should be expected as part of rabies elimination programmes with dog vaccination coverage and strengthened surveillance needed to detect and reduce their impact. Improved border control may minimize introductions but scaled up dog vaccination on the mainland should have wider benefits in achieving the global ‘zero by 30’ goal to eliminate dog-mediated human rabies deaths.

## A24 Medpack, a mobile app to simplify pharmacy services in Tanzania

### Henry Mathayo^1^, Erick Sollo1^1,2^, Isack Felix^1,3^

#### ^1^Medpack Tanzania Limited, Dar es salaam, Tanzania; ^2^S.Fransis University College of Health And Allied Sciences, Morogoro, Tanzania; ^3^National Institute Of Transportation,Dar es salaam, Tanzania

##### **Correspondence:** Henry Mathayo (henrymathayo49@gmail.com)

**Background**

MEDPACK TANZANIA LIMITED is a registered startup company born in September 2019 in dedication to solve health challenges using digital devices for the improvement of health and wellbeing of Tanzanians and Africa at large. MedPack Tanzania is one of the growing and trusted youth led health startup in Tanzania with well established team of IT and health professionals. MedPack provide online Pharmacy (website and Mobile App) for doctors ,pharmacists, caregivers and Patients to order and buy medication and other pharmaceutical items online from trusted pharmacies and we do quick deliver to door step. Good more on mobile App people can learn different ways on prevention of diseases, proper use of medication. So, we help health care providers, caregivers and patients’ easy access to medication and Access to SRHR Information.

**Objectives**

To simplify access to medication and Pharmacy Items in Health centers and personalized service to individuals in Tanzania particularly Dar es Salaam where we are located right now. Also, to determine the effect of misuse of prescription drugs to youth aged 15 -24 and come up with best solution that fit youth generation.

**Methodology**

To came up with solution MedPack done a survey to 12 hospitals in Dar es Salaam, 10 dispensaries and 5 clinics to determine the problem faced by patients, caregivers and healthcare to access medication from different pharmacies. Also, a survey was done also to youth at National instate of Transport (NIT) and University of Dar es salaam to determine the effect of misuse of prescription of drugs to youth aged from 15-24.

**Result**

During the study we get to know in some hospital , Patients spend 1-6 hours waiting for different medical services at hospitals. After examination you can found patients spend other 30 minutes to 1 hour at the pharmacy waiting for medication. At the pharmacy patient or caregiver can found 43% of prescribed medicines are out of stock in pubic and 35% in private facilities. Here Pharmacist or doctor direct Patients/care givers to go our searching for medication . It can take 1 to 3 community pharmacies to get those prescribed medicines hence wasting more time and money. Survey done to youth in universities at National Institute of Transport (NIT) and university of Dar es Salaam . We found that 55% youth aged from 15 – 24 are exposed to misuse of prescribed medicines. This also supported by Mayo Clinic proceeding article on Prescription drugs show that 47.5% of youth aged 15-24 misuse drugs due to Lack of Knowledge on prescription drug and their potential harm.

**Conclusion**

Our survey came up with conclusion to find a solution that could help health professionals , caregivers and Patients to access Pharmacy services quicker and easy and improve access to Health information among youth . And best way to solve this is the use of technology. MedPack came in to solves above Problems as follow:- MedPack provide online Pharmacy (website and Mobile App) for doctors ,pharmacists, caregivers and Patients to order and buy medication and other pharmaceutical items online from trusted pharmacies and we do quick deliver to door step. Good more on mobile App people can learn different ways on prevention of diseases, proper use of medication. So, we help health care providers, caregivers and patients’ easy access to medication and Access to SRHR Information.

## A25 The patterns and cause-specific in-hospital mortality among older children and adolescents in Tanzania, 2006

### Leonard E Mboera^1^, Susan F Rumisha^2,3^, Veneranda M Bwana^4^

#### ^1^SACIDS Foundation for One Health, Sokoine University of Agriculture, Morogoro, Tanzania; ^2^Malaria Atlas Project, Geospatial Health and Development, National Institute for Medical Research, Dar es Salaam, Tanzania; ^3^Telethon Kids Institute, West Perth, Australia; ^4^Amani Research Centre, National Institute for Medical Research, Tanga, Tanzania

##### **Correspondence:** Leonard E Mboera (lmboera@gmail.com)

**Background**

Despite the available statistics, little is known on the causes, trends and pattern of mortality among older children (5–9 years) and adolescents (10–19 years). This retrospective study was carried out to determine the pattern and cause-specific in-hospital mortality among older children and adolescents in Tanzania.

**Methods**

A multistage sampling technique was employed to select a representative number of hospitals from regions and districts in Tanzania. Analysis of in-hospital mortality from 2006 to 2015 was performed to identify leading cause of deaths, geographical, temporal and demographic variations. Age-standardized hospital mortality rates per 100,000 were calculated. A Bayesian spatial logistic regression model was used to estimate the spatial patterns and probability of dying from leading causes of death.

**Results**

A total of 247,976 deaths were reported from 39 hospitals during the 10-year period. Of these, 17,898 (7.2%; 95% CI: 7.1-7.4) affected the 5-19-year-old individuals. The 5-9, 10-14 and 15-19 years accounted for 42.5% (95% CI: 41.2-43.8), 26.8% (95% CI: 25.6-28.0) and 30.7% (95% CI: 29.5-31.9) of the deaths, respectively. The overall age-standardized mortality rates for 2006-2010 and 2011-2015, were 187.4 and 329.4 deaths per 100,000 population, respectively. The five major specific causes of death in older children and adolescents were malaria (28.8%), anaemia (16.9%), respiratory diseases (5.7%), injury (5.1%), and meningitis (3.9%). The geographical distribution of the probability of dying from the top causes varied by cause of death and geographical region.

**Conclusion**

In Tanzania, mortality among older children and adolescents contribute to about 7.2% of the total hospital deaths. There are significant variations on major causes of death between sex and age as well as geographical regions. These findings call for strategic multisectoral public health responses to reduce deaths among older children and adolescents.

## A26 Community engagement in the trial of insecticide-treated wall lining for malaria control in rural Tanzania

### Peter E Mangesho^1^, William N Kisinza^1^, Mohamed S Mohamed^1^, Joseph P Mugasa^1^, Ruth C Mnzava^1^, Kaseem J Mkuza^1^, George Mtove^1^, Kihomo R Mpangala^2^, Donald S Shepard^2^, Yara A Halasa-Rappel ^2^, Louisa A Messenger^3^, Aggrey R Kihombo^4^

#### ^1^Natioanal Institute for Medical Research, Dar es salaam, Tanzania; ^2^Brandeis University,Waltham, United States of America; ^3^London School of Hygiene and Tropical Medicine, London, United Kingdom; ^4^Mzumbe University, Morogoro, Tanzania

##### **Correspondence:** Peter E Mangesho (mangeshop@gmail.com)

**Background**

Community engagement (CE) during community trials is a complex social phenomenon that defies simple explanation or mechanization. We present findings from an assessment of the sensitization process, experiences, and challenges in improving understanding and subsequent acceptance of an insecticide-treated wall lining (ITWL) program.

**Methodology**

The initial project sensitization plan relied on the traditional approach of invite villagers to meetings with researchers. However, meeting schedules coincided with farming activities and Tanzania’s presidential elections, resulting on poor attendance. Sensitization was re-strategized to add door-to-door sensitization using local advocates, announcements using a megaphone, designing and distribution of brochures detailing the study objectives and consenting process. The process continued during the ITWL installation phase.

**Results**

Following re-strategizing of sensitization the number of ITWL acceptance rose from 31.5% to 65.1%. However, some clusters still had some refusals. Reasons included gender and consent, For example, in some houses the head of house (generally a man) refused installation after the wife had accepted; Old rumors resurfaced that that ITWL contributed to male impotence. Some installers, initially unprotected, developed skin rashes. In one case, one resident’s skin rashes spread fear to a whole hamlet. Households with better socio-economic status cited personal ability to control malaria and feared damage to their walls by the installation process. Directives that children should not touch the wall liners and confusion from installation delay all fed into refusal rates. Rumors of side effects from the ITWL contributed much on project challenges including refusals. Re-strategizing sensitization plus continuous sensitization throughout and after the official installation period increased ITWL acceptance.

**Conclusion**

Future projects should incorporate continuous sensitization and consider using specialized village research committees for improved CE.

## A27 Epidemiological trend and associated demographic factors for influenza cases in Tanzania, 2016-2019

### Vulstan Shedura^1, 2^, Doreen Kamori^2^, Geofrey Mchau^1, 3^, Ritha Willilo^4^, Ally Hussein^1, 5^, Salum Nyanga^6^

#### ^1^Tanzania Field Epidemiologyand Laboratory Training Program, Dar es salaam, Tanzania; ^2^Muhimbili University of Health and Allied Sciences, Dar es salaam, Tanzania; ^3^Ministry of Health in Tanzania, Dar es salaam, Tanzania; ^4^World Health Organization, Dar es salaam, Tanzania; ^5^Centres for Disease Control and Prevention, Dar es salaam , Tanzania; ^6^National Public Health Laboratory, Dar es salaam, Tanzania

##### **Correspondence:** Vulstan Shedura (vulstanshedura@gmail.com)

**Background**

Influenza is the major public health of concern that accounts for up to one million of total health related global death annually.

**Objectives**

To determine epidemiological trend and associated demographic factors for influenza cases in Tanzania from 2016 to 2019.

**Methodology**

The cross-section study design was conducted using secondary data obtained from laboratory information system from 2016 to 2019. Logistic regression model was used to check the significant predictors for influenza.

**Results**

A total of 7260 samples were collected between 2016 to 2019 from clients with median age of 4 years [IQR=25; 26-1], most samples were from patients aged under five years. Most samples (19%) collected from Mwananyamala hospital. Overall sample collection was lower in 2016, but increased from 2017 to 2019. Trend shows strong evidence of correlation between SARI and ILI case definition on influenza positivity (r=0.78[0.60-0.799]). Laboratory confirmed cases was 17% with higher prevalence of influenza A [12% (881/7260)] as compared to influenza B [5% (373/7260)]. We observed the seasonality of influenza where higher number of cases occurred in rainy and cold seasons (January to June) than the rest of other months. When bivariate and multivariate logistic regression was done, the factors of age, case definition type and sentinel site were significantly associated with influenza positivity (p<0.05). Patients who presented with SARI were more likely to have influenza as compared to those who had ILI symptoms (aOR 0.75, 95% CI [0.64-0.89], P=0.001). Those who presented with ILI symptoms were more likely to be detected for influenza B as compared to those with SARI.

**Conclusion and recommendations**

Having known the seasonality of the disease apprise the proper allocation of resources for the surveillance activities. Since Influenza viruses may have pandemic potential, surveillance and data review activities are inevitable to create epidemiological awareness for prompt public health action.

## A28 Mentors mothers! A paradyme shift for community prevention of mother to child transmission of HIV programs

### Neema Makyao^1^, Amani Maro^1^, Amos Nyirenda^1^, Agnes John^2^

#### ^1^Amref Health Africa, Dar es salaam, Tanzania; ^2^Christian Social Services Commission, Dar es salaam, Tanzania

##### **Correspondence:** Neema Makyao (neema.makyao@Amref.org)

**Background**

In the implementation of GF 2020-2023, under PMTCT program more emphasis is required for community PMTCT programs to ensure improvement of HIV services among pregnant and breast-feeding women attending PMTCTs with the aim of reducing HIV transmission to the young ones. The target is to eliminate new HIV infection among HIV exposed infants from 8% in 2018 to below 5% in 2023 and to increase access to ART among HIV infected children from 60% in 2016 to 95% by 2023. The intervention is implemented in a total of 10 regions in 330 Facilities which are non PEPFAR supported sites.

**Methodologies**

A team of 10 regional officers collaborated with RHMTs and CHMTs visited all supported regions to identify non-supported sites. A specific tool was developed to collect data based on positivity rate of a site, HIV testing coverage, early ANC booking, DBS collection and testing as well as turn around time. Issues related to community support such as availability of mentor mothers or CHW, champion fathers and CSOs working with PMTCT sites were also identified. Data was analyzed by on descriptive method and other quantitative data was aggregated in the excel spread sheet.

**Results**

Findings from the situational analysis indicated that most of the health care workers (HCWs) in Non PEPFAR supported facilities were not trained on how to collect DBS and facilities lack trained health care personnel on PMTCT in most cases resulting to people and babies losing life which is also associated with low awareness of the communities including poor male involvement. Few HCW are trained on PMTCT and EID services. Majority are providing PMTCT services through experience, on job coaching and mentorship. Regarding existing community structures, the analysis found that CHWs are not active due to lack of support and incentives. No clear mechanism for tracking the mother-baby pairs. Retention has been noted to be low in Arusha after HEI graduate.

**Conclusion**

Deaths of children associated with HIV/AIDS at lower facilities are preventable but they lack technical skills and equipment’s.-DBS to exposed children is not taken timely some reasons being the HCW incompetence and unavailability of DBS kits thus contributing to poor PMTCT services. -The use of Mother Mentors through community support will facilitate improvement of PMTCT services and hence reduction of new HIV infection.

## A29 Pre-vaccination monocyte-to-lymphocyte ratio as a biomarker for the efficacy of malaria candidate vaccines

### Jane P Nyandele^1^, Ummi Abdul^2^, Ally Olotu^2^, Fatma Issah^2^, Said Jongo^2^, Salim Abdulla^2^, George Warimwe^3^, Jean Pierre Van Geertruyden^4^

#### ^1^Hubert Kairuki Memorial University, Dar es salaam, Tanzania; ^2^Ifakara Health Institute, Morogoro, Tanzania; ^3^Kemri Wellcome Trust, Dar es salaam, Tanzania; ^4^University of Antwerp, Antwerp, Belgium

##### **Correspondence:** Jane P Nyandele (nyandele07@gmail.com)

**Background**

Pre-vaccination monocyte-to-lymphocyte ratio was previously suggested as a marker for the efficacy of RTS,S malaria vaccine in the field. In this study, we investigated the potential of monocyte-to-lymphocyte ratio as a marker for the efficacy of RTS,S malaria vaccine in the field and Whole Sporozoite malaria vaccine (BSPZV) in experimental conditions under a controlled human malaria infection trial (CHMI).

**Methods**

Of the 1705 participants in the phase III RTSS field trial, ML ratio was available for 842 participants, of whom our analyses were restricted. Using primary endpoint of time to first clinical malaria episode, we evaluated the statistical interaction between monocyte-to-lymphocyte ratio and RTS,S malaria vaccine efficacy using Cox regression modeling. Data from phase I and II CHMI trials were appended to evaluate the same effect on BSPZV vaccine efficacy using surrogate endpoints of P. falciparum density, multiplication rates and prepatent period.

**Results**

The unadjusted efficacy of RTS,S malaria vaccine was 54% (95% CI 37% to 66%, P <0.001). Interaction between monocyte-to-lymphocyte ratio and RTS,S efficacy did not reach statistical significance before and after adjusting for covariates (HR 0.90, 95% CI 0.45-1.80; P value = 0.77). Unadjusted efficacy of BSPZV malaria vaccine in the appended dataset was 17.6% (95% CI 10% to 28.5%, P<0.001). Consistent with the observations for RTS,S, we report no association between monocyte-to-lymphocyte ratio and the efficacy of BSPZV malaria vaccine against either the prepatent period (HR= 1.16; 95% CI 0.51 to 2.62, P=0.72), parasite density (Spearman rho=0.004, P=0.97) or multiplication rates (Spearman rho=0.031, P=0.799) endpoints.

**Conclusion**

Monocyte-to-lymphocyte ratio may not be an adequate marker for protection with immunization by malaria vaccines. Further investigations on underlying mechanisms of protection as well as immune correlates of protection would provide a clearer explanation of the differences between those protected in comparison with those not protected by vaccination.

## A30 Approaches towards interoperability of electronic medical record systems

### Neema Mkayula, Mercy Mbise

#### University of Dar Es Salaam, Dar es salaam, Tanzania

##### **Correspondence:** Neema Mkayula (mercymbise@gmail.com)

**Background**

Electronic Medical Record (EMR) systems assist healthcare providers to streamline the flow of patients’ information and the availability of real-time health information that helps to determine the effectiveness of healthcare service delivery. However, these EMR are different among hospitals and sources. As a result, it becomes difficult to share informations of patients because EMR systems cannot communicate with each other.

**Methodology**

The study adopted a qualitative case study approach. The research was carried out by narrative literature review to determine factors which influence interoperability. Semi-structured interviews were conducted with key personnel in ICT departments from both RRH and the MNH with the identified factors of interoperability obtained from the reviewed literature. Qualitative data from the interviews were analysed using the content analysis method.

**Results**

This study, through narrative literature review, found eight factors that influence interoperability in hospital EMR systems and they have been categorised into technical and non-technical factors. For technical factors, the findings indicated EMR interoperability in hospitals can be attributed to the use of standardised data type. Also, for non-technical factors, the influence of political supremacy, availability of resources and legal aspects which includes policy and regulations can probably be accounted for the interoperability of EMR systems.

**Conclusion**

Based on these findings, the study recommends approaches towards achieving interoperability in EMR systems which should include; the investment in interoperability technologies to support communication of EMR systems and initiatives to interoperability should start from the Ministry responsible for health. Lastly, there should be a way to manage and oversee if policies and regulations which support EMR interoperability are being followed.

## A31 Improving diagnosis of childhood TB: preliminary results on fujilam and spk from “rapaed-TB”

### Alfred Mfinanga^1^, Harrieth Mwambola^1^, Issa Sabi^1^, Nyanda Ntinginya^1^, Laura Olbrich^2^, Heather Zar^3^, Khosa Celso^4^, Denise Floripes^4^, Marriott Nliwasa^5^, Elizabeth Corbett^5,6^, Robina Semphere^5^, Joy Sarojini Michael^7^, valsan Verghes^7^, Stephen Graham^8^, Rinn Song^9^, Pamela Nabeta^10^, Andre Trollip^10^, Christof Geldmacher^2,10^, Michael Hoelscher^2,10^, Norbert Heinrich^2,10^

#### ^1^Mbeya Medical Research Centre, National Institute for Medical Research, Mbeya, Tanzania; ^2^Division of Infectious Diseases and Tropical Medicine, University Hospital, Ludwig Maximilian University, Munich, Germany; ^3^Department of Paediatrics and Child Health, University of Cape Town, Cape Town, South Africa; ^4^Instituto Nacional de Saúde, Marracuene, Mozambique; ^5^TB/HIV Research Group, College of Medicine, Blantyre, Malawi; ^6^TB Centre, London School of Hygiene and Tropical Medicine, London, United Kingdom; ^7^Department of Clinical Microbiology, Christian Medical College (CMC), Vellore, India; ^8^Centre for International Child Health, University of Melbourne, Melbourne, Australia; ^9^German Centre for Infection Research, Partner Site Munich, Munich, Germany; ^10^Foundation for Innovative New Diagnostics, Geneva, Switzerland

##### **Correspondence:** Alfred Mfinanga (amfinanga@nimr-mmrc.org)

**Background**

The diagnosis of tuberculosis (TB) in children remains challenging: current detection methods neither perform reliably nor are sampling methods child-friendly.

**Methods**

RaPaed-TB is a diagnostic validation study currently conducted in South Africa, Mozambique, Malawi, Tanzania, and India. Enrolment of children ≤14years was initiated in January 2019. Clinical and laboratory workup is standardized across sites, and diagnostic classification follows the current NIH-consensus statement. New tests conducted on site include the urine-based lateral-flow assay Fuji SILVAMP-TB LAM (FujiLAM) and Stool Processing Kit (SPK) for MTB-DNA detection.

**Results**

As of April 2021, 846 participants were enrolled. The median age was 5.7years (1.8-8.8years) with 14% of children being <1year (117/846) and 37% <5years (312/846).Overall Microbiological confirmation rate (PCR/culture) was 21% (182/846).For AlereLAM, the overall sensitivity was 14.5% (95%CI 9.6-20.6) and specificity 92.9% (95%CI 88.0-96.3),while the sensitivity for FujiLAM was 34.9% (95%CI 27.8-42.6) and specificity 87.8% (95%CI 81.8-92.4).In our cohort, the SPK had a sensitivity of 35.8% (95%CI 28.5-43.6) and specificity of 87.9% (95%CI 81.9-92.4).When applying FujiLAM and SPK jointly, the sensitivity was 52.8% (95%CI 44.8-60.7) and specificity 73.6% (95%CI 65.8-80.5).The number of HIV-infected children with confirmed TB was small (16/182); no substantial difference in test performance was seen in this group. Further analysis of collected data is ongoing and therefore updated results will be presented during the forum.

**Conclusion**

The RaPaed-TB cohort allows large-scale evaluation of new tests. To our knowledge this is one of the first studies to prospectively evaluate FujiLAM and SPK head-to-head. Presented data on tests using easy-to-obtain samples indicate a modest performance of FujiLAM and SPK while showing better performance in some age subgroups. Additionally, FujiLAM performed better than AlereLAM with both the new tests indicating an improved performance in the very young and malnourished, showing their potential to aid diagnosis in these particularly vulnerable groups.

## A32 Evaluating two new oxazolidinones for tuberculosis treatment in the panacea-sudocu and -decode trials

### Christina Manyama, Nyanda Elias, Lilian T Minja

#### Mbeya Medical Research Centre, National Institute for Medical Research, Mbeya, Tanzania

##### **Correspondence:** Christina Manyama (cmanyama@nimr-mmrc.org)

**Background**

Tuberculosis (TB) is one of the main causes of morbidity and mortality worldwide, and increasing drug resistance requires new treatment options.

Linezolid, an oxazolidinone, is an effective drug recommended for resistant TB, but a high risk of hematotoxicity and neurotoxicity limits its use. Two novel oxazolidinones, expected to have better safety profiles, are under development: sutezolid (Sequella) and delpazolid (LegoChem Biosciences).

Dose-finding for oxazolidinones for TB cannot be done in the 14-day monotherapy early bactericidal activity study design, since the main toxicities occur only after 14 days of treatment. Instead, a trial design needs to be chosen that permits longer exposure to oxazolidinones through combination therapy with established anti-tuberculosis drugs.

**Objective**

PanACEA have designed two trials with the aim to establish the exposure-response and exposure-toxicity relationship of sutezolid (SUDOCU), funded by EDCTP, and delpazolid (DECODE), sponsored by LegoChem Biosciences.

**Methods**

In each study, 75 adult patients in South Africa and Tanzania with newly diagnosed, drug sensitive pulmonary TB will be openly randomized to five experimental arms to receive bedaquiline–delamanid-moxifloxacin with different doses of sutezolid/delpazolid.

Treatment duration in SUDOCU will be 12 weeks, followed by 3 months standard of care treatment. DECODE patients with good clinical and microbiological treatment response (SCC) will end treatment after 16 weeks, followed by a 36 week observation period to rule out relapse.

Exposure-response and exposure-toxicity modeling will be used to enhance the precision of dose-finding.

**Results**

At time of submission, SUDOCU has started with two participants enrolled in South Africa. DECODE is planned to start soon. We expect to have preliminary data on safety and efficacy to report during the 10th EDCTP Forum in October 2021.

**Conclusion**

The study findings are expected to contribute to the development of new drug regimens against TB, which are safer and shorter to increase compliance with treatment.

## A33 Measuring quality of care practices of underfive children using a formulated ‘care score index'

### John M. Msuya^1^, Elieth D Rumanyika^1,2^

#### ^1^Sokoine University of Agriculture, Morogoro, Tanzania; ^2^Ministry of Health Community Development Gender Elders and Children, Dodoma, Tanzania

##### **Correspondence:** John M. Msuya (jmsuya@sua.ac.tz)

**Background**

While care practices are critically important in determining nutrition status of a child, a common tool for assessing them has not yet been developed. This study attempted to develop a Care Score Index to use as a tool for assessing the quality of care provided to children underfive years of age. The move by this study was an attempt to formulate a more universal tool to be used for assessing child care practices in the Tanzanian context.

**Procedure**

The study consisted of two phases: First was development of a care assessment tool (Care Score Index); and secondly, assessing the quality of care practices in the study area (Temeke Municipality in Dar es Salaam) using the developed tool. The tool comprised of five parameters of child care practices, namely: maternal health seeking, child immunization, child feeding, psychosocial care and hygiene. Literature review and expert opinions were the main methods of developing the tool. On the other hand, a total of 198 mothers with their underfive children were randomly selected to participate in a cross-section study in four wards of Temeke Municipality in Dar es Salaam. Data on the five parameters of care practices were collected through face-to-face interviews and observation of the sampled women. Children’s anthropometric measurements for assessing nutrition status of the study children were also taken. SPSS and WHO-Anthro computer software were used to analyse the collected data.

**Results**

Overall, three quarters of the respondents (75.8%) were categorised as having good care practices, however there were variations in each parameter whereby maternal health seeking care practices scored the lowest (30.3%). Maternal factors such as education, marital status, occupation and number of children were found to have no significant relationship with the care quality scores. The overall care score was significantly associated with weight-for-age z-scores (underweight) at p=0.003 while child feeding practices score was significantly associated with both underweight (p=0.009) and wasting (p=0.017).

**Conclusion**

It is concluded that Care Score Index has great potential in community screening for risks of nutrition vulnerability in developing countries.

## A34 Innovative approach for healthcare quality improvement self monitoring and supportive supervision

### Piensia Nguruwe^1^, Peter Risha^1^, Liberatha Shija^1^, Mwanaali H Ali^2^

#### ^1^PharmAccess Foundation, Dar es salaam; ^2^Ministry of Health, Pemba, Zanzibar

##### **Correspondence:** Piensia Nguruwe (p.nguruwe@pharmaccess.or.tz)

**Introduction**

Digitizing quality improvement process for healthcare providers is essential to, incentivize them and actively engage to support and implement the improvement plan. Pharmaccesss through SafeCare product developed a mobile phone-based quality platform application. The platform is interactive, real time quality management and monitoring designed for health care providers to access their Quality Improvement Plan on line and record implementation progress. The platform increases transparency and benchmarking on quality.

**Methods**

Access to Quality Platform is given after a rating assessment is completed. A rating assessment is conducted and soon after submitting data a quality platform access code is generated. Providers are trained on the platform and the facility admin is connected to quality platform using email. Instructions on next steps are provided if the facility meet requirements for providers’ access. The facility begins using quality platform for provider and data is sent to quality platform for stakeholders. In order to have access to quality platform, the facility should have reliable internet connection, access to smart phone, tablet or computer. And the facility admin/person in charge of Quality Platform must be comfortable with digital tools.

**Results**

59 facilities in Zanzibar are on-board which is equal to 69 % of all facilities that have undergone a SafeCare rating assessment. 15% (9/59) of the facilities visited the App per month and at least one library document is downloaded. During Jan-July 2021 a total of 106 tasks have achieved in all facilities implementing quality platform. Interviewed staff are happy and appreciate to work with digital tool rather than paper work.

**Conclusion**

Digitalizing quality improvement helps facilities to monitor improvements and gather data at appropriate time. The platform enables management to get almost real time data without physical visit to the healthcare facility.

## A35 Routine TB testing surveillance system evaluation at CTRL 2021

### Eustadius K Felician^1^, Said Aboud^1^, Loveness Urio^2^

#### ^1^Muhimbili University of Health and Allied Sciences, Dar es salaam, Tanzania; ^2^Tanzania Field Epidemiology and Laboratory Training Program, Dar es salaam, Tanzania

##### **Correspondence:** Eustadius K Felician (ellykamu@gmail.com)

**Introduction**

Tuberculosis (TB) is a communicable disease that remains a major global health problem and one of the top 10 causes of death worldwide and the leading cause of death from a single infectious agent ranking above HIV/AIDS. Globally, an estimated 10.0 million (range, 8.9–11.0 million) people fell ill with the disease in with the death of 1.2 million in 2019. In Tanzania, TB incidence has fallen from 306 per 100,000 population in 2015 to 253 per 100,000 in 2018. Surveillance system evaluation is very important for funders and stakeholders to know if the system meets its intended objectives. The aim was to assess the performance of the surveillance system for routine TB testing at Central TB Reference Laboratory (CTRL) whether it is meeting its set objectives, assess its usefulness and describe its attributes.

**Methods**

The TB surveillance system was evaluated using Centre for Disease Control and Prevention updated guidelines for evaluating public health surveillance systems 2006. Records and TB database were reviews from 2017 to 2020 to assess objectives of the system and attributes of the system at Central Tuberculosis Reference Laboratory (CTRL), Bugando Medical Centre Laboratory (BMCL), Misungwi and Magu District Hospital. Interviews were conducted at the various levels using a semi-structured questionnaire and data analysis done with Epi info 7 and Microsoft Excel to run frequencies and percentages.

**Results**

The surveillance system is well structured with standardized data collection tools. The system was found to be useful and met its objectives. It was also found to be simple, flexible, and fairly stable with average timeliness in TAT but partially in transit time. It had low acceptability and low geographically representative. It had low sensitivity of 6.8% at BMCL and a moderate CTRL of 66.17%. Predictive value positive was low for both CTRL and BMCL with 23.43% and 19.12% respectively.

**Conclusion**

The surveillance system was found to be useful and met its objectives. There is a need to improve the sensitivity, predictive value positive, timeliness (transit time), representativeness, and acceptability.

## A36 Impact of single nested RCH health provider at a facility level on performance and sustainable FP services

### Esther Lubambi^1^, Leah Anku^1^, Wema Moyo^1^, Rita Mbeba^1^, Zawadi Athanas^1^, Geofrey Sigalla^1,^ Emily Smith^2^

#### ^1^Marie Stopes Tanzania, Dar es salaam, Tanzania; ^2^Marie Stopes Reproductive choices, Dar es salaam, Tanzania

##### **Correspondence:** Esther Lubambi (elubambi@mst.or.tz, lanku@mst.or.tz)

**Background**

Tanzaniais alow-income country with shortage of human resource for health across many prongs of service delivery. Geographicalimbalances cause variations in this shortage with huge disparitiesbetween rural and urban.Toovercome this critical shortage,MSTimplementsa novel “Embedded nurse” (EN) modelas part of its commitment to public sector strengthening (PSS).EN is MST’s provider nested at a health facility to support service provision and capacity building.

**Objective:**

This case study evaluates the effectiveness of the Embedded nurse model in delivering its objectives of service delivery and building capacity of Local government providers (LGA)s.

**Methodology:**

Descriptive and trend analysis was done using data from MST’s routine systemand(DHIS2) in the period of January-June 2020/2021.A total of 27 ENs were included in the analysis,135 facilities visited in a hub and spoke model.

**Results:**

The average number of clients receiving LARCs per month increased from 6,524 before ENs were nested to 9,225, a 41% increase after ENs were nested. Of all LARCs provided, MST ENs contributed about 72%.ENs started to mentor LGA provider in October 2020, to date 105 LGAs provider have been mentored by ENs.

**Conclusions:**

Nesting EN at a health facility proved to yield positive gain in increasing number of users accessing family planning services particularly LARCs.Rotating between hubs and spokes maximize the impact of the EN across many health facilities. Distance proximities need to be considered when designing.Effective capacity building of LGA providers dependson factors including availability of mentees, time allocated for both LGA and the nested provider for training.

## A37 Improving access to reproductive and maternal health services in underserved districts of Simiyu region

### Serafina Mkuwa, Jerome Mackay, Frida Ngalesoni, Aisa Muya, Florence Temu

#### Amref Health Africa in Tanzania, Dar es salaam, Tanzania

##### **Correspondence:** Serafina Mkuwa (serafina.mkuwa@amref.org)

**Background**

Despite global efforts to reduce maternal mortality and morbidity, the burden is high in low and middle income countries including Tanzania, where major causes of deaths are hemorrhage, infection, high blood pressure, unsafe abortion, and obstructed labor. In Tanzania maternal mortality rate (MMR) have actually increased from 454 per 100,000 live births in 2021 to 556 per 100,000 live births in 2016. A major driver of the high rates of death and disability among pregnant women and newborns is the fragmented continuum of care with low coverage of Emergency Obstetric Neonatal Care (EmOC) especially in underserved areas as well as limited information on availability and use of EmOC services.

**Approaches**

Amref Tanzania implemented a four years Uzazi Uzima project that aimed to improve indicators of reproductive, maternal, neonatal, child and adolescents’ health in six councils of Simiyu region, with poor health indicators especially high maternal and neonatal mortality rates. The project focused on improving delivery of quality maternal & newborn health services and increased utilization of maternal & newborn health services by women & their families in the six district councils of the region.

**Methodology**

The ETE study adopted a before-after evaluation cross -sectional design employing both qualitative and quantitative approaches. A sample of 2025 households, 2020 women and 857 men were involved. In addition, 12 FGDs and 12 In-depth Interviews (IDIs) were conducted.

**Results**

Key results compared important indicators with the baseline and showed that the four ANC visits increased from 48.5% in the baseline to 62.3% among women aged 15-49. On partner escort, 83.3% of women aged 15-49 were accompanied by their partners to ANC clinic visit an increase from 75% at baseline. Health facility delivery, increased by 23.5% from 56.30% to 79.8%. Women aged 15-49 attended by a skilled attendant increased from 46.40% to 61.5%. It was reported that nowadays men's involvement in accompanying their partners to the ANC has increased but not during PNC. Majority of the pregnant women make joint decisions with their partners on ANC visits, a few pregnant women need to seek permission before making clinic visits. Women still need to ask for permission from their husbands/partners to use family planning.

**Conclusion**

Hence, the project was a success, but more need to be done

## A38 Factors influencing alcohol consumption among students: a case study of Tusiime high school in Ilala

### Hasitu K Chakachaka, Elizabeth Popova

#### St Joseph University College of Health and Allied Sciences, St Joseph Universy in Tanzania, Dar es salaam, Tanzania

##### **Correspondence:** Hasitu K Chakachaka (Hasitu014@gmail.com)

**Background**

Alcohol use among students is still public health problem. Studies show that the trend has been increasing worldwide since the 90s. Many authors suggesting that earlier initiation of alcohol use being associated with the development of alcohol dependence during adult life. In addition, harmful alcohol use has been linked with acute and chronic adverse health outcomes, as well as negative family, social and behavioral outcomes including risk sexual behaviors.

This study aimed to explore the factors influencing alcohol consumption among students aged 18 years old and above at Tusiime high school in Ilala Municipality, Dar es salaam.

**Methods**

A school-based descriptive cross-sectional study of quantitative approach. Random and systematic sampling techniques were used to sample 153 students aged 18 years old and above from Tusiime high school in ilala municipal. A structured questionnaire used to collect data from respondents. IBM SPSS Statistics 25 use to perform analysis.

**Results**

Spirit was the most consumed alcoholic beverage (66.67%), alcohol use start at 13 years (11.10%). Experimentation (40.28%) was the major reason for initiation of alcohol use. Majority of students who use alcohol have friends who were drinkers (94.34%). Socio-demographic characteristics; age (p=0.03), gender (p=0.00), religion (p= 0.04), parental supervision, peer pressure appears to be significantly associated with alcohol. Moreover, the likelihood of alcohol consumption tends be more in male gender, older age, living with single parent, peer pressure, lack of parental supervision.

**Conclusion**

There is the need to strengthen an early intervention against alcohol use among students like inclusion of substance use topic in school curriculum.

## A39 Factors contributing to using traditional medicine over modern medicine among adults

### Rehema Makorere

#### St Joseph University College of Health and Allied Sciences, St Joseph Universy in Tanzania, Dar es salaam, Tanzania

##### **Correspondence:** Rehema Makorere (rehemamakorere@yahoo.com)

**Background**

Today the world is facing major crisis with energy, food and population. It is none other than plants which can provide the whole thing to conquer all these. Close to these plants utilized as feed, strands, development materials or more all drugs. Plants as medications are utilized in all human progress and culture. Particularly in agricultural nations, the native methods of treatment are important for their way of life, socially worthy and monetarily reasonable. It is assessed that one of every seven of all current plant has restorative or healing force, however just if painstakingly picked and appropriately.

**Aim**

To assess the factors contributing to using traditional medicine over modern medicine among adults at Makuburi ward, Ubungo district, Dar es Salaam region, Tanzania.

**Methods**

A descriptive cross-sectional study was performed at Makuburi ward, Ubungo district, Dar es Salaam region and simple random sampling approach was applied to the sample size of one hundred people. Their knowledge, attitude, practice and factors on the usage of traditional and modern medicine were evaluated with the assist of a structured questionnaire.

**Results**

About 59% were males. The mean age of the study subjects was found to be 31.53 years. About 77% had knowledge about traditional medicine 42% had positive attitude towards traditional medicine.60% have been practicing traditional medicine and the prevalence of traditional medicine is 65%.

**Conclusion**

The study has shown that there is a good knowledge about traditional medicine and modern medicine, and there is also a negative attitude towards traditional medicine. Majority of the residents have been practicing traditional medicine and were aware of the side effects of both medicines and recommending that both medicines being used since they both are important and some of modern medicine comes from traditional medicine.

## A40 Parasite infectivity rates and blood meal sources of host seeking malaria vectors of northeastern Tanzania

### Subam Kathet^1^, Ayman Khattab^1^, Mwantumu Ally Fereji^1, 2^, Gladness Kiyaya^1,2^, Victor Mwingira^1,2^, William Kisinza^1,2^

#### ^1^Translational Immunology Research Program Unit, University of Helsinki, Helsinki, Finland; ^2^Amani Research Centre, National Institute for Medical Research, Tanga, Tanzania

##### **Correspondence:** Subam Kathet (subam.kathet@helsinki.fi)

**Background**

This study was conducted in Muheza district of North-East Tanzania as a part of baseline data collection accounting for a prospective cluster randomised trial to evaluate the efficacy of combining novel vector control tool with LLIN’s in reducing malaria vectors.

**Methods**

Adult mosquito samples were collected for 28 consecutive nights from 20 villages during May-June 2019 and November-December 2019 using a CDC miniature light traps installed in 5 different household from each village randomly selected every night. Collected samples were identified to species using morphological keys and their gonotrophic status were recorded. Engorged specimens were transferred separately for laboratory analysis followed by DNA extraction using CTAB method. Sibling species and blood meal sources were established using PCR and Plasmodium infection was validated using melt curve RT-PCR assay.

**Results**

A total of 1174 blood fed mosquito sample were collected during the long and short rainfall of 2019 of which 72.23% (n = 848) and 27.76% (n = 326) were identified morphologically as Anopheles funestus s.l. and An. gambiae s.l. respectively. Within An. funestus s.l. population, 93.75% (n = 796) were An. funestus s.s., 4.54% (n = 38) were An. leesoni and 1.70% (n = 14) were An. rivulorum while An. gambiae s.l. population comprised of 87.65% (n = 286) An. gambiae s.s. and 12.35% (n = 40) An. arabensis. From melt curve analysis, Plasmodium falciparum infections rates were reported to be 1.21% and 0.58% among blood-fed An. gambiae s.l. and Anopheles funestus s.l. respectively. Based on PCR analysis of vertebrate cytochrome b, humans (56.7 %) were the prominent blood-meal hosts of malaria vectors while 37.33% of blood-meals were from non-human vertebrate hosts.

**Conclusion**

Blood fed mosquito population was dominated by An. funestus s.l. however P. falciparum infection rate was significantly higher in An. gambiae s.l.

## A41 Diagnostic accuracy of xpert MTB/RIF ultra for the diagnosis of pulmonary TB in children

### Issa Sabi^1, 2^, Emanuel Sichone^2^, Hellen Mahiga^2^, Fred Njeleka^2^, Bariki Mtafya^2^, Willyhelmina Olomi^2^, Nyanda E Ntinginya^2^, Michael Hoelscher^1, 3^, Norbert Heinrich^1, 3^, Beate Kampmann^4^

#### ^1^Center for International Health University Hospital, Ludwig Maximilian University, Munich, Germany; ^2^National Institute for Medical Research, Mbeya Medical Research Center, Mbeya, Tanzania; ^3^German Centre for Infection Research, Munich, Germany; ^4^Medical Research Council Unit The Gambia at the London School of Hygiene and Tropical Medicine, Fajara, Gambia

##### **Correspondence:** Issa Sabi (isabi@nimr-mmrc.org)

**Background**

Xpert MTB/RIF Ultra Assay (Ultra) was most recently recommended by WHO for the diagnosis of pulmonary TB (PTB) in children. We evaluated the diagnostic accuracy of Ultra for the detection of PTB using fresh sputum samples in children.

**Methods**

In the recently completed study of the Reach4Kids Africa team, children aged less than 15 years old were considered for enrollment at the Mbeya regional Referral hospital or referred from the nearby health facilities in Mbeya, Tanzania. Children with signs and symptoms suggestive of active TB disease were assessed using history, physical examination, and chest X-rays. Two sputum samples were obtained using sputum induction or by spontaneous expectoration in older children. Sputum samples were analyzed using smear microscopy, Ultra, and liquid culture using BD BACTEC MGIT 960. The diagnostic sensitivity, specificity, and predictive values of Ultra were calculated using culture as the reference standard.

**Results**

Between September 2017 to October 2018, 189 children with presumed TB were enrolled. The median age was 3.4 years, 103 (54.5%) were males and 21 (11.1%) were HIV infected. Bacteriological confirmation (positive culture or Ultra) was achieved in 25 (13.2%) children. Among all children smear detected 8 (4.2%), Ultra detected 19 (10.1%) and culture detected 21 (11.1%) TB cases. The sensitivity of Ultra was 69.0% (95% CI, 19.0 - 97.0), and the specificity was 96.0% (95% CI, 79-100) when assessed against culture as the reference standard. Ultra detected extra 2 cases (1 “low”; 1 “trace”) in the group of children with culture-negative results. Updated results will be presented.

**Conclusion**

Compared with culture as the gold standard, Ultra demonstrated a modest but improved sensitivity for the detection of PTB in children. The addition of a second sample did not increase its sensitivity.

## A42 The power of blended cascading training approach in strengthening provision of essential health service in Tanzania

### Anna-Grace Katembo, Rita Mutayoba, Frida Ngalesoni, Aisa Muya, Florence Temu

#### Amref Health Africa, Dar es salaam, Tanzania

##### **Correspondence:** Anna-Grace Katembo (Annagrace.Katembo@amref.org)

**Background**

Disease outbreaks disrupts provision of essential health service; i.e Ebola outbreak in West Africa showed negative effects on provision of essential services especially linked with HCWs insufficient IPC practices. Data from HIMS in Sierra Leone showed a decrease in maternal and new-born care that contributed to an estimated 3,600 maternal deaths, neonatal deaths and stillbirths. With COVID 19 outbreak, study by John Hopkins University projects a reduction in the MNCH services in low- and middle-income countries (LMICs) leading to an increase in maternal and child deaths. Tanzania will not be immune with virulent waves of COVID-19 calling for strategies to maintain essential health services. Through the UNICEF funding, Amref Tanzania in collaboration with MOHCDGEC and PORALG is responding to WHO guidance on maintaining essential health services (EHS) by rapidly optimize health workforce capacity through IPC trainings.

**Approaches**

Continued Essential Services (CES) projects introduced in August 2021 deployed a blended cascading mode to train health care workers on IPC to ensure that there is continuity of essential health services even during pandemic.

**Results**

The project physically trained 40 national level master trainers who then trained virtually 237 regional and district level trainers. These then trained virtually and physically 1,172 facility based trainers of trainees/facility champions who finally trained their fellow health care workers in their facilities. These facilities champions are now providing ongoing mentorship to their fellow health care workers physically and to date a total of 5,172 health care workers both medical and non-medical have been reached from 297 health facilities in mainland and Zanzibar. With these initiatives essential services as indicated by key indicators for instance institutional deliveries and vaccination has been maintained between August 2020 and June 2021

**Conclusion**

Maintaining EHS should be done holistically. This projects support of one strategy in capacity strengthening of HCWs on IPC should be complemented with other strategies including supplies of medical equipment, WASH, increasing numbers of HRH and improving infrastructure of the projects plan to support “Establish safe and effective patient flow at all levels” THE TRIAGE RENOVATION then call for unified support.

## A43 Women empowerment in sanitation entrepreneurship improves health and livelihood of urban poor

### Mturi James, Elias Kazingoma

#### Amref Health Africa in Tanzania, Dar es salaam, Tanzania

##### **Correspondence:** Mturi James (Mturi.James@Amref.org)

**Background**

Globally, women are disproportionately impacted by poor WASH services due to both social norms and biological factors. Technical solutions alone to WASH problems, don’t address decision-making power or control over resources. Lack of women economic independence compromises women empowerment and perpetuates gender inequality and poverty. Through the funding from Madrid City Council - Spain, Amref Tanzania implemented pro-poor sanitation project that aimed at providing opportunities to women to carry out sanitation economic activities and created opportunities for paid work through selling sanitation products (eg. briquettes, etc)

**Approaches**

Several strategies were used (i) demand creation through Community Health Workers (CHWs) for improved sanitation and hygiene facilities at household level, focusing on waste separation and collection. This followed by (ii) sanitation products development using participatory and bottom-up approach whereby various sanitation and hygiene products were fabricated/recycled from wastes. Women capacitated on Sanitation Business promotion through marketing skills and effective linkages/partnerships. For sustainability purposes, women also empowered to venture into sanitation businesses through financial literacy and provision of loans through revolving funds for those not yet bankable. The project supported women groups with an initial capital of four motor tricycles with trailers; protection gears; safety equipment and with recycling machineries.

**Results**

A total of 154 women from 4-sanitation business groups directly supported and benefited through the project. This has resulted in generating a margin profit of about $10,903 per month equivalent to $70.8 per women group per month. Additionally, a total of 119,805 people have also benefited from women entrepreneurships and skills development, hence raised voices on matters related to sanitation and health development.

**Conclusion**

Women economic empowerment through sanitation business perpetuates gender equality, community health improvement and economic development. WASH programmes should keep on providing women with support needed to carry out social activities, hence significantly contribution to sustainable development.

## A44 Implementation of the new WHO labor care guide: experience from Tosamaganga hospital

### Valentina Locati^1^, Michele D'Alessandro^1^, Donald Maziku^2^

#### ^1^Doctors with Africa CUAMM, Dar es salaam, Tanzania; ^2^Tosamaganga District Designated Hospital, Doctors with Africa CUAMM, Iringa, Tanzania

##### **Correspondence:** Valentina Locati (m.dalessandro@cuamm.org)

**Introduction**

The aim of this pilot study was to implement the new WHO obstetrical management guideline, the Labour Care Guide partograph (LCG) published in 2021 and to compare it with the traditional Modified WHO partograph(MWP). WHO reviewed and revised the design of the previous partograph: the recommendations present in the LCG are evidence-based practices that should be implemented throughout labour. An additional section on respectful maternity care, a revised definition of latent and active stages, duration and progression of the first stage have been implemented. The tool aims to stimulate shared decision making and to promote women-centred care.

**Study design**

A prospective study was conducted among women undergoing delivery at Tosamaganga Hospital, Iringa District Council, during the month of September 2021 (01 september up to 22 september 2021).

**Methods**

The gynaecologist in charge of the maternity ward trained the staff on the use of the LCG, to ensure proper and consistent utilization. Midwifes working in labour room started following labours with LCG in August 2021, in order to highlight problems in the obstetrical management due to the implementation of the new tool. At the end of August, a second audit was conducted with the maternity staff in order to share and solve the challenges reported by the midwifes. During the month of September 2021, women in labour were followed with both partographs, randomly assigned to each woman. Exclusion criteria have been all elective cesarean sections (CS). All women with two previous deliveries by CS, one previous CS with post-delivery sepsis, one previous scar and suspected big baby, previous scar for cephalopelvic disproportion have been excluded from the study.

**Results**

A total of 115 patients, who delivered at Tosamaganga Hospital between the 1^st^ and the 22^nd^ of September 2021. The average age of the population was 29. Variables included aspects such as pregnancy complications, maternal and fetal wellbeing assessment, medication and labour progress. These factors were analysed in order to compare the outcomes in the new WHO Labour Care Guide (LCG) and the old MWP. Labour was spontaneous in 101 out of 115 patients (87,8%), while it was induced in 14 patients (12,2%).

**Discussion**

The average age of this population is 29 years old. The percentage of pre-term babies (9,7%), which is a pregnancy risk factor, is in line with the Tanzanian national rates (approximately 11%). The parity of the women in this study is equally distributed between nulliparous and multiparous. The median number of children for multiparous women in this study is 3. In 2020 fertility rate of Tanzania was 4,8 births per woman.

The main limit of this study is an unsuccessful adoption of the new LCG: as a matter of fact, the amount of data of patients whose labour had been followed with the new LCG was lower than those followed with the MWP. The reason for this could be found in the lack of confidence while using the new tool and poor encouragement of the end-users, which led the maternity staff to choose the old partograph instead of the new one.

## A45 Breaking silence in sanitation marketing; greatly improves health and livelihood

### Mturi James, Elias Kazingoma, Anastazia Rutatina

#### Amref Health Africa in Tanzania, Dar es salaam, Tanzania

##### **Correspondence:** Mturi James (Mturi.James@Amref.org)

**Background**

Globally, over 55% of the population lack access to safely managed sanitation services. Despite declared as lower middle-income country, 75% households in Tanzania lack access to safely managed sanitation facilities (open defecation stands at 7%). The problems increase along with population growth to 58.01million. Financial Inclusion Improves Sanitation and Health (FINISH) Tanzania project has been an evident case indicating that development of the market is the only sustainable approach to meeting the need for sanitation and realization of Tanzania development vision (2030) and the national health goals.

**Approaches**

The FINISH approach premised on Diamond Model that engages both the public and private sectors to improve sanitation and hygiene (S&H) in target communities. The model creates an inclusive local S&H market through cooperation between local government and implementing partners, increasing demand by communities (consumers) and strengthens capacities of businesses and enterprises through training and adopting the low-cost toilet designs. The DIMOND model catalyses financial investments by households and financiers (promoting sanitation loans).

**Results and Targets**

In 2019-2020, 8,209 new toilets constructed in Serengeti district. This is 41,045-people benefited and directly improved their healthily lives. 1,336,332 Euros fund mobilized from both households and financiers for sanitation loans. The project created economic opportunities to people and especially women and youth through generating 82,090 workdays. The project supported 12-sanitation business (enterprises) and more that 82,090-people directly employed in the sanitation business. Of the 401 households surveyed during end-term evaluation, the results indicated that 38% had basic service level, 22% had limited, 28% had unimproved and 12% still practiced open defecation. Hygiene service level stood at 27% basic, 28% limited and 50% no hygiene facility.

**Conclusion**

Tackling the millennium sanitation goal calls for fresh thinking and innovative approaches. Marketing sanitation, building on perceived benefits, offers a new approach to ensure that communities have access to safer services and improved livelihood. Promotion of the market base sanitation systems allows to tape the untapped opportunities towards eradicating the spread of infectious diseases.

## A46 A new integrated management system for non-communicable diseases in Iringa district council

### Agata Miselli^1^, Francesco Cavallin^1^, Rehema Itambu^1^, Katunzi Mutalemwa^1^, Emmanuel Ndile^1^, Gaetano AzzimontI^1^, Samwel Marwa^2^, Bruno Ndunguru^2^, Giovanni Putoto^3^, Michele D'Alessandro^3^, Giovanni Torelli^4^

#### ^1^Tosamaganga District Designated Hospital, Doctors with Africa CUAMM, Iringa, Tanzania; ^2^Iringa District Medical Office, Iringa, Tanzania; ^3^Doctors with Africa CUAMM, Dar es salaam, Tanzania; ^4^Policlinico Umberto I, Doctors with Africa CUAMM, Roma, Italy

##### **Correspondence:** Agata Miselli (a.miselli@cuamm.org)

**Background**

Morbidity and mortality due to non-communicable diseases (NCDs), in particular hypertension and diabetes, are growing exponentially across Tanzania. The limited availability of NCDs services and the disparity in the quality of the health care system between rural and urban areas are among the key factors for the increased burden of NCDs in the country.

Since March 2019, an integrated management system has been implemented in Iringa District Council by Doctors with Africa CUAMM, an international organization working for health system strengthening. The system implements an integrated management of hypertension and diabetes between the hospital and the peripheral health centres, and introduces the use of paper-based treatment cards.

**Objectives**

The aim of this study is to present the results of the first-28-months roll-out of the system.

**Results**

The first 28 months of roll-out included 1128 patients. Data show that: 48.1% of patients returned for the 6 months re-assessment visit during follow-up; blood pressure was at target in 47.1% of patients with hypertension and blood sugar was at target in 37.3% of diabetic patients. The majority of patients who were lost to follow-up or did not reach the targets were those without medical insurance or living in the most remote peripheries.

**Conclusion**

Our findings confirm that gaps in the control of NCDs are still large in rural Tanzania, but integrated management systems connecting primary health facilities and referral hospitals can improve care and follow-up of patients with hypertension and diabetes. Our experience may be useful for clinician and stakeholders who are involved in NCDs management in similar settings.

## A47 Hand washing practice makes perfect: schools and health care facilities in response to covid-19 disease

### Mturi James, Christossy Lalika, Anastazia Rutatina

#### Amref Health Africa in Tanzania, Dar es salaam, Tanzania

##### **Correspondence:** Mturi James (Mturi.James@Amref.org)

**Introduction**

WASH plays significant role in reducing infectious diseases. According to UNICEF, inadequate access to WASH, kills about 4,500 children and sickens thousands more every day and highly impacting school-aged children’s. Since, eruption of COVID-19 diseases, handwashing with soap remains among the best defensive against Corona virus and other infection diseases. In Tanzania, it is only 20% of schools and 50% of health care facilities have handwashing facilities with soap. In efforts to curb the COVID-19 pandemic, in May 2020 and July 2021, Amref Tanzania through the hygiene and behavioural change coalition (HBCC) project, with support from UNILEVEL/DFID provided an awareness campaigns and installed handwashing facilities (with soaps) at high populated places (schools, markets and HFCs) in three regions of Tanzania.

**Approaches**

WASH assessment in 115-primary schools and 20-HCFs in 3-Municipalities in Dar es Salaam, Mwanza and Dodoma determined WASH conditions, skills and behaviours toward prevention and control of COVID-19. Then public sensitization using edutainment strategies using famous local artist. IEC materials with different message of hand washing technique were also distributed through social media platforms and mass campaigns. Final trainings on best handwashing practices, operation and maintenance of the facilities.

**Results and Targets**

62%, 65%, 70% schools in Ilala, Dodoma and Mwanza Municipal Councils respectively, had temporally handwashing facilities. Handwashing does not have water at all time and therefore, children do not wash their hands at critical times. Estimated 5,003,500 people were reached through sensitization activities. A total of 543 water tape were constructed in 71-schools and in 7-HFCs, in which 133,922 students and 40,000 community members directly benefited.

**Conclusion**

This project provides a guiding framework for preparedness and response to the current COVID-19 global pandemic through WASH facilities and behavioural changes. On next step, guidance note and actions will be taken to prevent infection in schools, healthcare facilities and at households’ levels thorough instilling hygiene behavioural changes at young school age (agent of changes at community level); targeting establishment of school hygiene clubs.

## A48 Integrating early child development interventions into existing health and nutrition services: feasibility and cost-effectiveness: experience from nourishing the future project

### Cesare Gelso, Michele D'Alessandro

#### Doctors with Africa CUAMM, Dar es salaam, Tanzania

##### **Correspondence:** Cesare Gelso (c.gelso@cuamm.org)

**Introduction**

Research shows that investing in early interventions timed to take advantage of crucial phases of brain development can improve the lives of the most disadvantaged and vulnerable children in their societies, helping to break cycles of poverty, violence and despair (UNICEF, 2014). However, despite some progress in recent years, Early Child Development (ECD) remains a neglected issue, particularly in low-middle income countries (LMICs).

**Methodology**

*Nourishing the Future* project, implemented by Doctors with Africa CUAMM in four districts of Iringa and Njombe regions, has embraced research analyses from the *Lancet series* published in 2016, which emphasizes the relevance of existing nutrition and health services as entry points for ECD. Indeed, we consider the health sector uniquely positioned to have multiple contacts with caregivers along the early periods of the life cycle, and especially RMNCH services can be identified as crucial entry points for ECD interventions.

**Results**

The establishment of ECD corners within the health structures proved to be a useful system for integratinghealth and child stimulation services. To promote nurturing care within already existing structures at community level,the platform of the Village Health and Nutrition Days (VHNDs) has proved to be particularly effective: VHNDs are wide-ranging community events in which health, nutrition and ECD services can be comprehensively integrated and delivered by non-specialist providers, with no unbearable costs.

**Conclusion**

In order to boost these existing structures, our experienceshows that a systematic approach is required at all levels. This should include supportive laws and frameworks that prioritize ECD service provision, allocations of adequate resources for ECD interventions across all sectors, and effective ECD coordination structures at all levels.

## A49 Integrated management of severe acute malnutrition in Tanzania: experience from doctors with Africa CUAMM

### Flora Manyanda, Michele D'Alessandro

#### Doctors with Africa CUAMM, Dar es salaam, Tanzania

##### **Correspondence:** Flora Manyanda (m.dalessandro@cuamm.org)

**Introduction**

Severe Acute Malnutrition (SAM) is a life-threatening condition requiring urgent treatment. At national level, the prevalence of Global Acute Malnutrition (GAM) among children aged 0-59 months decreased from 4.8% in 2010 to 3.5% in 2018 (TNNS,2018). Joint efforts between the Ministry of Health and health stakeholders managed to maintain the prevalence of GAM to low thresholds of below 5% according to UNICEF-WHO. In this joint effort, Doctors with Africa CUAMM has implemented several IMAM projects in different Regions including Simiyu, Ruvuma, Dodoma, Iringa and Njombe. Our intervention focuses on supporting national programs and policies on maternal and child health, coordination and support at facility level, strengthening the link between community and facility and integration of nutrition with multi-sectoral sectors like WASH, Agriculture and ECD due to its multi-factorial causes

**Methods**

The projects use training and on job mentorship approach to improve the quality of IMSAM activities at community and facility level: screening of children under five years, management of SAM cases, data collection and reporting. Nutrition assessment including mid- upper arm circumference, oedema and weight for height were done from all entry points including RCH, OPD, IPD, CTC and house hold visits. However, Village Health and Nutrition Days in the community found to be effective in capturing large number of beneficiaries at a time and it is also a great platform for interaction between community and the health system. Treatment was offered according to Tanzania Integrated Management of Acute Malnutrition (IMAM) guideline.

**Results**

In the last five years, projects were able to cover 1221 villages, 269 Health facilities and also trained 298 Health Care Workers and 2835 Community Health Care Workers to improve the quality of malnutrition services.

**Conclusion**

Community-based approach provides timely detection of severe acute malnutrition and treatment for those without medical complications at home. The combination of facility and community-based approaches for those malnourished children with medical complications minimize the risk of death.

## A50 Financial and social consequences of TB; what lessons learned from a qualitative study in Mbeya & Songwe regions

### Kilima Stella^1^, Mubyazi Godfrey^1^, Moola Aneesa^2^, Sabi Issa^3^, Mwanyonga Simeon^3^, Nyanda Elias^3^, Evans Denise^2^

#### ^1^National Institute for Medical Research, Dar es salaam, Tanzania; ^2^Health Economics and Epidemiology Research Office, Wits Health Consortium, Johannesburg, South Africa; ^3^Mbeya Research Centre, National Institute for Medical Research, Mbeya, Tanzania

##### **Correspondence:** Kilima Stella (stellakilima@yahoo.com)

**Introduction**

Tuberculosis (TB) as one of the leading killer infectious diseases in the world. This is in recognition of the statistics indicating that, TB is responsible for approximately 10 million people who are infected by it, among whom about 1.6 million dies of it each year globally (WHO, 2018). The global TB reports consistently point the African continent as the most highly burdened of all continents. However, within the African continent alone, the southern part of sub-Saharan Africa (SSA) seems to suffer more than the northern part.

**Objective**

To assess the financial and social consequences of TB in Mbeya and Songwe regions in Tanzania; a qualitative study

**Methods**

This qualitative study was conducted on TB patients, their relatives, TB survivors, and Health care providers. A total of 46 interviews was conducted to participants aged 18 years and above. KIIs and FGDs were conducted in private venues in Kiswahili local language by PhD student and trained researcher, the participants were asked permission to audio-recorded. Audio files were transcribed verbatim and translated into English. Data were analyzed thematically using NVivo version 11.

**Results**

A total of 09/12 patients and 8/12 TB survivors declare facing financial and social consequences before treatment, during and even after treatment it took them more than six months to return to their normal condition. This was due to the fact that all of them started treatment late and the late initiation of TB treatment was caused by not knowing the symptoms of TB, lack of enough money for paying a hospital bill, some choose to continue with economic activities in order to get money for the basic needs rather than going to the hospital. During treatment, TB patients are unable to engage in any economic activities because of the severity of the disease. After treatment, some of the patients were unable to return to their normal activities because of losing their network and some due to severe treatment they were facing some complications which made them unable to perform their daily activities as they used to do.

**Conclusion**

In conclusion, it is clear that Poverty and Tuberculosis are two-sided of the coin, because TB is responsible for poverty and vice versa.

## A51 Club model implementation in art provision among PLHIV: test & treat project in Shinyanga and Simiyu regions

### Nock Shagata, Collin Frank, Justice Minofu, Atim Molinari, Mwanaid Mfanga, Giada Corona, Michele D'Alessandro

#### Doctors with Africa CUAMM, Dar es salaam, Tanzania

##### **Correspondence:** Nock Shagata (m.dalessandro@cuamm.org)

**Background**

Since 2016, the Test & Treat project has been implemented in Shinyanga and Simiyu Regions by Doctors with Africa CUAMM, an international organization working for health system strengthening. The project aims at enhancing the availability and accessibility of HIV services through strengthening targeted HIV testing and counselling services and providing client-centered antiretroviral therapy (ART).

**Methodology**

Currently worldwide, HIV services have been differentiated and client-centered, thus simplifying and adapting HIV services across the cascade to reflect the preferences and expectations of various groups of people living with HIV (PLHIV), while reducing unnecessary burdens on the health system.

The Test & Treat project is implementing the CLUBs model in ART provision, whereby stable clients are given ART at their communities through healthcare worker-managed groups. This service model aims to reduce unnecessary burden on the health facility and to increase clients’ adherence and retention in care. The CLUBs are composed of 25-30 stable clients living within 3-5 km from the CLUB meeting site.

**Results**

Since July 2018, 74 CLUBs headed by 4 Care and Treatment Centers (CTCs) have been established with a total of 1447 patients enrolled. At the moment, there are only 12 lost to follow-up and 16 deaths have occurred, mostly not HIV-related (data updated to June 2021).

**Conclusion**

We believe that in a context like Tanzania, the CLUB model can be considered a valid alternative as one of the differentiated ART provisions, to decongest hospitals and health facilities and at the same time to guarantee treatment to the majority of PLWH in resource-limited settings. Furthermore, by bringing the services closer to patients, there is a saving of money and time for the clients which lead to an improvement in adherence to care.

## A52 Empowering women to access maternal healthcare services through a digital momcare wallet

### Liberatha Shija, Johnson Yokoyana, Neema Massawe

#### PharmAccess Foundation, Dar es salaam, Tanzania

##### **Correspondence:** Liberatha Shija (l.shija@pharmaccess.or.tz)

**Introduction**

Although ‘free’ maternal care policy exists, out-of-pocket payments remain the predominant mode of health care financing in Tanzania. Expectant mothers from low-income households often do not seek skilled birth attendance to avoid the large costs associated with delivery. Digitized maternal health care and financial system is revolutionizing maternal and newborn care in Tanzania. PharmAccess Foundation in collaboration with Selcom have developed a digital solution that tracks the journey of expectant mothers and use a digital ‘closed’ wallet to access services.

**Methodology**

Enrolled women are provided with an electronic card which has virtual money to pay for maternal services at contracted facilities. The card with QR code stands as unique identifier and must be presented during each visit along the pregnancy journey. Midwife put the QR CODE on mother’s card and scan the QR CODE using a tablet. Then she enters all services provided and the woman pay her bills by swapping the card at the POS machine. SMS on amount utilized and remaining balance is sent back to the woman.

**Results**

By 31st July 4,500 women were using a digital wallet. ANC profile at first visit improved from 45% in February to 86% in July and BP, Weight and HB checks at every visit improved from 36% to 91%. Adherence to ANC visit reached 3.74 exceeding the target of 3 visits. Additionally, institutional delivery improved to 67% compared to 52% at the start of the project and PNC within seven days after delivery reached 31% from 25%.

**Conclusion**

Digitalization of the payment process improves efficiency and in turn improves quality of services provided. Male involvement has been enhanced and women have been empowered to seek maternal health care and request for expected services that were not offered.

## A53 Kap study on hand hygiene towards covid-19 among adults visiting the main market in Mikindani, Mtwara region

### Mwanaisha D Zayumba, Aibora Samali

#### St Joseph University College of Health and Allied Sciences, St Joseph Universy in Tanzania, Dar es salaam, Tanzania

##### Mwanaisha D Zayumba (mwanaishazayumba@gmail.com)

**Background**

China reported the Novel Coronavirus at the end of the year 2019 which was, later on, declared a Pandemic by the World Health Organization. Proper hand hygiene was identified as one of the simplest most cost-effective Coronavirus Disease -19 control and prevention measures. It is therefore very important to understand the compliance of the community to hand hygiene.

**Objective**

This study aims to examine the knowledge, attitude, and practice of hand hygiene towards COVID - 19 among adults visiting the Main market in Mikindani municipal, Mtwara region.

**Methodology**

A descriptive cross-sectional study was conducted at the Main market, Mikindani Municipal, Mtwara for one month and simple random sampling approach was applied. Structured questionnaire was used to assess their knowledge and attitude while checklist was used to assess their practice towards hand hygiene. Analysis was done by using chi square method.

**Result**

A total of 100 participants were recruited with a mean age of 29.5 years (Standard deviation = 15.41). 50.85% were men and 49.15% were female, 43.22% are self-employed etc. 74% were knowledgeable about hand hygiene, 94.07% of the participants had a positive attitude towards hand hygiene and only 13.56% of all participants had good practice on hand hygiene. After adjusting for other factors, the association was observed between knowledge on hand hygiene with sex (P-value = 0.021). Attitude towards hand hygiene and occupation (P-value = 0.021) as well as practice on hand hygiene with sex (P-value = 0.032).

**Conclusion(s)**

Despite the good knowledge and positive attitude on hand hygiene, there is poor practice due to barriers like lack of soap and clean water. More efforts are required to ensure that people are being emphasized on proper practice of hand hygiene through public health involvement by better designing and implementation strategies.

## A54 Factors influencing health insurance coverage among patients at Bukoba regional referral hospital

### Johnston K Barongo, Elena Generalova

#### St Joseph University College of Health and Allied Sciences, St Joseph Universy in Tanzania, Dar es salaam, Tanzania

##### **Correspondence:** Johnston K Barongo (jbkagumisa@gmail.com)

**Background**

Avoidance of catastrophic health expenditures and the need to strive towards universal health coverage have called for the need to develop models of health insurance. Health insurance coverage in Tanzania has not met desired goals with only 9% of the total Tanzanian population enrolled to national Health Insurance Fund (NHIF) and only 2.1 million households enrolled to Community Health Fund (CHF). This study aimed to determine the factors influencing health insurance coverage among patients at Regional Referral Hospital, Kagera Region.

**Methods**

Descriptive cross sectional institutional based study involving 168 participants was conducted in November 2020. Study participants who were selected randomly. Data was collected using structured questionnaires and analyzed using IBM SPSS Statistics Version 25.

**Results**

Enrollment to health insurance services was found to be 69.0% of all respondents; majority being government employees. More than three quarters (91.38%) of respondents covered by health insurance belonged to National Health Insurance Fund (NHIF) and only 5.17% belonged to Community Health Fund (CHF). Only 98.2% of the respondents were aware of health insurance. Significant relationship was found between enrollment to health insurance and age (p-value=0.000), level of education attained (p-value=0.017), occupation (p-value=0.000), average monthly income (p-value=0.000), health status (p-value=0.000) and awareness on health insurance (p-value=0.009). However, enrollment to health insurance coverage was not significantly associated to gender (p-value=0.637), marital status (p-value=0.151) and religion (p-value=0.741)

**Conclusion**

The study concluded that enrollment to health insurance was influenced by age, highest level of education attained, occupation, average monthly income, health status and awareness on health insurance.

## A55 Illiteracy of HIV transmission and prevention methods among orphans and vulnerable adolescents in Tanzania

### Amon Exavery, John Charles, Tumainiel Mbwambo, Jacob Mulikuza, Asheri Barankena, Christina Kyaruzi, Charles Makoko, Amal Ally, Remmy Mseya, Levina Kikoyo, Deogratias Kakiziba

#### Pact Tanzania, Dar es Salaam, Tanzania

##### **Correspondence:** Amon Exavery (aexavery@pactworld.org)

**Background**

Despite evidence of notable progress in the global awareness and knowledge of HIV and AIDS, there are gaps in knowledge of HIV prevention among certain populations. This study assessed the factors associated with HIV transmission and prevention methods literacy among 10–19-year-oldorphans and vulnerable adolescents (OVAs) in Tanzania.

**Methods**

Cross sectional data (collected in 2019) from the community-based USAID-funded Kizazi Kipya project implemented in 25 regions in Tanzania were used. Illiteracy was defined as having incorrect knowledge of one or more HIV transmission and prevention methods (i.e., HIV can be transmitted through unprotected sex, sharing injecting needle, mother-to-child during pregnancy, delivery, and breastfeeding; and prevention through abstinence, faithfulness, consistent condom use). illiteracy of at least one of the methods was coded as 1 and absence thereof as 0. Data analysis involved multilevel mixed-effects logistic regression.

**Results**

The study analyzed 179,341 OVAs aged 10-19 years. 51.5% were female, and 71.3% had female caregivers. Of all the OVAs, 32.4% were illiterate of one or more of the HIV transmission and prevention methods. The likelihood of illiteracy increased among OVAs: with male caregivers (OR=1.12, 95% CI 1.04-1.21), with disabled caregivers (OR=1.38, 95% CI 1.14-1.67), with HIV status undisclosed caregivers (OR=1.59, 95% CI 1.45-1.74), with divorced caregivers (OR=1.62, 95% CI 1.47-1.77), changed caregiver in the past year (OR=1.34, 95% CI 1.13-1.59), and lived in urban (OR=1.26, 95% CI 1.18-1.34). The likelihood of illiteracy declined among OVAs: with older age (15-19 years) (OR=0.51, 95% CI 0.49-0.53), with educated caregivers (primary: OR=0.77, 95% CI 0.71-0.84; secondary: OR=0.74, 95% CI 0.62-0.90), with HIV positive caregivers (OR=0.86, 95% CI 0.79-0.92), and from moderate hunger households (OR=0.76, 95% CI 0.71-0.82).

**Conclusion**

Nearly one third of OVAs in Tanzania are illiterate of one or more HIV transmission and prevention methods, highlighting a need to advance and target HIV transmission and prevention education to adolescents, especially those who are orphaned and vulnerable, who live with male caregivers, have disabled caregivers, live with divorced or widowed caregivers and other detrimental caregiver, household, and residence contexts which drive the illiteracy.

## A56 Maltreatment of orphans and vulnerable children: findings from the USAID Kizazi Kipya project in Tanzania

### Amon Exavery, John Charles, Tumainiel Mbwambo, Jacob Mulikuza, Asheri Barankena, Christina Kyaruzi, Charles Makoko, Amal Ally, Remmy Mseya, Levina Kikoyo, Deogratias Kakiziba

#### Pact Tanzania, Dar es Salaam, Tanzania

##### **Correspondence:** Amon Exavery (aexavery@pactworld.org)

**Background**

Child maltreatment is a problem worldwide and has life-long severe consequences. Understanding the magnitude and factors associated with maltreatment is key to appropriate interventions. This study assessed the prevalence and factors associated with maltreatment of orphans and vulnerable children (OVC) in Tanzania.

**Methods**

This is secondary data analysis of data from 25 regions of Tanzania where the USAID Kizazi Kipya project was implemented. Child maltreatment was defined as an experience of one or more abuse categories; physical abuse, sexual abuse, emotional abuse, exploitation, or neglect by a child aged 0–19 years in the last 6 months. Multivariate analysis using random-effects logistic regression was conducted.

**Results**

The study population comprised 418,923 OVC aged 0–19 years, 51.0% of whom were female, and 71.0% had female caregivers. Overall, 1.6% of the OVC experienced some form of abuse. Several factors were noted to be associated with reporting or experiencing abuse. OVC who received parenting intervention were more likely to report abuse as compared to those who received no parenting intervention. Abuse also varied by OVC characteristics: sex, age, school enrollment status and place of residence as well as by their caregiver characteristics: sex, marital status, and disability status.

**Conclusions**

About 2% of the studied OVC had experienced at least one type of abuse in the last six months, with OVC who were reached by the parenting intervention being more likely to report abuse than those who were not. While there may be many cases of unreported abuse due possibly to inability to recognize them, these factors could inform targeted interventions towards ending child abuse in Tanzania.

## A57 Determine the uptake of contraceptives among adolescents and youth in Tanzania

### Charles Mushi^1^, Kenneth Owino^1^, Msira Mageni^1^, Mary Joseph^1^, Tumaini Kiyola^1^, Lena Mfalila^1^, Edith Mboga^2^, Catherine Kenneth^2^, Rehema Panga^2^, Edith Kijazi^2^, Pilly Makame^2^

#### ^1^Johs Hopkins Program for International Education in Gynaecology and Obstetrics, Dar es salaam, Tanzania; ^2^Ministry of Health in Tanzania, Dar es salaam, Tanzania

##### **Correspondence:** Charles Mushi (Charles.Mushi@jhpiego.org)

**Introduction**

TCI uses service statistics data from the Health Management Information System (HMIS) to estimate family planning client volume and to model annual mCPR growth. While all TCI countries have FP client data available for women 15-49, there is limited data regarding client characteristics (i.e. age, marital status, and parity). In most cases, this information is collected at the facilities and recorded in registers/logbooks but are not reported up to the HMIS database. This presents a challenge in making data for adolescents and youth visible. Without age-disaggregated data, it is difficult to monitor progress on contraceptive uptake among this age group. With focus on estimation of FP users by age group 15-19, 20-24, >24, data were collected from June 2018-October 2020 (30 months).

**Methods**

71 health facilities were selected purposely, 55% being AY facilities, and 45% non-AY facilities Purposive and simple random sampling technique were used to select classic and layering health facilities. 11 TCI supported geographies were sampled respectively. Aggregated FP attendance data summarized in program specific form. 71 health care providers were oriented on AY tool and real-time-reporting.

**Results**

Key findings of the survey shows an increase of FP uptake among AY by 123% from the baseline data (43,515). It was also revealed that FP uptake on LARC, increased by 128% (87,272) from 38,249. In terms of comparison, the findings indicates that, layered facilities had 131% change, compared to classic facilities which were 111% change.

**Discussion & Recommendations**

For the purpose of estimating FP users by age category using current disaggregation variables available in the HIMS registers, DHIS2 data summary, should be disaggregated by age (15-19, 20-24, 25+) and by method (condoms, pills, injectable, emergency contraception, IUD, implants, male sterilization, female sterilization).

## A58 Xpert MTB/RIF ultra cycle threshold values as a predictor of sputum culture conversion during TB treatment

### Beatrice Ngaraguza^1^, Julieth Lalashowi^1^, Nyanda E Ntinginya^1^, Ombeni Chimbe^1^, Issa Sabi^1^, Daniel Mapamba^1^, Bariki Mtafya^1^, Willyhelmina Olomi^1^, Andrea Rachow^2^, Robert Wallis^3^

#### ^1^Mbaya Researcg Centre, National Institute for Medical Research, Mbeya, Tanzania; ^2^Division of Infectious Diseases and Tropical Medicine, University Hospital, LMU Munich, Germany; ^3^The Aurum Institute, Johannesburg, South Africa

##### **Correspondence:** Beatrice Ngaraguza (bngaraguza@nimr-mmrc.org)

**Background**

The Xpert MTB/RIF Ultra (Xpert Ultra) assay offers rapid diagnosis of tuberculosis (TB) and quantitative estimation of bacterial burden through Cycle threshold (Ct) values. We assesed the association of Xpert Ultra Ct values at the time of TB diagnosis in predicting sputum culture conversion at month 2 and 6 post TB treatment initiation among adult pulmonary TB (PTB) patients in Mbeya Tanzania.

**Methods**

Information were obtained from adults PTB patients participating in the NAC-TB substudy of TB Sequel cohort, which examine if oral N-acetylycysteine (NAC) could restore Glutathione and prevent lung injury in TB patients. Participants were enrolled at the time of TB diagnosis and were followed up for at least 2 years. About half of the partcipants received NAC in addition to standard TB and HIV therapy. Information on demographics, HIV status, Xpert ultra, and culture results at enrollment, and subsequent culture results at month 2 and 6 were extracted from the NAC-TB database. The association between Xpert Ultra Ct values and culture conversion at month 2 and 6 were determined using multivariable logistic regresion.

**Results**

Between March 2019 and August 2020, 90 participants were enrolled. The median age was 34 (IQR: 28-40) years, 64(71.9%) were male, and 23(25.6) were HIV positive. The median Xpert Ultra Ct values was 16.2 (IQR; 16.1-16.3). At month 2 and month 6 (end of TB treatment), 45/87 (51.7%) and 5/73(6.9%) participants remained culture positive respectively. Xpert Ultra Ct values at the time of TB diagnosis showed no association with sputum culture conversion at month 2 and 6 during TB treatment.

**Conclusion**

Over half of adult TB patient remain culture positive at the end of intensive phase of TB treatment. Xpert Ultra Ct values at the time of TB diagnosis shows no association with sputum culture conversion during TB treatment.

## A59 Knowledge and attitudes towards prevention of mother to child HIV transmission

### Abel Kibiki, Elizabeth Popova

#### St Joseph College of Health and Allied Sciences, St Joseph University in Tanzania, Dar es salaam, Tanzania

##### **Correspondence:** Abel Kibiki (ados.sjchas@gmail.com)

**Background**

Mother to child transmission of HIV (MTCT) is one among the mode of HIV infection transmission in most of African countries and the common way for children to become infectedwith HIV. Good knowledge about HIV transmission can lead to implementing prevention of MTCT programs and help to achieve goals of eliminating and decreasing new cases of HIV in children.

**Objective**

The study aimed to assess knowledge and attitude on MTCT of HIV among pregnant women attending Reproductive and Child Health clinic.

**Methodology**

100 pregnant women attending Reproductive and Child Health clinicat Lugoda Hospital, Mufindi district, Iringa regionwere selected randomly. Data was collected using structuredQuestionnaires and analyzed by IBM SPSS Statistics Version 25.

**Results**

Most of respondents were aware that there is possibility of mother to child transmission of HIV, but they didn’t have knowledge about the ways of transmission. Almost all respondents were aware that there is possibility of prevention of MTCT of HIV; but only half of them mentioned giving ARVs to the mother, only few - giving antiretroviral medicineto the new born, and delivery by cesarean section as preventive measures. Generally, only 27 % of pregnant women had adequate knowledge on prevention of mother to child transmission of HIV. More than half of respondent had a positive attitude towards prevention of mother to child transmission of HIV.

**Conclusion**

Health workers have a task to continue and increase effort in providing education about understanding the way of transmission of HIV.

## A60 Assessment of knowledge, attitude and practice on nutritional risk factors on hypertension among adults at Wazo ward, Kinondoni district, Dar es salaam, Tanzania

### M Kilaja, E Popova

#### St Joseph College of Health and Allied Sciences, St Joseph University in Tanzania, Dar es salaam, Tanzania

##### **Correspondence:** M Kilaja (ados.sjchas@gmail.com)

**Background**

Hypertension contributes significantly to cardiovascular and renal failures. Controlled and prevented by lifestyle modifications, it becomes to be the challenges in different communities due to the lack of nutritional knowledge, attitudes and practices.

**Objective**

The study aimed to determine the knowledge, attitude and practices on nutritional risk factors on hypertension among adults at Wazo ward, Kinondoni district, Dar es salaam region.

**Methodology**

100 people aged above 18 years living at Wazo ward, Kinondoni district, Dar es salaam region were involved randomly. Data was collected using structured Questionnaires and analyzed by by Epi Info version 7.2.

**Results**

About 66% of respondents had knowledge on hypertension and that normal blood pressure exists. Among represented risk factors of hypertension, 82% of respondents underlined obesity, 79% - sedimentary life style, 73%- high fat intake, only 61%- high salt intake, 58%- smoking, and 45% genetic factor. Knowledge on risk factors on hypertension was significantly associated with education. Only 58% of respondents considered that regular checking of blood pressure to adult is very important for early treatment and for the health life. About 72% respondents thought that reduction of high salt intake is important. A lot of people had habit to add extra salt in their meal and consumed fatty food. Only few people did not use high salts and fat foods because of risk for disease.

**Conclusion**

People in rural and urban areas should be given education on good dietary practices towards prevention and control of hypertension. Every public area should have available health personnel for nutritional health education.

## A61 Feeding practice in in Ruvuma region, Tanzania

### Shanel G Komba, Elizabeth V Popova

#### St Joseph College of Health and Allied Sciences, St Joseph University in Tanzania, Dar es salaam, Tanzania

##### **Correspondence:** Shanel G Komba (ados.sjchas@gmail.com)

**Background**

Inappropriate feeding practices for children are the major contributor to poor nutrition status among children under five years in Tanzania.

**Methods**

We aimed at assessing maternal knowledge, attitude, and practice towards feeding practices among mothers of under-five years’ children at Langiro Ward, Mbinga district in Ruvuma region. Pre-tested structural questionnaires were used. Calculation of proportion and other statistical tests were done by the SPSS program.

**Results**

Out of 100 mothers, 64% were primary school leavers meanwhile 79% were housewives (not employed), 83% had a daily income of less or equal to 500Tshs. 88% of respondents had poor knowledge of feeding practices over their children. 84% of mothers had a negative attitude toward complementary feeding. 82% of women were not covered with medical/health information from health practitioners or mass media. 74% started complementary feeding at six months, 71% of all respondents started with porridge. 53% were breastfeeding less than 24months while 42% reached 24months. about 66%decreased food quantity to their children when fell sick. 33% responded that cultural norms had a positive contribution to the child’s nutrition. 80% of respondents bided with the answers that suggested milk, vegetables, and fruit juices as frequent complementary foods. 52% had three meals in the family and the rest had less, 93% did not practice pre-lacteal feeding. Only 59% of mothers continued breastfeeding alongside complementary feeding.

**Conclusion**

These results are generally terrible hence all members of the community, policymakers, NGOs, local government should work in collaboration with health officers, health centers, and other health institutions to remove the downsides of mothers on knowledge, attitudes, and practices about appropriate complementary feeding.

## A62 Momcare project contribution on performance of ANC indicators at Bonga health centre. Can this be sustained?

### Fadhil Kalokola

#### Bonga Health Centre, Manyara, Tanzania

##### **Correspondence:** Fadhil Kalokola (fadhilmusa81@yahoo.com)

**Background**

ANC provision as per standard guidelines is the major factor in achieving safe journey of pregnant mother, reducing maternal and perinatal morbidity/mortality. However, facilities have been facing challenges on supply of maternal commodities to meet ANC requirements. This contributed to low performance on most of these indicators. MomCare project under PharmAccess implemented at the Bonga Health Centre since April 2020 has made positive changes on maternal and newborn service delivery through its improvement on commodity availability.

**Methodology**

The analysis was done on six major ANC indicators to compare the year before and after MomCare project. The indicators under analysis were, new pregnant women starting ANC, New ANC visit below 12 wks, ≥4ANC visit before delivery, total ANC visit including revisit, institutional delivery and PNC within 48 hours. The tools used were RCH HMIS Books and DHIS2 database.

**Results**

Changes on indicators under study were quantified in numbers and percentage and in reference to facility targets. There was clear increase in scores on all indicators between the review period as follows. New ANC attendance (106.6% to 115%), New ANC attendance <12 weeks of gestation age (57.1% to 81.4%), ≥4 ANC visit before delivery (115.2 to 225.9%), total visit including revisit (72.6% to 112.6%), HF delivery (78% to 91.4%) and PNC in 48 hours (78.3% to 108.8%).

**Conclusion**

ANC maternal indicators are critical areas to assess the quality of a pregnant mother. To consistently achieve the facilities, need to have a guaranteed supply of essential commodities to enable them deliver services according to set guidelines. MomCare has shown how this is possible the question that remains is how these achievements can be sustained. There is a need to learn and support the model used in MomCare for facilities to sustain quality maternal and newborn health care delivery.

## A63 Use of social networks strategy (sns)to increase case finding in HIV testing and counselling among FSW & MSM

### John Emma, David Ally, Amos Hanifah, Innocent Proches, Mboggo Eric, Sarah Mununa, Kilimba Edwin, Mponzi Victor, Muya Aisa

#### Amref Health Africa, Dar es salaam, Tanzania

##### **Correspondence:** John Emma (Emma.John@Amref.org)

**Background**

The country has made significant progress toward HIV control among key population (KP) with the prevalence reported to have declined among female sex workers (FSW) from 26% in 2014 to 15% in 2018, while that of male sexual workers (MSM) from 25% in 2014 to an estimate of 8.4% in 2018.Despite the effort made which include conduct HTS during community KP outreach services in hotspots, use of Peer model approach however HIV prevalence among this group continue to be higher 2 times than general population. This can be contributed to challenges in operationalization of several strategies owing to the high levels of stigmatization and discrimination toward key populations This abstract seeks to unleash the efficacy of using social networks strategy to increase HIV case finding among FSW and MSM through the support of PEPFAR funds to Amref Tanzania’s Afya Kamilifu project

**Methods**

Beginning of July 2020, the project introduced a new strategy of social networks strategy (SNS) among FSW and MSM to ensure increase of KP case findings in Tanga. Project used health care workers (HCW) already trained in delivering KP friendly services and Peer based volunteer to implement SNS. This was followed by recruitment of seeds/social client with the following eligibility criteria: (1) male/female, age 18 years or older; (2) reporting male to-male sex behavior (anal or oral sex), multiple sexual partners in the past 12 months; (3) having at least 3 genuine friends who were MSM or FSW in their social or sexual network (4) KPs newly or previously diagnosed with HIV. Each seed/social client is required to elicit a maximum of 3 MSM/FSW friends with similar behaviors referred as social contact with detailed contact information. SNS were routinely collected and monitored every month. Data was then evaluated to assess the outcome of the strategy.

**Results**

Between July and December 2020 a total 176 seeds were selected in Tanga region, a total of 457 social contact (MSM & FSW friends) were elicited, 429 (94%) social contact were tested, 106 POS identified which is 25% yield and 104 (98%) initiated ART.

**Conclusion**

Hence the findings suggest that the social networks strategy could be an effective and powerful tool for optimizing case finding among KPs (MSM and FSW) hence improve yield in HIV testing and Counselling Services.

## A64 Demand side financing for maternal healthcare enhances quality of care provision-momcare project experience

### Johnson Yokoyana, Liberatha Shija, Anunsiatha Mrema, Theodora Kiwale, Jonia Bwakea, Lemali Mbise

#### PharmAccess International, Dar es salaam, Tanzania

##### **Correspondence:** Johnson Yokoyana (l.shija@pharmaccess.or.tz)

**Introduction**

In Tanzania reproductive and child health services are largely financed through supply side financing mechanisms. Some of the limitations of this model are inability to target the poor, lack of user choice and absence of linkages between provider payments and quality of care. This brings a need for an alternative financing mechanisms, which can target scarce resources to those who cannot afford to pay and improve quality. PharmAccess is implementing the demand side financing model for maternal care in Kilimanjaro and Manyara region with aim of improving access and utilization for better maternal and new born outcome. This is done through allowing pregnant mothers to access services through a predefined care bundle in form of electronic ring fenced wallet. Mother use their wallets to pay for services then facilities get reimbursed upon verification for the quality of services delivered.

**Methodology**

Analysis was done on service utilization data from 40 facilities at their first month and sixth month of participation in the project. The aim was to evaluate improvement on two key risk monitoring indicators which are ANC full profile uptake at first ANC visit and consistent measure of weight, Hb and Blood Pressure together at a visit over the same period.

**Results**

The ANC full profile access improved from 30% at first month to 90% at six month of facility participation in the project. Consistent uptake of weight, BP and Hb together at a visit improved from 42% to 91%.

**Conclusion**

The demand side financing model can significantly improve service delivery as motivates effective service delivery. Quality based payment stimulate inter-facility competition to win client satisfaction which result in overall better outcome. Government and funders need to see the potential of this model in effort to curb maternal and newborn morbidity and mortalities.

## A65 Gender equality as a means to improve maternal and child health in Simiyu region Tanzania

### Jane Tesha^1^, Agatha Fabian^2^, Frida Ngalesoni^1^, Serafina Mkuwa^1^, Gasto Frumence^3^

#### ^1^AMREF Health Africa, Dar es salaam, Tanzania; ^2^University of Dodoma, Dodoma, Tanzania; ^3^Mumbili University of Health and Allied Sciences, Dar es salaam, Tanzania

##### **Correspondence:** Jane Tesha (jane.tesha@amref.org)

**Background**

Amref Health Africa, through support from Global Affairs Canada, examines whether measures of gender-based roles and responsibilities, decision-making power, access to, and control over resources, and social norms are associated with maternal and child health outcomes in Tanzania. A Gender Need Assessment (GNA) was conducted in five districts in Tanzania’s Simiyu Region in relation to improving the infrastructure, supply, quality and demand of integrated Reproductive, Maternal, Newborn and Child and Adolescent Health (RMNCAH), Nutrition, and Water, Sanitation and Hygiene (WASH) services. The assessment identifies gender equality as a fundamental driver not only on maternal, but also child health.

**Methodology**

The qualitative assessment explored themes related to gender-based roles and responsibilities, norms, decision-making power, access to, and control over resources. Primary data gathered through in-depth interviews of Key Informants and Focus Group Discussions (FGDs) with beneficiaries were triangulated using secondary data obtained through a review of existing project documents, policies, strategies and laws related to gender equality and RMNCAH, WASH and Nutrition in the country.

**Results and Lesson Learned**

Majority of participants mentioned the key drivers that influencing gender inequalities as highlighted below:

Overall, women and girls have more roles and responsibilities at the household and community level than men and boys, and men and boys’ roles are more valued than women and girls’ roles and responsibilities. Some participants reported as follow; “A woman is a housewife, a man is a decision-maker, women’s job is to give birth to children for me to have a big family” (FGD –Community leader - Mkula village, Busega DC)

**Conclusion**

Based on the findings it can be concluded that: RMNCAH services in Tanzania need to take into consideration the unique barriers faced by women and adolescents that are the result of gender norms and age-related constraints.

## A66 Quality improvement initiatives to improve EAC among HIV clients with high viral load in Tanga city

### Christina Mosha^1^, Andrew Kigombola^1^, Abubakar Maghimbi^1^, Simon Mkawe^2^, Faraja Gabriel^2^, Sarah Kweyamba^2^, Brown Mwakibambo^2^, Eric Mboggo^2^, Kilimba Edwin^2^, Mponzi Victor^2^, Muya Aisa^2^, Florence Temu^2^

#### ^1^Afya Kamilifu Project, University of Maryland Baltimore, College Park, United States of America; ^2^Afya Kamilifu Project, Amref Health Africa, Dar es salaam, Tanzania

##### **Correspondence:** Christina Mosha (cmosha@mgic.umaryland.edu)

**Background**

Since 2016, Tanzania had adopted World Health Organization (WHO) guideline that recommend ‘’Test and Start ” strategy aiming at HIV testing and ART initiation to all HIV positive to ensure viral suppression. With the current viral suppression in Tanga city at 97%, 453 clients have high viral load. These clients are usually initiated Enhanced Adherence Counseling (EAC) to improve adherance, address barriers and achieve re-suppression. We present a quality improvement (QI) initiatives which improved EAC cascade among clients with high viral load in CTC facilities in Tanga city council.

**Approach**

Following both the central and on job training of CTC staff, all high volume sites in Tanga city were enrolled in the EAC improvement initiative (N=4). Following a root cause analysis; poor documentation, not all clients being registered in the EAC, inadequate follow up and inactive multi-disciplinary teams (MDT) were identified as major gaps. On monthly basis, HVL results of clients were reviewed, clients with high viral load (>1000 cp/mL) were identified and enrolled in the EAC and closely followed by the re-activated facility MDT for three months. Repeat viral load was performed to check for re-suppression or guide a switch to a second line. Progress was monitored on a monthly basis and shared across facilities involved in this QI. Through this QI approach we were able to identify all clients with high viral load and enroll them in EAC. Data was triangulated between lab register, CTC database and EAC register to ensure all clients are registered and followed.

**Results**

With the intervention implemented for thirteen months, initiation of EAC to clients with high viral load improved from 75% in October 2019 to 100% in October 2020.

**Conclusion and recommendation**

The QI initiative involving multiple sites has helped to improve initiation of EAC among clients with high viral load in Tanga city. The approach enabled the QI teams to work together in identification of gaps and institution of tailor-made initiative to improve EAC initiation through cross-learning.

## A67 The role of targeted mentorship to improve ipt initiation and completion rate in Tanga, and Znz islands

### Andrew Kigombola^1^, Abubakar Maghimbi^1^, Wanze Kohi^2^, Eric Mbogo^2^, Edwin Kilimba^2^, Victor Mponzi^2^, Aisa Muya^2^, Florence Temu^2^,

#### ^1^Afya Kamilifu Project, University of Maryland Baltimore, College Park, United States of America; ^2^Afya Kamilifu Project, Amref Health Africa, Dar es salaam, Tanzania

##### **Correspondence:** Andrew Kigombola (akigombola@mgic.umaryland.edu)

**Background**

Tuberculosis remains a public health problem worldwide, affecting 10 million people in 2019 and people living with HIV (PLWHIV) remain very susceptible to TB. Use of Isoniazid Preventive Therapy (IPT) for 6-12 months among PLWH has shown to reduce the risk of TB by 33%. Besides all the benefits of the IPT, the initiation and completion rate were very low in Tanga and Zanzibar, in October 2018 with completion rate of only 31%. Afya Kamilifu project in Tanga, Simiyu and Zanzibar Island employed targeted mentorship across facilities aiming to improve IPT initiation, completion and TB diagnosis cascade. We evaluated the impact of targeted mentorship to improve IPT indicators.

**Approach**

Deployment of district-based mentors was agreed as an intervention for improvement. District based mentors were oriented in IPT and TB diagnosis cascade as per the national standards. Emphasis on IPT eligibility and documentation was emphasized as these were determined as major hurdles affecting IPT performance. Deployment of mentors started in March 2019. At least two to three mentors were deployed in each district on monthly basis. Data and documentation errors were corrected in real time at the facility. As part of a monthly monitoring, among other indicators, data on IPT completion and initiation were collected and displayed in the quality improvement dashboard.

**Results**

From baseline of 31% recorded at the baseline, IPT completion rate continued to improve in regions on monthly basis to 93% in June 2021. Erratic performance observed in the first year was contributed by INH stock outs, treatment interruptions and limited staff knowledge on IPT initiation and completion criteria. Also, the proportion of clients eligible and initiated IPT increased from 25% to 91%.

**Conclusion**

Targeted mentorship coupled with close real time monitoring has resulted into an improvement of IPT completion rate across facilities. Also, the mentorship built the capacity of health workers in identifying and initiating IPT to the eligible clients. To maintain performance, INH stock is monitored closely, and drugs redistributed if needed in addition to retention to care strategies. Use of district-based mentors ensured sustainability and transition to the CHMTs.

## A68 Reaching out vulnerable and risk groups for tuberculosis through outreach activity in Amref health Africa Tz

### Rose Olotu^1^, Godson Maro^1^, Godwin Munuo^1^, Michael Machaku^1^, Aisa Muya^1^, Florence Temu^1^, John Msaki^2^, Paschal Wilbroad^3^

#### ^1^Amref Health Africa Tanzania, Dar es salaam, Tanzania; ^2^National Tuberculosis and Leprosy Programme Tanzania, Ministry of Health, Dodoma, Tanzania; ^3^USAID Tanzania, Dar es salaam, Tanzania

##### **Correspondence:** Rose Olotu (Rose.Olotu@Amref.org)

**Background**

According to 2020 WHO TB Global Report, Tanzania misses approximately 54,166 TB cases all forms annually. Through USAID Tanzania funding, Amref Tanzania’s Afya Shirikishi project in collaboration with NTLP is implementing community-based TB services aiming at finding missing people with TB among vulnerable, underserved and at-risk population for TB.

**Approach**

Mapping of hot spot areas for the vulnerable, underserved and risk population for TB is done before the event is conducted. During the event, different approaches are used attracting people to gather together for TB screening. Systematic active TB screening through outreach services was carried out in 18 out of 54 districts throughout 9 regions supported by the project.

**Results**

About 54,140 individuals received health education about TB during outreach services. Among them 49,198 (91%) individuals were screened for TB and 18,535 (38%) were identified as presumptive TB clients, 14,969 (87%) of these people were tested for TB, 999 were bacteriologically confirmed TB (736 GeneXpert: 263 microscope). Out of diagnosed TB patients 1250 (99%) started TB treatment. The outreach showed that the slums and household visit has a high number of at risk people with undiagnosed TB 57%, market areas 17%, fisher folks 13%, miners and mining communities 7%, places where people who inject drugs like to gather 4% and traditional healers premises 1%. Majority of those who were confirmed to have TB disease were homeless men and women. Of the diagnosed TB 7(5M:2F) were MDR-TB patients.

**Conclusion**

In order to find even more of the missing TB cases, interventions targeted at vulnerable populations and raising public awareness of TB are needed to encourage more people to seek help when they experience any TB related symptoms. Interpersonal communication is needed during TB outreach services. This approach provides a room for individuals to share their challenges and get support needed.

## A69 Assessing the success of artificial intelligence and mobile health in radiological diagnosis of covid-19: a systematic review

### Atish R Shah^1^, Pooja M Karia^2^

#### ^1^Department of Biomedical Engineering, University of Strathclyde, Glasgow, Scotland; ^2^University of CapeTown, CapeTown, South Africa

##### **Correspondence:** Atish R Shah (atishshah007@gmail.com)

**Introduction**

As of 8^th^ June 2021 more,then 173 million covid-19 cases have been reported worldwide with 3.73 million deaths globally.In Africa 4.9 million cases and 132,000 deaths have been reported. Specialist medical services cannot be accessed resulting into increased turnaround times for diagnosis leading to poor patient prognosis.Artificial intelligence integrated with Mobile health as subset of telemedicine has shown that it is indeed possible to fill this gap.

**Objective**

The aim of this study is to analyse the level of success of artificial intelligence and mobile health in radiological diagnosis Covid-19 and related diseases with similar presentation.

**Methodology**

Our study reviewed peer reviewed research journal articles through desktop research methodology. The review was limited to research carried on radiological diagnosis. This study was accomplished by a structured review method to identify studies related to the identification and diagnosis of COVID-19 and related diseases with similar presentation. A systematic search strategy was developed by using previous studies and the authors’ opinions on the success of machine learning and mobile health in diagnosis.

**Results**

Our analysis showed success of artificial intelligence and mobile health in the diagnosis of covid-19 related diseases with similar presentation through the following factors: Sensitivity of detection of Covid-19 from x-rays and CT Scan images 80%- 100% and specificity of 78%-99%, it is the cheapest and safest imaging modality, and improved efficacy and reduced errors.

**Conclusion**

Artificial intelligence and machine learning,over cloud can further simplify the situation of overburdened health care profession since they can now play a major role in the expedited preliminary diagnosis of a medical image (X-ray, MRI, CT). This study elaborates ways and factors through which Artificial intelligence has been successful in the diagnosis of Covid-19 and related conditions.

## A70 The role of governance and policy in improving implementation of digital initiatives in Tanzania

### Neema Ringo^1^, Walter Ndesanjo^2^, Silvanus Ilomo^2^

#### ^1^PATH Tanzania, Dar es salaam, Tanzania; ^2^Ministry of Health, Community Development, Gender, Elderly and Children, Dodoma, Tanzania

##### **Correspondence:** Neema Ringo (walter.ndesanjo@afya.go.tz)

**Background**

Data governance and health data policies define how data flow through these systems, ensuring that data from multiple sources can be used together. Data governance establishes a landscape in which information can be safely and securely shared between systems. The Tanzania Data Use Partnership (DUP), an initiative led by the Government of Tanzania with support from PATH, is an example of how strong data governance policies are supporting the information needs of the health system and protecting individual privacy.

**Methodology**

A total of 48health sector stakeholders within the government were consulted to describe the extent of implementation of digital Health initiatives in Tanzania health sector. In addition, review of Health Sector Strategic Plan, health information systems strategies, policy guidelines, program or disease-specific strategies, and other health policies was done to inform the study.

**Results**

Previous health policy did not clearly stipulate the policy commitment in promoting evidence-based decisions in the health sector at all levels. In addition, there are several document guiding digital investments, which need to be harmonized, and the governance structures to support digital investments should work closely with Health Sector Approach mechanisms.

**Conclusion**

Central guidance is important to guide investment in digital health implementation in the health sector.Many investments have led to data availability and improved data quality but Individual data availability is still a challenge.Structures within both ministries are relevant to promote implementation of digital health initiatives in the health sector.

## A71 Door to door contact investigation: more innovation is needed to effectively finding contacts of TB index

### Rose Olotu^1^, Godson Maro^1^, Godwin Munuo^1^, Michael Machaku^1^, Aisa Muya^1^, Florence Temu., John Msaki^2^, Paschal Wilbroad^3^

#### ^1^Amref Health Africa Tanzania, Dar es salaam, Tanzania; ^2^National Tuberculosis and Leprosy Programme Tanzania, Ministry of Health, Dodoma, Tanzania; ^3^USAID Tanzania, Dar es salaam, Tanzania

##### **Correspondence:** Rose Olotu (Rose.Olotu@Amref.org)

**Background**

Contact investigation (CI) offers the opportunity for early case finding among close contacts of infectious TB patients. However, in Tanzania, although Tanzania has CI guidelines since 2016, CI is not often conducted due to minimal geographical coverage of community health workers (CHWs). Based on the WHO recommendation to actively find the missing patients, the USAID funded Amref Tanzania’s Afya Shirikishi project, is bridging this gap by engaging CHWs to closely doing TB CI among the close contacts of index patients. The aim is to reach the unreached 41% (54,166 persons) as reported by the NTLP.

**Methods**

Twice a week, the community volunteers collect index patients’ information at the DOT health facility. They visit patient households to provide TB infection control information, screen household contacts for TB, provide referrals for TB testing, and return results. The data that CHWs collect are submitted to the district TB coordinator for verification and compilation.

**Results**

From April to June 2021, 3,967 bacteriologically confirmed TB patients in 54 Amref USAID Afya Shirikishi district councils were notified. CHWs were provided with 2233 (56%) index patients for follow up. Community health workers found 7,896 close contacts of TB patients in the households, of which 7,566 (96%) persons were screened for TB symptoms. Of those screened, 3,470 (46%) were identified as presumptive TB patients and 364 (12%) diagnosed with TB. Of those diagnosed with TB, 70.1% by GeneXpert, 18.1% by smear microscopy, and the remaining 11.8% by chest X-ray and pediatric score charts. Out of confirmed TB patients (356) 98% of the TB patients started anti-TB medications. Index ratio is for CI is 1:3.5 (7896/2233).

**Conclusion**

Involvement of community members, building the capacity of village chairpersons to implement and support community TB activities will help ensure inclusion of all TB patients during contact investigation.

## A72 Impact of dolutegravir based regimen on viral load suppression in Tanga region

### Kweyamba Sarah, Mkawe Simon, Kilimba Edwin, Mponzi Victor, Muya Aisa, Mboggo Eric, Mwakibambo Brown

#### AMREF Health Africa Tanzania, Dar es salaam, Tanzania

##### **Correspondence:** Kweyamba Sarah (Sarah.Kweyamba@Amref.org)

**Background**

Tanzania had adopted World Health Organization (WHO) recommendation for using Integrase Stand Transfer Inhibitor like Dolutegravir (DTG) first line ART regimen in Tanzania since early 2019 due to less likelihood to resistance and lesser side effects hence more tolerable. DTG has been shown to improve ART compliance and viral load suppression. This study aims at assessing the impact of DTG transition on viral load suppression to CTC attending clients in Tanga Region supported under PEPFAR funds through Amref Tanzania’s Afya Kamilifu program.

**Methods**

An assessment was done using routinely conducted data from CTC2 database from July 2019 to June 2021 to review the impact of DTG transition on viral load suppression among PLHIV in Tanga region. Data points included were number of client’s eligible for viral load (VL) testing, number of clients tested and their recent viral load (VL) results within the past 12 months. The cut off for VL suppression was viral load less than 1,000 copies/ml.

**Results**

In July 2019 total clients with VL results were 32,338, Of them 7,537 (23%) were on DTG based regimen with a suppression rate of 93.6% compared to 24,801 (77%) on other ART regimen who their suppression was 88%, overall suppression rate is 89.1%. The proportion of clients with VL who were on DTG based regimen has increased from 7,537 (23%) in July 2019 to 41,950 (95.9%) in June 2021. Suppression rate among clients on DTG based regimen has increased from 93.6% to 97.8%, Suppression of clients on other ART regimen increase from 88% to 92.2% while overall improve from 89.1% to 97.6%.

**Conclusion**

Increase in DTG uptake shows a good efficacy with sharp increase in VL suppression with good safety profile where more than 95% of clients are DTG based regimen. The challenges were;- Clients on second line ART regimen were not eligible for DTG transition which is provided as single pill regimen compared to twice daily regimen. And the limitations were; - Poor adherence to DTG, pediatrics less than 20kg were not eligible for DTG

## A73 Leaving no one behind: implementing scaling up family planning programme for inclusive services in Tanzania

### Moke Magoma^1^, Kate O’Connell^1^, Danielle Garfinkel^1^, Prudence Masako^1^, Elizabeth Ngoye^1^, Deus Ngerangera^1^, Fredrick Msigallah^2^, Shahada Kinyaga^3^

#### ^1^EngenderHealth, Dar es salaam, Tanzania; ^2^Comprehensive Community Based Rehabilitation in Tanzania, Dar es salaam, Tanzania; ^3^Pathfinder International, Dar es salaam, Tanzania

##### **Correspondence:** Moke Magoma (Mmagoma@enenderhealth.org)

**Background**

Ensuring inclusive services is key to addressing inequity in health service utilization, especially for underserved society groups such as people with disabilities (PWDs). The United Nations Convention on the Rights of Persons with Disabilities and the Tanzanian Persons with disabilities act No. 9 of 2010 recognize the rights of PWDs, including on sexual and reproductive health. Importantly, PWDs have unique needs which may influence individual choices and eligibility for some family planning (FP) method choices. The Scaling up Family Planning (SuFP) programme is a five year integrated and inclusive FP programme which is implemented in 545 intensive health facilities across eight and five regions in Tanzania Mainland and Zanzibar, respectively, with provision of inclusive and integrated FP services to PWDs among its objectives.

**Methods**

A descriptive analysis was completed of Scaling up Family Planning (SuFP) programme implementation outreach service data for the period of February 2020-June 2021. PWDs were identified using a modified version of the Washington Group Criteria and clients were classified as having a disability if they reported having a lot of difficulty or were unable to do any activity in the respective disability category.

**Results**

A total of 483,984 outreach clients were reached during the period, of whom 0.8% (N=3,888) were categorized as PWDs. Of all PWDs, 73.7% were new FP clients and the remaining were continuing clients. Approximately, 73.5% of PWDs were less than 30 years (10-19 years, 10.7%; and 20-29 years, 62.8%). Physical, intellectual, and hearing impairments accounted for most encountered disabilities 26.0%, 17.8% and 21.5% respectively.

**Conclusions**

PWDs remain an underserved population segment of the Tanzanian population as regards service utilization, including SRHR. Results from the SuFP programme indicate that meeting the needs of PWDs is feasible and crucial to ensure equitable health service utilization.

## A74 Counselling and level of satisfaction with services among family planning clients in Tanzania

### Moke Magoma, Prudence Masako, Elizabeth Ngoye, Deus Ngerangera, Justin Kahwa, Ramadhan Mlange, Kate O’Connell, Danielle Garfinkel

#### EngenderHealth, Dar es salaam, Tanzania

##### **Correspondence:** Moke Magoma (Mmagoma@enenderhealth.org)

**Background**

Despite progressive improvement in family planning (FP) service uptake in Tanzania in recent years, modern contraceptive prevalence remains below the national projections. Strengthening of FP services is imperative, especially through addressing factors related to quality of services, including quality of counselling and client satisfaction.

**Methods**

A cross-sectional client exit survey was conducted involving 847 participants exiting FP services from 55 randomly selected health facilities (9 district hospitals, 19 health centers and 27 dispensaries) across eight regions of Mainland Tanzania and two regions in Zanzibar. The survey assessed the proportion of respondents who received comprehensive counselling according to FP2020 criteria, those who were satisfied with services and who were poor according to the Equity Tool. Participants were classified as having received comprehensive counselling if they responded in the affirmative for at least 12 of the 15 questions, satisfied with received services if they responded in the affirmative for at least nine of the ten survey questions and poor if they were from the lowermost two wealth quintiles.

**Results**

Interviewees had a mean age of 27.9 (±6.5SD) years, 6.6% were aged 15-19 years, 27.9% were aged 20-24 years, and the remaining were 25+ years. About 64% had completed pre or primary education. Overall, 88% of respondents reported receiving high-quality counselling and 91.5% were satisfied with the services based on the set criteria. About 36% of the participants were categorized as poor. Respondents who were counselled according to FP2020 criteria were almost 15 times more likely to be satisfied with services than those who were not (OR 14.9, 95% CI 8.77-25.47; p<0.0001) and one’s satisfaction was not associated with the level of poverty (OR 0.81, 95% CI 0.50-1.30; p<0.40).

**Conclusions**

Comprehensive client counselling ought to be prioritized in this and similar settings for client satisfaction with available services.

## A75 Lesson on engagement of CHWS for early warning and rapid response in Buhingwe district, Kigoma

### Rita Mutayoba, Lusungu Ngailo, Frida Ngalesoni, Aisa Muya

#### Amref Health Africa in Tanzania, Dar es salaam, Tanzania

##### **Correspondence:** Rita Mutayoba (Rita.Mutayoba@amref.org)

**Background**

Emerging and re-emerging events with a potential to cause disease outbreak remain a constant threat to health security globally. The International Health Regulations, (IHR2005), legally binds WHO member states to work for global security though development of national public health capacities for surveillance and response to diseases. Tanzania remains under heavy threat from outbreak prone infectious diseases and non-communicable diseases, neglected tropical diseases and diseases targeted for eradication, (TDHS-MIS 2015/16). Amref, through CDC funding, supports MOHCDGEC to strengthen event-based surveillance (EBS) for early detection and response of PHEICS in Kigoma region.

**Approach**

Collaborating with regional and council teams, 116 CHWs recruited and trained using national EBS package followed by intensive continual capacity strengthening through supportive supervision, education forum and routinely by CHWs supervisors. CHWs are equipped with working gears including reference manuals, data collection tools, backpacks and umbrellas and allocated a hamlet that has up to 50 families. CHWs detect alerts using broad unstructured scenarios → immediately notify the health facility within 24 hours → support alert verification and risk assessment conducted by health care workers → verified alerts, events, call upon a response locally or may need support from other levels.

**Results**

In May 2021, CHWs from Songambele village notified the dispensary of one diarrhea event in the community. Within three hours, other CHWs reported 8 cases reaching 21 by end of day. This called for notification to council level who mobilized and deployed resources (personnel and medical supplies) to the event place. From a single case reported by CHW, 300 cases were identified and treated in 7 days and the diarrhea outbreak was contained within a small catchment area.

**Conclusion**

EBS is a vital component to early warning and rapid response for public health events and a scale up to other regions should be considered.

## A76 Using community own solutions to economically empowerment for fistula survivors

### Rita Mutayoba, MagdalenaDhalla, Frida Ngalesoni, Aisa Muya

#### Amref Health Africa in Tanzania, Dar es salaam, Tanzania

##### **Correspondence:** Rita Mutayoba (Rita.Mutayoba@amref.org)

**Background**

With limited access to quality maternal health services, obstetric fistula is a leading cause of pregnancy-related disability and stigma for around one million women across developing countries. Estimates show that 500,000 women and girls live with the condition in developing world . Obstetric Fistula, an abnormal opening between a woman’s genital tract and her urinary tract or rectum leaving women with life shattering consequences including chronic incontinence, shame, social isolation, poverty, physical, mental and emotional problems which, make it difficult to maintain sources of income, thus deepening poverty and magnifying suffering. Amref, works in Mwanza region to economically empower these women (fistula survivors) to regain social wellbeing.

**Approach**

Targeting 150 fistula survivors, a holistic approach used to empower these women economically. First, identification and treatment of fistula survivors in community through trained Fistula Ambassadors and then linked to Bugando Medical Centre (BMC) for surgical repair. Fistula survivors receive Psychosocial Counselling and Support (PSS) by trained Fistula Ambassadors, district social workers and members of community-based organization. Fistula survivors receive 3 days Entrepreneurship skills training and provision of seed fund for income generating activities (IGA) using tailor-made package. Fistula survivors propose type of IGA that Amref provides a seed fund.

**Results**

Between June 2019 and May 2021, 114 Fistula survivors were identified with 88 leaking urine or fecal linked to BMC repair, 26 treated through previous projects directly recruited for PSS. By May 2021, 50 fistula survivors received entrepreneurship skills training and provided with seed funds. Most preferred IGA is animal husbandry (52%), kiosk (30%) and cash crop selling (18%). Preliminary results show 30% contribute to their family income.

**Lessons Learnt**

Suffering from a devastating condition, Fistula survivors upon their surgical repair need support to regain their social wellbeing and become contributory family members.

## A77 Successfully index testing services. Experiences from Amref Afya Kamilifu supported sites Tanga and Zanzibar

### Joseph Kundy, Juma Dyegula, Noah Mwakisonga, Fulgence Ngeze, Nunuu Faki, Andrew Kapaya, Georgia Kasori, Erick Mboggo, Kilimba Edwin, Mponzi Victor, Muya Aisa

#### Amref Health Africa in Tanzania, Dar es Salaam, Tanzania

##### **Correspondence:** Joseph Kundy (joseph.kundy@amref.org)

**Background**

Amref Health Africa with support from CDC through Afya Kamilifu program is supporting Ministry of Health, Community Development, Gender, Elder and children and Ministry of Health Zanzibar to implement HIV services in Tanga and Zanzibar since October 2018 with the aim of driving the country towards the 95-95-95 HIV epidemic control. Prior to the program Tanga and Zanzibar, HIV testing yield, index testing yield and index testing positive contribution was low at 1.5%, 15% and 4% respectively. The program has for the last three years implemented innovative index testing maximization to improve testing efficiency across the supported sites.

**Methods**

The index testing maximization was implemented in 40 high volume sites across Tanga and Zanzibar starting July 2019. The approach mainly focuses on eliciting contacts, tracing and testing within 10 days of elicitation. Priority of index clients for elicitation includes newly diagnosed clients and clients with high viral load.

**Results**

Between July 2019 and June 2021, the project managed to improve overall HTS yield to 8.2 %, index testing yield to 29 % and index POS contribution to 66%. Also contacts tested and identified positive improved from 2043 and 191 end of March 2019 to 9906 and 2260 March 2021.

**Conclusions**

Index testing maximization approach successfully improved HIV testing efficiency and contributed to targets achievement after been scaled to sites. This approach is recommended to address gaps along the index testing care cascade to improve HTS efficiency.

## A78 Sanitation is dignity

### Simon Chiwanga

#### Amref Health Africa in Tanzania, Dar es salaam, Tanzania

##### **Correspondence:** Simon Chiwanga (simon.chiwanga@amref.org)

**Background**

Water and sanitation facilities are essential for human life, their improvement is also related to human respect and dignity. However, still 2 billion people do not have basic sanitation facilities according to WHO. WASH Standards in National Education Policy (2007) indicates pupils’ drop hole ratio to be one drop hole for 20 girls and one for 25 boys, a room for girls’ menstrual hygiene and special toilets for pupils with disability. Under UNICEF support, Amref assessed level of sanitation coverage at households and primary schools in implementation of a two-year project in Chunya District through sanitation marketing to improve sanitation and hygiene facilities, both in schools and at household level.

**Approach**

A mixed-study approach with quantitative and qualitative methods was used. The quantitative approach used a cross-sectional for school and household survey interviewing pupils and household heads. The qualitative method used key informant interviews with a structured checklist interviewing head teachers in selected schools, artisans, entrepreneurs and financial institutions in 10 villages. 10 wards were randomly selected from 20 wards of Chunya District, from which one school was selected from each ward. Analysis was conducted through STATA and NVivo software.

**Results**

In 9 schools visited, 44% of them don’t qualify with improved toilets; only 33% of girls’ toilets meet the national WASH standards in terms of drop hole ratio and no any single room for menstrual hygiene management which informs unhealthy environment for pupils especially girls, concluding indignity.

**Conclusion**

Amref will conduct a massive WASH campaign in Chunya to rehabilitate 48 school toilets including disabled and menstrual hygiene rooms, and handwashing facilities; educating 62 School WASH Clubs on water, sanitation and hygiene for all pupils to become community behaviour change agents. School sports and essay competitions will be conducted in propagating community health messages.

## A79 Tracking progress in rational medicine use - an assessment of key indicators in Dodoma region

### Eva Ombaka^1^, Jeremiah Seni^2^, Siana Mapunjo^3^, Sia Tesha^4^, Fiona Chilunda^4^, Karin Wiedenmayer^5^

#### ^1^Saint John’s University of Tanzania, Dodoma, Tanzania; ^2^Catholic University of Health and Allied Sciences, Mwanza, Tanzania; ^3^Ministry of Health, Community Development, Gender, Elderly and Children, Dodoma, Tanzania; ^4^Health Promotion and System Strengthening Project, Dodoma, Tanzania; ^5^Swiss Tropical and Public Health Institute, University of Basel, Basel, Switzerland

##### **Correspondence:** Eva Ombaka (e.ombaka@gmail.com)

**Background**

Health care systems cannot operate without medicines. Stock-outs of medicines in Tanzania are common and efforts focus on strengthening supply chain and availability of medicines at point of care. However appropriate use of medicines will ultimately determine quality of health care. Irrational prescription of medicines is harmful to patients and leads to waste of limited resources. This study tracks medicines use over time and explores the impact of interventions.

**Methodology**

A cross-sectional baseline study was conducted in 6 districts of Dodoma Region Tanzania in 2012 using WHO methodology. Subsequently, interventions to promote rational medicine use were implemented focusing on Standard Treatment Guidelines and antibiotic use. A repeat study in 2021 using the same WHO standard questionnaires included a random sample of 132 health care facilities. Data collected was analysed using STATA version 13.0.

**Results**

The number of prescribed medicines per patient encounter slightly increased from 1.9 in 2012 to 2.2 in 2021, while prescription conforming to the National Essential Medicine List (NEDLIT) declined from 98% to 91.2%. Generic prescription decreased from 97% to 63.9%, p-value <0.001. Antibiotic prescription increased from 66.0% to 80.9%, p-value <0.001. Injection use increased from 9.0% to 14.1%, p-value <0.001. Average consultation and dispensing time increased to 8.9 minutes and 105.7 seconds. Correct labelling of prescriptions remained unacceptably low at 2.2%. Patient knowledge about prescribed medicines decreased from 49.0% to 21.3% (p-value<0.001). Availability of reference material increased for STG/NEDLIT and Good Dispensing Manual. Access time to health care facilities by patients decreased.

**Conclusion**

The study showed improvement in availability of STG/NEDLIT, better accessibility to health facilities and patient care with longer provider-patient interactions. However, this does not translate into prescribing and dispensing practice, which has declined significantly for antibiotics and injections, generic prescribing, labelling of medicines and patient information. Findings show a disappointing trend of medicines use despite various interventions. Results should guide specific responsive actions at all levels. Quality of care cannot be improved unless limited resources are used more responsibly.

## A80 Drug dispensing outlets have high potential as source of community surveillance data

### Lusungu Ngailo, Rita Mutayoba, Aisa Muya, Frida Ngalesoni, Mwanakombo Khama

#### Amref Health Africa in Tanzania, Dar es salaam, Tanzania

##### **Correspondence:** Lusungu Ngailo (Lusungu.Ngailo@Amref.org)

**Background**

March 16th 2020, the Tanzania Ministry of Health Community Development, Gender, Elderly and Children (MOHCDGEC) announced the first case of corona virus disease 2019 (COVID-19). Immediately, the government instituted measures to respond and halt further transmission within the country. Drug dispensing outlets (Pharmacies and Accredited Drugs Dispensing Outlets (ADDO) were identified as one of the important sources of information that may reflect the magnitude of the diseases in the community. Collaborating with MUHAS, Amref Health Africa designed and piloted event based active surveillance (EBS) of COVID-19 cases among clients presenting with flu-like illness in ADDOs in Dar es Salaam to determine if drug dispensing outlets are potential source of information in the community for early and rapid detection of COVID-19 cases.

**Methodology**

103 ADDO near hospitals/health centers were conveniently sampled in Ilala and Kinondoni Municipalities. ADDO were opted to be piloted as part of EBS given the common habit of over-the-counter practices also being the first point of contact when people become sick to buy a drug of his/her choice or go to seek medical advice. A multi-faceted approach was implemented which included training of ADDOs and the surveillance team, collaborative continuous supportive supervision. First, screening using a standardized tool was done then alert notification from ADDOs were forward to Ward Health Officer or designated Rapid Response Teams (RRT) for further screening and linkages to health facilities.

**Results**

67% (69/103) of ADDOs reported an increased number of clients presenting with respiratory and flu-like illness in April 2020 as compared to the period before the outbreak. Common reported symptomatic complains by clients were cough (81%, 83/103), common cold (75%, 77/103) and fever (61%, 63/103). Between April 23rd and May 18th 2020, 75 alerts of COVID-19 suspect cases were recorded in ADDOs and linked to RRT. 17% (13/75) of alerts reported recent travel history from a foreign country while 4% (3/75) had a close contact with a COVID-19 confirmed case.

**Conclusion**

The pilot shows a promising strategy to expand EBS to include ADDO give their high willingness, collaboration and devotion. ADDO are potential to generate alerts of disease outbreaks in the community and hence timely response.

## A81 Pharmaceutical training and employment in the public sector: training alone is not enough

### Kelvin Msovela^1^, Romuald Mbwasi^1^, Sia Tesha^2^, Fiona Chilunda^2^, Karin Anne Wiedenmayer^3^

#### ^1^St John’s University of Tanzania, Dodoma, Tanzania; ^2^Health Promotion and System Strengthening Project, Dodoma, Tanzania; ^3^Swiss Tropical and Public Health Institute, University of Basel, Basel, Switzerland

##### **Correspondence:** Kelvin Msovela (kelvinmsovela@gmail.com)

**Background**

The estimated shortage of Human Resources for Health in Tanzania is about 50% and primarily affects the rural population. Shortage of health staff affects the quality of health service delivery, the attainment of UHC and health outcomes. Pharmacy staff shortage translates into gaps in pharmaceutical services, causing risks to patients. The pharmacy dispenser course is a 1-year vocational training with 23 weeks for theoretical training and 17 weeks for practical field work. This study explored the extent of applied learning back on the job and the impact on pharmacy practice.

**Methodology**

A comparative, post intervention assessment measuring indicators of pharmacy practice. 29 public health facilities employing pharmacy- trained dispensers (PTD) were compared with 32 health facilities without pharmacy-trained dispensers (NPTD) in Dodoma, Shinyanga and Morogoro regions. Course assessment results were included. Analysis was done using Microsoft Excel (2016) and SPSS (version 2.5).

**Results**

The average number of medicines per prescription was 2 and 84% of the prescriptions were filled. Documentation, handling of medicines and dispensing area cleanliness was slightly better for PTD as was medicines availability and stock record keeping. Good dispensing practice was low at 25% and without difference between PTD and NPTD. Dispensing time ranged from 1.8 – 2 minutes. There was no difference of patient knowledge on medicines. There was no difference for labelling and for storage practice between the two groups of dispensers.

**Conclusion**

The study showed no significant difference in performance of pharmacy practice despite a one-year training course which improves knowledge and skills and is highly valued by students, teachers and supervisors. Practice application not only depends on effective training but on the working environment. Clear job descriptions, tools, SOPs, acceptance by management, adapted work duties, personal attitude, recognition by team, and active encouragement to apply learnt skills are prerequisites. Training and knowledge alone does not lead to better practice and performance.

## A82 Improving diagnosis of childhood TB: preliminary results on fujilam and spk from “rapaed-TB”

### Alfred Mfinanga^1^, Harrieth Mwambola^1^, Laura Olbrich^2^, Heather Zar^3^, Issa Sabi^1^, Khosa Celso^4^, Denise Floripes^4^, Marriott Nliwasa^5^, Elizabeth Corbett^6^, Robina Semphere^5^, Joy Sarojini Michael^7^, Valsan Verghese^7^, Stephen Graham^8^, Rinn Song^9^, Pamela Nabeta^10^, Andre Trollip^10^, Christof Geldmacher^2^, Michael Hoelscher^2^, Norbert Heinrich^2^, Nyanda Ntinginya^1^

#### ^1^Mbeya Research Centre, National Institute for Medical Research, Mbaya, Tanzania; ^2^Ludwig Maximilian University, Munich, Germany; ^3^University of Cape Town Lung Institute, Cape Town, South Africa; ^4^Instituto Nacional de Saude, Maputo, Mozambique; ^5^Union Internationale de la Marionnette, Cherleville – Mezieres, France; ^6^London School of Hygiene and Tropical Medicine, London, United Kingdom; ^7^CMC; ^8^UNIMELB; ^9^German Centre for Infectious Research, Partner Site, Munich, Germany; ^10^Foundation for Innovative New Diagnosis, Geneva, Switzerland

##### **Correspondence:** Alfred Mfinanga (amfinanga@nimr-mmrc.org)

**Background**

The diagnosis of tuberculosis (TB) in children remains challenging: current detection methods neither perform reliably nor are sampling methods child-friendly.

**Methods**

RaPaed-TB is a diagnostic validation study currently conducted in South Africa, Mozambique, Malawi, Tanzania, and India. Enrolment of children ≤14years was initiated in January 2019. Clinical and laboratory workup is standardized across sites, and diagnostic classification follows the current NIH-consensus statement. New tests conducted on site include the urine-based lateral-flow assay Fuji SILVAMP-TB LAM (FujiLAM) and Stool Processing Kit (SPK) for MTB-DNA detection.

**Results**

As of April 2021, 846 participants were enrolled. The median age was 5.7years (1.8-8.8years) with 14% of children being <1year (117/846) and 37% <5years (312/846).Overall Microbiological confirmation rate (PCR/culture) was 21% (182/846).For AlereLAM, the overall sensitivity was 14.5% (95%CI 9.6-20.6) and specificity 92.9% (95%CI 88.0-96.3),while the sensitivity for FujiLAM was 34.9% (95%CI 27.8-42.6) and specificity 87.8% (95%CI 81.8-92.4).In our cohort, the SPK had a sensitivity of 35.8% (95%CI 28.5-43.6) and specificity of 87.9% (95%CI 81.9-92.4).When applying FujiLAM and SPK jointly, the sensitivity was 52.8% (95%CI 44.8-60.7) and specificity 73.6% (95%CI 65.8-80.5).The number of HIV-infected children with confirmed TB was small (16/182); no substantial difference in test performance was seen in this group. Further analysis of collected data is ongoing and therefore updated results will be presented during the forum.

**Conclusion**

The RaPaed-TB cohort allows large-scale evaluation of new tests. To our knowledge this is one of the first studies to prospectively evaluate FujiLAM and SPK head-to-head. Presented data on tests using easy-to-obtain samples indicate a modest performance of FujiLAM and SPK while showing better performance in some age subgroups. Additionally, FujiLAM performed better than AlereLAM with both the new tests indicating an improved performance in the very young and malnourished, showing their potential to aid diagnosis in these particularly vulnerable groups.

## A83 The in vitro activity of solanum carolinense extract against fungi isolated from skin infection of out patient

### Godlove Chaula, Emmanuel Sichone, Lwitiho Sudi, Jonathan Munkai, Nyanda Elias, Bariki Mtafya, Daniel Mapamba, Wolfram Mwalongo

#### Mbeya Research Centre, National Institute for Medical Research, Mbeya, Tanzania

##### **Correspondence:** Godlove Chaula (gchaula@nimr-mmrc.org)

**Background**

Fungal infections are occurring particularly in patients at high risk, immunosuppressed patients. Absidia corymbifera is found in family Cunninghamellaceae that can causes zygomycosis in the form of mycotic and also cause mucormycosis in humans. The in vitro antifungal activity of Solanum carolinense fruit extract has glycoalkaloids and was tested against isolated fungi.

**Methodology**

A case study was conducted at NIMR-Mbeya Medical Research Center, based on experiment, three samples from out patients were collected, from head, lap area and pus from wound, two samples by scraping and one sample by swabbing( pus discharge), were transported to the laboratory. Extraction was done from Solanum carolinense fruits fluid by squeezing and centrifugation, 100 to 102 were made. Samples were analyzed by methylene blue (celotape technique) and culture by Sabouraud dextrose agar. The pure cultures were adjusted to Mc Farland standard 1 using 0.85 % normal saline. Fluconazole was used as a positive control. The samples were analyzed using Microsoft Excel and recorded in figures.

**Results**

The macroscopic characteristics were, rapidly growth, white to greyish with, woolly texture cotton at 37oC, microscopic morphological characteristics were aseptate hyphae, resembles Rhizopus, sporangiophores arise on the stolon lie between the rhizoids, spherical sporangia to pyriform in shape, Absidia corymbifera was isolated.

**Conclusion**

Solanum carolinense fruit extract has greater antifungal activity against Absidia corymbifera and not only this specie can be found on skin but also in infected wounds.

## A84 Pathogenic fungi from bat droppings causing histoplasmosis in human in southern west of Tanzania: Mbeya region

### Godlove Chaula, Emmanuel Sichone, Lwitiho Sudi, Jonathan Munkai, Nyanda Elias, Bariki Mtafya, Daniel Mapamba, Wolfram Mwalongo

#### ^1^ Mbeya Research Centre, National Institute for Medical Research, Mbeya, Tanzania

##### **Correspondence:** Godlove Chaula (gchaula@nimr-mmrc.org)

**Background**

Histoplasma is a genus of dimorphic fungi commonly found in birds and bats fecal materials and Histoplasma capsulatum is the causative agent of histoplasmosis which occurs worldwide and should not be overlooked in patients with unexplained pulmonary or systemic illnesses. H. capsulatum has been reported to cause human disease in the coastal areas around the cities of Tanga and Dar es Salaam. After exposure chronic histoplasmosis can resemble tuberculosis.

**Methodology**

This was a case study conducted at NIMR-MMRC, bats droppings were collected into falcon tubes and transported to the lab prior processed, 0.5ml of 0.85%Nacl was used to prepare the inoculum by votexing, few drops were inoculated on two Sabouraud dextrose agar plates, we incubated one plate at room temperature for few days and another at 370c for 24hours. The results were identified macroscopically and microscopically.

**Results**

Culture at 37°C we observed 2-3mm, wrinkled, moist, heaped and creamy yeast like colonies, Gran stain we observed round, oval budding yeast cells at room temperature we observed white, fluffy mold that turns to brown to buff with age in celotape techniques we observed the mycelium with round microconidia.

**Conclusion**

From the results the fungi investigated was Histoplasma capsulatum which is the causative agent of Histoplasmosis in human and people should be prevented from exposed spores. The chronic stage of histoplasmosis have similar symptoms like those people who have M. tuberculosis.

## A85 Use of data for improving access and delivery of healthcare: a call for capacity strengthening at PHC level

### Paul E Kazyoba^1^, Prince Mutalemwa^1^, Justin Omolo^1^, William Reuben^2^

#### ^1^National Institute for Medical Research, Dar es salaam, Tanzania; ^2^President’s Office Regional Administration and Local Governments, Dodoma, Tanzania

##### **Correspondence:** Paul E Kazyoba (paul.kazyoba@nimr.or.tz)

**Background**

Availability of medical commodities is an important aspect for service delivery and responsive health system. Thus Interrogating systemic gaps which affect access to medicine at the primary healthcare (PHC) is an important undertaking to enable timely intervention.The Access and Delivery Partnership has been working towards understanding gaps which contribute to persistent stock outs of tracer medicines at the PHC with the aim to devise interventions to address them.

**Objective**

To strengthen capacity of the PHC to mine and utilize data accurately during quantification of medical commodities.

**Methodology**

This was a cross sectional study involving three key steps of implementation research namely; pre-intervention, intervention and evaluation of the intervention. The implementation of activities was inclusive and involvedDistrict Pharmacists, Regional Medical Officers, Council Medical Officers, Regional Pharmacists and medical officers in charge from Health centers, District Laboratory Technologists (DLT) and Council HMIS Coordinators. The MoHCDGEC through PSU and the PORALG through the Department of health services, social welfare and nutrition services.

**Results**

Working through the health system building blocks, fifty two (52) implementation and/or operational gaps were identified and prioritized. Out of these 29 (55.8%) were ranked as higher priority gaps needing urgent intervention, while 44.2% of the gaps were of medium and low priority implying they may need intervention at a later stage. Among key gaps which were identified as drivers for drug stock outs at PHC level include limited skills on the use of health information systems. For instance, lack or inadequate orientation of healthcare workers on the use of health information systems (HIS), limited on job training on supply chain management and utilization of the health information systems, etc. Based on the gaps analysis and limited resources, providing training to key personnel from the PHC on gaps related to data mining and use will address several others gaps linked to drug stock outs.

**Conclusion**

There is a need to provide regular training on technical personnel at the PHC level to be able to mine and use data during planning and decision making. With respect to access to medicines, the DLTs, DPs and Council HMIS coordinators need regular training on data mining and use, and accurate posting of routine data.

## A86 Transmission and spread of STIs and RTIs among youths’ in higher learning institutions and neighbor areas: the case of Mbeya city in Tanzania

### Leoncia H Kibani, Agustino Matem, Furaha Mwangosi

#### Mbeya University of Science and Technology, Mbeya, Tanzania

##### **Correspondence:** Leoncia H Kibani (lkibani@yahoo.com)

**Background**

Love affairs and imitated modernization habits among youthsare resilient contributors of sexually risk behaviours and prevalence of attack by sexually transmitted infections (STIs/RTIs/HIV/AIDS). However, paucity studies have dealt with sexual transmitted infections to youths in higher learning institutions.

**Objective**

Therefore, this study aims to investigate on the transmission and spread of STIs, RTIs and HIV/AIDS among youths’ in higher learning institutions and neighbor villages.

**Methodology**

The study was conducted in three higher learning institutions and three villages in Mbeya City District. A total of 210 students and 100villages youths all aged 19-30 years were randomly sampled for the study. A cross-sectional design was adopted. Thematic and descriptive statistical analysis was employed.

**Results**

The results showed that youths aged between 19-30 years engaged much in love affairs with integrated modern cultures but limited understanding on possible risk outcomes. Findings showed that 82% females and 23% male infected with Chlamydia and Gonorrhoea did not showed symptoms.71% youths aged 19-26 are more infected with STIs/RTIs/HIV than 54% who are 27-30 years. Majority 83% village youthsand 73% university students had limited knowledge on STIs/RTIsinfections. Villages around universities had more young females with single parent children who’s further were unknown university students.Village male youths were in conflict with university male students because of love affairs with females. Alsofrom January-June 2021, students with Gonorrhea in institution ‘A’ were 5% male and 3% female; Institution ‘B’ 7% male and 4% female; Institution ‘C’were 5% male and 4% female.

**Conclusion**

These findings indicate that love affairs in higher learning institutions and neighbor are contributing factors ofsexual transmitted infections. This indicates that in the coming years there will be more STIs/RTIs/HIV/AIDS and children. Therefore, it is imperative to establish sensitization programs for educating about STIs/RTIs/HIV/AIDs and resulted risks.

## A87 Strengthening Tanzania’s pandemic preparedness and response capacities

### Kate Molesworth^1^, Ally-Kebby Abdallah^2^

#### ^1^Swiss Tropical and Public Health Institute, University of Basel, Basel, Switzerland; ^2^Health Promotion and System Strengthening, Dodoma, Tanzania

##### **Correspondence:** Kate Molesworth (karin.wiedenmayer@swisstph.ch)

**Background**

Throughout the life of the project, the Health Promotion and System Strengthening (HPSS) Tuimarishe Afya Project funded by the Swiss Agency for Development and Cooperation (SDC) and implemented by the Swiss Tropical and Public Health Institute (Swiss TPH), has provided the Government of Tanzania with rapid support to disease outbreaks and pandemic responses. This included the 2015 cholera outbreak, the 2016 outbreak of acute liver disease resulting from ingesting aflatoxin, the HIV response and more recently, the Coronavirus disease 2019 (COVID-19) pandemic response.

**Methodology**

Since the first case of COVID-19 infection in Tanzania was reported on 17th March 2020. HPSS, together with other partners such as UNICEF, supported the Government of Tanzania to implement of its COVID-19 Emergency Response Plan, contributed to the national COVID-19 Emergency Preparedness Task Force, and has been actively engaged in the Risk Communication and Community Engagement Pillar of the Emergency Response Plan.

**Results**

To address the gap in public information, the project printed and distributed 100,000 posters country-wide, supported radio spots produced by the government both on national and on local community radio stations. To address public and health authorities’ concerns and lack of knowledge, to contain misinformation, and provide guidance on testing, care and treatment, HPSS jointly with UNICEF, IMA World Health and UNFPA supported the enhancement and capacity of the existing health hotline to provide the national “Afya Call Centre” for the Corona virus hotline “199”, launched in May 2020. The IT system can accommodate 500 concurrent calls and has the possibility to be connected with stations in other cities across. It operates 24 hours a day with two shifts of 40 operators each.

**Conclusion**

The new call centre substantially increases the capacities of the Ministry of Health to respond to disease outbreaks and pandemics both now and into the future.

## A88 Nurses’ use of CPOT on patients unable to self-report pain in the ICUs of the national hospital in Tanzania

### Zainab K Manji, Beatrice Mwilike, Agnes Massae

#### Muhimbili University of Health and Allied Sciences, Dar es salaam, Tanzania

##### **Correspondence:** Zainab K Manji (zainab.karim4@gmail.com)

**Background**

Self-reporting is the most reliable indicator of pain, however it is not possible to achieve in critically ill patients. Therefore, it is important to have a valid, reliable, and accurate pain assessment tool for assessment of pain in such patients. The Critical Care Pain Observation Tool (CPOT) has been recommended as the most valid and reliable by various critical care organizations.

**Objective**

To assess nurses’ knowledge and perception on feasibility of the CPOT among patients unable to self-report pain in the ICU of the National Hospital.

**Methodology**

Single-group pretest posttest study involving 111 nurses working in the ICU’s of the National Hospital. Two questionnaires were administered to the participants, pre and post intervention, the CPOT was introduced and its correct usage taught in form of a training as an intervention. Data was analyzed using the SPSS 25.0 software with the help of descriptive statistics as well as inferential statistics.

**Results**

100% of the nurses were aware of the importance of assessing pain for patients unable to self-report and thought it was important to have a standard tool for doing so. However, only 20% of the nurses had previously heard about the CPOT and just 50% of them had used it on their patients, majority of who (63.6%) had inadequate knowledge on its appropriate use. There was significant improvement in their knowledge after training (p value 0.001). Nurses agreed that the CPOT is a feasible tool for use in their current setting.

**Conclusion**

Majority of the nurses didn’t know of the existence of the CPOT and relied on physiological parameters to assess pain, despite knowing the importance of assessing pain and having a standard pain assessment tool for patients unable to self-report. After the tool was introduced to the nurses, their knowledge on its appropriate usage was adequate and they perceived it as a feasible tool. Continuous professional education on pain assessment for patients who cannot self-report is required across all ICU’s for nurses and also needs to be incorporated into nursing curriculums at university. Follow up studies are required to assess nurses implementation of the CPOT. Recommendations can then be made to create standard pain assessment policies.

## A89 The prevalence of sexually transmitted infections and risk factors among young adult female, Mbeya-Tanzania

### Anifrid Mahenge^1^, Wolfram Mwalongo^1^, Jonathani mnkai^1^, Regino Mgaya^1^, Willyhelmina Olomi^1^, Ruby Mcharo^1^, Beatrice Komba^1^, Christof Geldmacher^2^

#### ^1^Mbeya Research Centre, National Institute for Medical Research, Mbeya, Tanzania; ^2^Ludwig Maximilian University, Munich, Germany

##### **Correspondence:** Anifrid Mahenge (amahenge@nimr-mmrc.org)

**Background**

Sexually Transmitted Infections (STIs) are currently the global public health problem with serious threat to the infected individuals, either with symptoms or asymptomatic. Most of these curable STIs are treated syndromically and untreated cases cause complications in pregnancy and perinatal sequelae,chronic pelvic diseases and increased risk of HIV acquisition. This was cross sectional study which investigated the prevalence and risk factors of 7-STIs from young adults females.

**Methods**

This study was conducted since April 2020 whereby total of 170 bio-banked samples at NIMR-MMRC were randomly selected, then were collected from young adult women from various universities in Mbeya region. Of which 150 cytobrush and 20 urine samples were extracted using Qaigen kit and detected with 7-essential STIs assay using multiplex CFX96 system. Data were analyzed using Stata 14 and graph pad prism software.

**Results**

The overall prevalence of STIs is 81.33% of which (MG/CT/NG/TV 19.33% are public health important pathogens. The specific prevalence to each pathogens were Ureaplasma parvum(56%),Microplasma hominis and Ureaplsma urealyticum (38%), Chlamydia trachomatis (14.67%), Microplasma genitalium (6.67%),Trichomonas vaginalis (1.33%) Neisseria gonorrhea (0.67%). The proportion of STIs co-infection with Human Papillomaviruses(HPV) was (28.67%) and great correlation of CT from multiple infections observed. Also, 93% of women who were diagnosed with STIs pathogen(s), never had sex and diagnosed with either 7-STIs. More than 50% of females who were diagnosed with STIs pathogens they had no obvious STIs symptoms. As well as 78% of all females had poor knowledge of STIs of which 52.67% first year university females and 66.67% non-residence. Furthermore, seegene multiplex is superior in STIs diagnosis using cytobrush with the sensitivity and specificity of 83.33% and 92.87% against CT/NG geneXpert.

**Conclusion**

This study demonstrated high prevalence of STIs among young adult females who are vulnerable to STIs. The crude risk for STIs are gender, sex experience, age, knowledge and residence. Most of STIs are asymptomatic with multiple infections; molecular diagnosis of STIs offers reliable and accurate results. Thus, cytobrush specimen is superior in STIs diagnosis compared to urine. Further research is needed to report STIs pathogen.

## A90 Controlling post partum haemorrhage using mkanda salama in Tanzania health facilities

### James Kalema^1, 2^, Elias Kweyamba^2^, Esther Kyungu^2^

#### ^1^Tanzanian Training Center for International health - Ifakara, Morogoro, Tanzania; ^2^Muhimbili university of health and allied sciences, Dar es salaam, Tanzania

##### **Correspondence:** James Kalema (kalemajames30@gmail.com)

**Background**

Based on Tanzania administrative data and policy Implication report of 2018, revealed that PPH contributed to about 29% of all maternal deaths in Tanzania. MKANDA SALAMA KIT is the new innovated technological device designed to stop PPH.

**Objective**

To determine the effects of MKANDA SALAMA KIT (MSK) on the clinical symptoms improvement, hemodynamic restoration, and bleeding stoppage in post-delivery mothers with Postpartum hemorrhage (PPH). The primary outcome was the composite of PPH maternal death and PPH rehospitalization within42 days follow-up time; secondary outcomes were all causes of maternal death and the occurrence of rehospitalization for worsening PPH.

**Methods**

Data from 120 women with PPH aged from 13years to 39 years from December 2020 to May 2021 (6 months) at St. Francis referral hospital labor ward were included. Patients were grouped into mild PPH, moderate PPH, and severe PPH. A multinomial regression model was used to determine the associations between risk and outcomes using SPSS software version 21.0˝ (Chicago, Illinois, USA).

**Results**

Twenty-three percent (23%) had a previous history of PPH, 36% had maternal hypertension, 45% had anemia, and 12% had maternal diabetes. Mild PPH was 20%, moderate PPH was 37%, and severe PPH was 43%. MKANDA SALAMA KIT significantly improved clinical symptoms and signs of all PPH categories by 84%. A significant restoration to normal all hemodynamic parameters was observed to all patients by 79% MKANDA SALAMA KIT significantly stopped mild PPH by 100% within 10-20 minutes, while stopped moderate PPH by 70.3% within 10-20 minutes and stopped severe PPH by 63.5% within 10-20 minutes. There was no rehospitalization during follow-up time and no death was reported.

**Conclusion**

MKANDA SALAMA KIT significantly improved clinical symptoms, hemodynamic parameters and stopped bleeding in PPH patients, timely application of MSK can significantly prevent PPH maternal death and PPH rehospitalization.

## A91 Traditional medicine practices in Sub-Saharan Africa. A case study of coronavirus (covid-19) management

### Ndahani Msigwa, Marko Hingi

#### Traditional and Alternative Health Practice Council, Dar es salaam, Tanzania

##### **Correspondence:** Ndahani Msigwa (markohingi@gmail.com)

**Introduction**

Coronavirus (COVID-19) continue to emerge and represent a serious threat to public health across the World. The +stranded RNA viruses with a crown-appearance have ranged from asymptomatic to severe illness. Tanzania has reported 509 cases and the virus have claimed lives of 21 patients.

In fight of COVID-19 crisis in Tanzania, various measures were taken including social distance, putting mask, hand hygiene as well as traditional methods such as herbal remedy and steam inhalation administration.

**Methods and Analysis**

In traditional medicine practices, COVID-19 infection was regarded as cold related illness and Traditional Health Practitioners encouraged patients to take herbal remedysuch as mixture of ginger, onion, cayenne pepper, garlic and lemon which can warm their body and remove dumpnessin the lungs alternatively they administered steam inhalation from plants such as Ocimum Suave, Lemon grass and Eucalyptus leaves. All these methods relieved symptoms of COVID-19 and prevented further deterioration of the COVID-19 patients.

14 samples of herbal remedy were taken for chemical (phytochemical) analysis. The analysis results were as follows Flavonoids (87.5%), Saponins (35.7%), Alkaloids (35.7%), Capsians (14.2%), Steroids (7.1%), Eucalyptol (7.1%) and Anthraquinones (7.1%)

**Discussion**

The literature retrieves that the flavonoids have potential inhibitory and immunomodulatory activities against severe acute respiratory syndrome coronavirus 2 (SARS-CoV-2) designated as COVID-19 also has showed excellent anti-inflammatory activities including the inhibition of various inflammatory cytokines. Further, flavonoids showed significant ability to reduce the exacerbation of COVID-19 in the case of obesity through promoting lipids metabolism.

**Conclusion**

There is gap in translating the traditional medicines practices and knowledge into conventional practices due to inadequate information regarding phytochemical perspective of the medicinal plants used by Traditional health practitioners in Tanzania and Africa.

## A92 The patterns and cause-specific in-hospital mortality among older children and adolescents in Tanzania, 2006-2015

### Leonard E Mboera^1^, Susan F. Rumisha^2,3^, Veneranda M Bwana^4^

#### ^1^SACIDS Foundation for One Health, Sokoine University of Agriculture, Morogoro, Tanzania; ^2^National Institute for Medical Research, Headquarters, Dar es Salaam, Tanzania; ^3^Malaria Atlas Project, Geospatial Health and Development, Telethon Kids Institute, West Perth, Australia; ^4^Amani Research Centre, National Institute for Medical Research, Tanga, Tanzania

##### **Correspondence:** Leonard E Mboera (vbwana@gmail.com)

**Background**

Despite the available statistics, little is known on the causes, trends and pattern of mortality among older children (5–9 years) and adolescents (10–19 years). This retrospective study was carried out to determine the pattern and cause-specific in-hospital mortality among older children and adolescents in Tanzania.

**Methods**

A multistage sampling technique was employed to select a representative number of hospitals from regions and districts in Tanzania. Analysis of in-hospital mortality from 2006 to 2015 was performed to identify leading cause of deaths, geographical, temporal and demographic variations. Age-standardized hospital mortality rates per 100,000 were calculated. A Bayesian spatial logistic regression model was used to estimate the spatial patterns and probability of dying from leading causes of death.

**Results**

A total of 247,976 deaths were reported from 39 hospitals during the 10-year period. Of these, 17,898 (7.2%; 95% CI: 7.1-7.4) affected the 5-19-year-old individuals. The 5-9, 10-14 and 15-19 years accounted for 42.5% (95% CI: 41.2-43.8), 26.8% (95% CI: 25.6-28.0) and 30.7% (95% CI: 29.5-31.9) of the deaths, respectively. The overall age-standardized mortality rates for 2006-2010 and 2011-2015, were 187.4 and 329.4 deaths per 100,000 population, respectively. The five major specific causes of death in older children and adolescents were malaria (28.8%), anaemia (16.9%), respiratory diseases (5.7%), injury (5.1%), and meningitis (3.9%). The geographical distribution of the probability of dying from the top causes varied by cause of death and geographical region.

**Conclusion**

In Tanzania, mortality among older children and adolescents contribute to about 7.2% of the total hospital deaths. There are significant variations on major causes of death between sex and age as well as geographical regions. These findings call for strategic multisectoral public health responses to reduce deaths among older children and adolescents.

## A93 Parasite infectivity rate and blood meal sources of host seeking malaria vectors of north-eastern Tanzania

### Subam Kathet^1, 2^, Ayman Khattab^1^, Mwantumu Ally Ferei^2^, Gladness Kiyaya^2^, Victor Mwingira^2^, Seppo Meri^1,3^, William N. Kisinza^2^,

#### ^1^Research Program Unit, Translational Immunology Research Program, Haartman Institute, University of Helsinki, Helsinki, Finland; ^2^Amani Medical Research Centre, National Institute for Medical Research, Tanga, Tanzania; ^3^Helsinki University Central Hospital, Helsinki, Finland

##### **Correspondence:** Subam Kathet (subam.kathet@helsinki.fi)

**Background**

This study was conducted in Muheza district of North-East Tanzania as a part of baseline data collection accounting for a prospective cluster randomised trial to evaluate the efficacy of combining novel vector control tool with LLIN’s in reducing malaria vectors.

**Methods**

Adult mosquito samples were collected for 28 consecutive nights from 20 villages during May-June 2019 and November-December 2019 using a CDC miniature light traps installed in 5 different household from each village randomly selected every night. Collected samples were identified to species using morphological keys and their gonotrophic status were recorded. Engorged specimens were transferred separately for laboratory analysis followed by DNA extraction using CTAB method. Sibling species and blood meal sources were established using PCR and Plasmodium infection was validated using melt curve RT-PCR assay.

**Results**

A total of 1174 blood fed mosquito sample were collected during the long and short rainfall of 2019 of which 72.23% (n = 848) and 27.76% (n = 326) were identified morphologically as Anopheles funestus s.l. and An. gambiae s.l. respectively. Within An. funestus s.l. population, 93.75% (n = 796) were An. funestus s.s., 4.54% (n = 38) were An. leesoni and 1.70% (n = 14) were An. rivulorum while An. gambiae s.l. population comprised of 87.65% (n = 286) An. gambiae s.s. and 12.35% (n = 40) An. arabensis. From melt curve analysis, Plasmodium falciparum infections rates were reported to be 1.21% and 0.58% among blood-fed An. gambiae s.l. and Anopheles funestus s.l. respectively. Based on PCR analysis of vertebrate cytochrome b, humans (56.7 %) were the prominent blood-meal hosts of malaria vectors while 37.33% of blood-meals were from non-human vertebrate hosts.

**Conclusion**

Blood fed mosquito population was dominated by An. funestus s.l. however P. falciparum infection rate was significantly higher in An. gambiae s.l.

## A94 Community engagement around the implementation of trial of insecticide-treated wall lining for malaria control in rural Tanzania: lessons from cluster randomized trials in Tanzania

### Peter E Mangesho^1^, Mohamed S Mohamed^1,^, Ruth C Mnzava^1^, Kaseem J Mkuza^1^, William N Kisinza^1^, Joseph P Mugasa^1,4^, George Mtove^1^, Kihomo R Mpangala^2,3^, Donald S Shepard^2^, Yara A Halasa-Rappel^2,^ Louisa A Messenger^5^, Aggrey R Kihombo^6^,

#### ^1^ Amani Research Centre, National Institute for Medical Research, Tanga, Tanzania; ^2^Brandeis University; ^3^Empowered and Improvement Livelihood Initiatives Foundation, Dar es salaam, Tanzania; ^4^Population Services International, Dar es Salaam, Tanzania; ^5^London School of Tropical Medicine and Hygiene, London, United Kingdom; ^6^Mzumbe University, Morogoro, Tanzania

##### **Correspondence:** Peter E Mangesho (mseif19@gmail.com)

**Background**

Community engagement (CE) is gradually promoted for Bio-medical research conducted in resources poor settings. During community trials CE is a complex social phenomenon that defies simple explanation or mechanization employed to engage the community. However, there is scarce documented local experiences on CE in Bio-medical research in Tanzania.

**Objective**

To assess the sensitization process, experiences and challenges in improving understanding and subsequent acceptance of an insecticide treated durable wall lining project in rural Tanzania.

**Methodology**

Prior the project we conducted meetings with village leaders to introduce the project. Village leaders prepared community engagement meetings by using the traditional approach “Mbiu”to invite the villagers at the meetings places, whereas, the researchers in support of local leaders addressed the community about the trial.

**Results**

The meetings were poorly attended due long walking distance to the meeting locations, farming activities and presidential election campaigns. Sensitization was re-strategized to add door-to-door sensitization, announcements using a megaphone, designing and distribution of brochures detailing the study objectives and consenting process. The process continued during all three installation phases.

The new strategies rose an acceptance rate from 31.5% to 61.5%. However, some clusters still had some refusals. Reasons included gender and consent, For example, in some houses the head of house (generally a man) refused installation after the wife had accepted; Old rumors resurfaced that ITWL contributed to male impotence. Some installers, initially unprotected, developed skin rashes and the message reached all over. Fear of damaging house walls. Directives that children should not touch the wall liners and confusion from installation delay all fed into refusal rates.

**Conclusions**

Re-strategizing sensitization plus continuous sensitization throughout and after the official installation period increased ITWL acceptance. Future projects should not rely on a single sensitization approach and consider using specialized village research committees for improved CE.

## A95 Integrating HIV, diabetes and hypertension services in Africa: study protocol for a cluster-randomised trial in Tanzania and Uganda

### Sayoki Mfinanga^1,2^, Sokoine Kivuyo^1^, Samafilan Ainan^1^, Joseph Okebe^2^, Anupam Garrib^2^, Josephine Birungi ^3,4^, Godfather Kimaro^1^, Elizabeth Shayo ^1^, Kaushik Ramaiya^5^, Erik van Widenfelt^2^, Marie Claire van Hout ^6^, Max Bachmann ^6^, Dominic Bukenya^7^, Walter Cullen^2^, Jeffrey V Lazarus ^6^, Louis Niessen^2^, Anne Katahoire^7^, Ivan Namakoola^4^, Tinevimbo Shiri^2^, Duolao Wang^2^, Luis E Cuevas^2^, Bernard Etukoit^7^, Janet Lutale^8^, Shimwela Meshack^9^, Kenneth Mugisha^10^, Geoff Gill,^2^ Nelson K Sewankambo^7^ Peter G Smith^6^, Moffat Nyirenda, Shabbar Jaffar ^4,6^

#### ^1^National Institutes for Medical Research, Dar es Salaam, Tanzania; ^2^Liverpool School of Tropical Medicine, Liverpool, United Kingdom; ^3^The AIDS Support Organisation, Mulago Hospital Complex, Kampala, Uganda; ^4^MRC/UVRI & LSHTM Uganda Research Unit, Entebbe, Uganda; ^5^Hindu Mandal Hospital, Dar es Salaam, Tanzania; ^6^London School of Hygiene and Tropical Medicine, London, United Kingdom; ^7^Makerere University College of Health Sciences, Kampala, Uganda; ^8^Muhimbili University of Health and Allied Sciences, Dar es Salaam, Tanzania; ^9^Amana Regional Referral Hospital, Dar es Salaam, Tanzania; ^10^Non-Communicable Diseases Control Programme, Ministry of Health, Kampala, Uganda; ^11^Directors Office, Ministry of Health, Community Development, Gender, Elderly and Children, Dodoma, Tanzania

##### **Correspondence:** Sayoki Mfinanga (tmaya.tz@gmail.com)

**Background**

Disease epidemiology has changed rapidly in Africa. Until about a decade or so ago, African health services were dealing principally with acute infections. HIV programmes in sub Saharan Africa are well-funded and crucially over 60% of people with HIV-infection are in regular care with good viral suppression. The programmes for diabetes and hypertension are weak, and the burden of diabetes and hypertension is rising where only less than 5% of people with diabetes or hypertension are in regular care. This trial is evaluating a concept of integrated care for people with HIV-infection, diabetes or hypertension from a single point of care.

**Methods**

A total of 32 primary care health facilities in Dar es Salaam and Kampala regions were randomised to either integrated or standard vertical care in a 1:1 ratio. In integrated care, services are organised from a single clinic where patients with either HIV-infection, diabetes, hypertension or combinations of these are managed by the same clinical and counselling teams. They all use the same pharmacy and laboratory and they have the same style of patient records. Standard care involves separate clinics, waiting areas, counselling areas, pharmacies and medical records.

INTE-AFRICA will measure both efficacy data in patients, health economics data and aggregated data at the health facility level. The trial has 2 primary endpoints: retention in care of people with HIV, hypertension and diabetes and plasma viral load suppression. Recruitment is expected to take 3-months and follow-up is for 12 months.

With 100 participants enrolled in each facility with diabetes or hypertension, the trial will provide 90% power to detect an absolute difference in retention of 15% between the two study arms (at the 5% two-sided significance level). If 100 participants with HIV-infection are also enrolled in each facility, we will have 90% power to show non-inferiority in virological suppression between the 2 arms to a delta=10% margin.

**Discussion**

This is the only randomised trial of its kind evaluating a one-stop integrated clinic for common high-burden diseases in Africa, designed to generate policy-relevant evidence on the re-organisation of chronic care services in Africa. The identification of a sustainable and effective integration model could lead to a substantial improved health services for these chronic conditions in resource poor settings.

## A96 After-action review of rabies and anthrax outbreaks multi-sectoral response in Tanzania, challenges and lessons

### Kunda John^1, 2^, Justine Assenga^2,6^, Jubilate Bernard^2,3^, Ernest Eblate^5^, Elibariki Mwakapeje^3^, Janeth Mghambai^3^, Harrison Chinyuka^2^, Dominic Kambarage^4^

#### ^1^Muhimbili Medical Research Centre, National Institute for Medical Research, Dar es Salaam, Tanzania; ^2^One Health Coordination Desk, Prime Minister’s Office, Dodoma, Tanzania; ^3^Ministry of Health, Community Development, Gender, Elderly and Children, Dodoma, Tanzania; ^4^Sokoine University of Agriculture, Morogoro, Tanzania; ^5^Tanzania Wildlife Research Institute, Arusha, Tanzania; ^6^Ministry of Livestock and Fisheries, Dodoma, Tanzania

##### **Correspondence:** Kunda John (john.kunda@gmail.com)

**Background**

After-action review uses experiences gained from past events to adopt best practices, thereby improving future interventions.In December 2016 and late 2018, the government of Tanzania with support from partners responded to anthrax and rabies outbreaks in Arusha and Morogoro regions respectively. The One health coordination desk (OHCD) of the Prime Minister’s Office (PMO) later coordinated after-action reviews to review the multi-sectoral preparedness and response to the outbreaks.

**Objectives**

To establish and describe actions undertaken by the multi-sectoral investigation and response teams during planning and deployment, execution of field activities, and outbreak investigation and response, system best practices and deficiencies.

**Methodology**

These were ross-sectional surveys. Semi-structured, open and closed-ended questionnaire and focus group discussions were administered to collect information from responders at the national and subnational levels.

**Results**

It was found that the surveillance and response systems were weak at community level, lack of enforcement of public health laws including vaccination of livestock and domestic animals and joint preparedness efforts were generally undermined by differential disease surveillance capacities among sectors. Lack of resources in particular fundsfor supplies, transport and deployment of response teams contributed to many shortfalls

**Conclusion**

The findings underpin the importance of after-action reviews in identifying critical areas for improvement in multi-sectoral prevention and control of disease outbreaks. Main sectors under the coordination of the OHCD should include after action reviews in their plans and budget it as a tool to continuously assess and improve multi-sectoral preparedness and response to public health emergencies.

## A97 Accessibility and utilization of birth companions in public facilities: lessons from implementation of birth companionship in Kigoma and Katavi region

### Sunday Dominico, Alex Mputa, Agnes Mbanza

#### Thamini Uhai, Dar es salaam, Tanzania

##### **Correspondence:** Sunday Dominico (sdominico@thaminiuhai.or.tz)

**Introduction**

A birth companion is a non-medical person who provides emotional, physical and practical support to pregnant women during pregnancy, labor and childbirth^1^. During the scale-up phase(unlike the pilot phase), the on-call birth companions(OBCs) who were recruited, incentivized and stationed at the labour ward throughout to support needy women were dropped to retain only the cost-effective and sustainable desired birth companion (DBCs, women’s relative or friend, oriented at antenatal clinic, not paid incentives); a significant change from a model. The desired birth companions were oriented by facility-based maternity healthcare providers who were trained by the project on birth companionship including comfort measures.

**Methodology**

Scale-up phase covered four health centers in Katavi region from May 2019 to April 2021. Communities were sensitized and women delivering at intervention sites were allowed to have a birth companion during childbirth. Access to companions was documented monthly for 15 months (Jan 2020 – Mar 2021), by recording women who delivered with a birth companion, expressed as proportion of all deliveries. We compared birth companion’s utilization during pilot phase (a model with both on call and desired birth companions) and scale-up phase for Katavi region (with Desired Birth companions only), to see impact on birth companions utilization rates and recommendations for birth companionship in future deliveries. .

**Findings**

During 15 month Pilot phase in Kigoma, 82% of women had a companion while in scale-up phase in Katavi region, 92% of women had a companion during labor and delivery during 1 15month implementation period, a 10% increase. A gradual increase in companion’s utilization was noted from 84% in the first quarter to 95% in the last quarter. Nearly all women (99%) who responded to exit interview in both pilot phase(Kigoma) and scale-up phase(Katavi), said they would like to have a companion next time they deliver.

**Conclusion**

The change in the birth companionship implementation model, didn’t reduce accessibility and utilization of birth companionship and satisfaction rate remained high (nearly universal). Provider’s awareness, readiness and community engagement during implementation were vital for successful and sustained implementation.

## A98 Factors associated with successful introduction and sustainability of birth companionship intervention: lessons a qualitative evaluation of birth companionship in Katavi region, April 2021

### Sunday Dominico, Alex Mputa, Agnes Mbanza

#### Thamini Uhai, Dar es salaam, Tanzania

##### **Correspondence:** Sunday Dominico (sdominico@thaminiuhai.or.tz)

**Introduction**

Birth companionship was introduced as pilot intervention in 2016 by Thamini Uhai in Kigoma region. Since then, significant progress has been achieved towards being routine practice during labor and childbirth. In 2019, a national guideline was launched that allow implementation of birth companionship as a key component of respectful maternity care; and is recommended possible remedy to prevent mistreatment during childbirth, by having companions assume roles such as, witness, or safeguard for the woman they are supporting.

**Method:**

Thamini Uhai implemented a two-year project and evaluated what factors were associated with sustainability of birth companionship in Katavi region. A cross-sectional design, using purposive sampling was conducted by an external consultant, employing a qualitative approach, eight Focus group discussions and 62 Key informant interviews were conducted at four intervention facilities involving beneficiaries, health care providers, health managers and community leaders^4^. Factors related to successfully implementation and ensuring future sustainability were documented.

**Findings:**

Introduction of birth companionship intervention in Katavi region had an immediate impact with notable acceptability and readiness of key stakeholders, such that, beneficiaries were allowed to have a companion during labor and childbirth. Key contributing factors for both implementation success and promising future sustainability noted were (1) Presence of political will (2) Availability of improved provisional infrastructure for audio and visual privacy and trained health care providers (3) Perceived cost-effectiveness of the project (4) Active engagement of community and religious leaders.

**Conclusion:**

The program design and mode of implementation has provided a wide opportunity for sustainability. Furthermore, the design is cost-effective and participatory, future implementation should consider adding accountability mechanism to further strengthen sustainability.

## A99 Perceived outcomes related to birth companionship introduction in Katavi region: selected findings from a qualitative evaluation in Katavi region, April 2021

### Sunday Dominico, Alex Mputa, Agnes Mbanza

#### Thamini Uhai, Dar es salaam, Tanzania

##### **Correspondence:** Sunday Dominico (sdominico@thaminiuhai.or.tz)

**Introduction**

Birth companionship was introduced as a pilot intervention in 2017 by Thamini Uhai in Kigoma region. Following successful implementation, in 2019, Thamini Uhai was able to scale up and introduced implementation in Katavi region in four health centers. Thamini Uhai implemented a 2 years project, to learn about perceived outcomes and benefits of the implementation by key stakeholders.

**Method**

A cross-sectional design, using purposive sampling was conducted by an external consultant, employing a qualitative approach, Eight Focus group discussions and 62 Key informant interviews were conducted at four intervention facilities involving beneficiaries, health care providers, health managers and community leaders^4^. Centered into aspects of quality of care, the outcomes of the project are listed as perceived by key stakeholders in Katavi region.

**Findings**

Introduction of birth companionship intervention in Katavi region had an immediate impact with notable acceptability and readiness of key stakeholders, such that, beneficiaries were allowed to have a companion during labor and childbirth. Key contributing factors for both implementation success and promising future sustainability noted were (1) Improved privacy in labor wards (2) Reduced workload to healthcare providers (3) Improved respectful care to women and by health care providers (4) Improved interaction between women and health care providers and with birth companions (5) Availability/Access to birth companion support (6) Timely detection of complications and (7) increased facility deliveries (was not significant using quantitative data)^1^.

**Conclusion**

Effective implementation of birth companionship contributes to improved maternal and newborn outcomes. Quality clinical care in presence of birth companions ensure supportive and enabling environment for positive experience of labor and delivery.

## A100 Evaluation of point-of-care (POC) HIV exposed infants’ diagnosis (HEID)

### Esther Mtumbuka^1^, Joseph Mziray^1^, Susan Mlangwa^1^, Leah Mtui^1^, Theopista Mbago^1^, David Kayabu^1^, Emmanuel Mwendo^1^, Ambwene Yesaya^2^, Michael Msangi^3^, Mukome Nyamhagata^3^, Debora Kajoka^3^

#### ^1^Clinton Health Access Initiative, Dar es salaam, Tanzania; ^2^National AIDS Control Program, Ministry of Health, Dar es salaam, Tanzania; ^3^National Prevention of Mother To Child Transmission unit, Ministry of Health, Dar es salaam, Tanzania

##### **Correspondence:** Esther Mtumbuka (emtumbuka@clintonHealthAccess.org)

**Introduction**

The WHO strongly recommends early testing of all HIV Exposed Infants (HEI), rapid availability of test results, and prompt initiation of treatment for HIV positive infants. The Point of Care (POC) for HIV Early Infant Diagnosis (HEID) can facilitate same-day diagnosis and rapid ART initiation for HIV-positive infants. Tanzania roll out the use of POC EID 2019 to complement the already

Existing centralized testing network. This study aimed at evaluating POC for HEID implementation in Tanzania to assess its effectiveness in improving timely utilization of results for better clinical management.

**Methodology**

Twenty (20) sites among 54 POC HEID testing implementing sites were randomly selected for retrospective analysis. We used descriptive statistics to compare the outcome for the infants tested between Centralized laboratory testing systems and POC testing system.

**Results**

Data were collected for 1209 infants who received HEID between January and December 2020 from 20 POC EID sites. Testing through conventional and POC platforms was done on 240 (20%) infants and 969 (80%) infants with 3 (1.2%) and 25 (2.6%) HIV-positive infants identified, respectively. POC EID testing improved the turnaround time (TAT) of results from sample collection to results return to caregivers (POC median: 31 days (IQR:14-49) Conventional median: 76 days (IQR: 58-134)). Additionally, POC EID testing improved ART initiation rate for HIV positive infants (POC median: 19 days (IQR:14-49). There was no documented evidence of ART initiation for HIV positive infants diagnosed on conventional platform, hence turnaround time was not calculated.

**Conclusion**

While there may be some implementation challenges that need to be addressed, POC for HEID has been demonstrated to dramatically improve results availability to caregivers for infant initiation on treatment by bring testing services to nearest point of care. However, since POCT does not cover all RCH sites, centralize testing is still important however more improvement is need in referral system to assure return of results to patient management. Furthermore, with over 259 Gene Xpert platform across the country with utilization less than 60%, to maximize the potential benefits of POC EID on rapid ART initiation in resource-limited settings as shown in the study, appropriate mechanisms should be taken to facilitate same day availability of test results to caregivers by utilizing the available platforms.

## A101 Increasing contraception access to adolescents and youth through pharmacies

### Waziri Njau^1^, Charles Mushi^1^, Rose Mzava^1^, Msira Mageni^1^, Lena Mfalila^1^,Tumaini Kiyola^1^, Florence Solomo^2^, Butolwa Bujiku^2^

#### ^1^Johns Hopkins Program for International Education in Gynaecology and Obstetrics, Dar es salaam, Tanzania; ^2^The Ministry of Health, Community Development, Gender, Elderly and Children, Dodoma, Tanzania

##### **Correspondence:** Waziri Njau (Waziri.Njau@jhpiego.org)

**Introduction**

In January 2019, the TCI Tanzania team decided to work with the District Pharmacists in Arusha to improve young people’s experiences when accessing pills and condoms at local pharmacies and Connect them with TCI trained health facilities. Together with TCI, the District Pharmacists identified 300 high-volume registered pharmacies and drugs shops in Arusha that are eligible to receive training ion providing quality family planning counseling particularly young people.. In Tanzania, 37% of modern contraceptive users obtain their method from the private sector, including pharmacies, accredited drug dispensing outlets or shops (SHOPS Plus Project 2018).

**Methods**

Mapping the pharmacies and drug shops who meet the basic criteria and in servinges young people. DPHARMs develop a Memorandum of Understanding (MOU) with interested partners that clearly outlines the pharmacy engagement approach. An MOU is a helpful tool to establish commitment on the part of the pharmacists and drug shop owners. ADDOs established a referral system whereby clients are referred to the closest TCI-supported HFs.

**Results**

TCI trained drugs shop sellers have provided 422 condoms and 223 pills to clients and referred 1,812 clients to TCI health facilities. 55% of the clients referred to health facilities were youth between the ages of 15-24. TCI health facilities have completed 636 referrals from pharmacies since the partnership began, leading to a 20% increase in youth acceptors between April 2018 and 2019.

**Conclusion**

ADDOs program had contributed in mCPR increase and referrals among youths to the near facility, this will help to strengthen youth friendly services. ADDO program reduces effects of misuse of emergency contraceptive pills and uterotonic drugs among youth of reproductive age. Pharmacy and drug shops sellers are now more motivated to counsel on contraceptives and are sharing user data on time with the District Pharmacists. The District Pharmacists are also motivated to ensure that the monthly data is collected.

